# p75 neurotrophin receptor modulation in mild to moderate Alzheimer disease: a randomized, placebo-controlled phase 2a trial

**DOI:** 10.1038/s41591-024-02977-w

**Published:** 2024-05-17

**Authors:** Hayley R. C. Shanks, Kewei Chen, Eric M. Reiman, Kaj Blennow, Jeffrey L. Cummings, Stephen M. Massa, Frank M. Longo, Anne Börjesson-Hanson, Manfred Windisch, Taylor W. Schmitz

**Affiliations:** 1 Schulich School of Medicine and Dentistry, Western University, London, Ontario Canada; 2 Robarts Research Institute, Western University, London, Ontario Canada; 3 Western Institute for Neuroscience, Western University, London, Ontario Canada; 4 Banner Alzheimer’s Institute, Phoenix, AZ USA; 5 College of Medicine-Phoenix, University of Arizona, Phoenix, AZ USA; 6 College of Health Solutions, Arizona State University, Downtown, Phoenix, AZ USA; 7 Translational Genomics Research Institute, Phoenix, AZ USA; 8 Arizona Alzheimer’s Consortium, Phoenix, AZ USA; 9 ASU-Banner Neurodegenerative Disease Research Center, Arizona State University, Tempe, AZ USA; 10 Department of Psychiatry and Neurochemistry, Institute of Neuroscience and Physiology, Sahlgrenska Academy, University of Gothenburg, Mölndal, Sweden; 11 Clinical Neurochemistry Laboratory, Sahlgrenska University Hospital, Mölndal, Sweden; 12 Chambers-Grundy Center for Transformative Neuroscience, Department of Brain Health, School of Integrated Health Sciences, University of Nevada Las Vegas (UNLV), Las Vegas, NV USA; 13 San Francisco Veterans Affairs Health Care System, San Francisco, CA USA; 14 Department of Neurology, University of California, San Francisco, San Francisco, CA USA; 15 PharmatrophiX, Inc., Menlo Park, CA USA; 16 Clinical Trials, Department of Aging, Karolinska University Hospital, Stockholm, Sweden; 17 NeuroScios, GmbH, St. Radegund, Austria

**Keywords:** Alzheimer's disease, Neurotrophic factors, Drug safety, Alzheimer's disease

## Abstract

p75 neurotrophin receptor (p75^NTR^) signaling pathways substantially overlap with degenerative networks active in Alzheimer disease (AD). Modulation of p75^NTR^ with the first-in-class small molecule LM11A-31 mitigates amyloid-induced and pathological tau-induced synaptic loss in preclinical models. Here we conducted a 26-week randomized, placebo-controlled, double-blinded phase 2a safety and exploratory endpoint trial of LM11A-31 in 242 participants with mild to moderate AD with three arms: placebo, 200 mg LM11A-31 and 400 mg LM11A-31, administered twice daily by oral capsules. This trial met its primary endpoint of safety and tolerability. Within the prespecified secondary and exploratory outcome domains (structural magnetic resonance imaging, fluorodeoxyglucose positron-emission tomography and cerebrospinal fluid biomarkers), significant drug–placebo differences were found, consistent with the hypothesis that LM11A-31 slows progression of pathophysiological features of AD; no significant effect of active treatment was observed on cognitive tests. Together, these results suggest that targeting p75^NTR^ with LM11A-31 warrants further investigation in larger-scale clinical trials of longer duration. EU Clinical Trials registration: 2015-005263-16; ClinicalTrials.gov registration: NCT03069014.

## Abstract (web summary)

A phase 2a trial of LM11A-31 in mild to moderate Alzheimer disease suggests that p75^NTR^ modulation is safe and attenuates measures of degeneration.

## Main

Late-onset Alzheimer disease (AD) is the leading cause of dementia^1,2^. AD is a complex and heterogeneous disease in which multiple mechanisms become dysregulated to promote synaptic failure, degeneration and loss^3,4^. Two important approaches for disease-modifying AD therapies involve targeting the accumulation of pathological forms of amyloid-β (Aβ) or tau^5–8^. A limitation of these strategies is that they each target a narrow set of AD-related pathophysiological processes. An alternative pharmacological strategy is to target ‘deep biology’, that is, receptors and/or signaling networks that control manifold fundamental cellular pathways and may, therefore, be able to normalize multiple pathological processes underlying AD, particularly those relevant to synaptic resilience and degeneration^9–11^.

Over the past two decades, multiple lines of evidence have converged on the p75 neurotrophin receptor (p75^NTR^) as a promising deep biology target for modifying neuronal dysfunction and degeneration in AD. p75^NTR^ is a member of the tumor necrosis factor family^12^. Although p75^NTR^ has traditionally been known as a ‘death receptor’, more recent work has demonstrated that it can determine synaptic and cellular fate^13^. p75^NTR^ is a coreceptor for sortilin and SorCS2. In its nonliganded state or when binding to proneurotrophin ligands, such as pro-nerve growth factor (pro-NGF) or pro-brain-derived neurotrophic factor (pro-BDNF), p75^NTR^ promotes degenerative signaling that causes destabilization of dendritic spines, degeneration of synapses and neuronal death^14–17^. However, p75^NTR^ can also bind mature forms of neurotrophins (such as NGF and BDNF) and can act as a tropomyosin receptor kinase (Trk) co-receptor, thereby promoting cell survival and synaptic plasticity through multiple pathways^18–20^. Thus, p75^NTR^ acts as a potent and fundamental molecular signal switch for neuronal survival and synaptic integrity.

p75^NTR^ regulates a broad intracellular signaling network that has considerable overlap with degenerative signaling networks active in AD, particularly those relevant to synaptic function and resilience^18,21–23^. Consistent with this overlap, p75^NTR^-mutant mice demonstrate resilience against Aβ-related neuronal degeneration^17,24–28^. In humans, polymorphisms in the genes encoding proneurotrophins and p75^NTR^ coreceptors, including sortilin and SorCS2, are associated with altered AD risk^29–32^. Studies of patients with AD and tauopathy reported increased levels of p75^NTR^ in brain tissue and elevated levels of pro-NGF in brain extracts and cerebrospinal fluid (CSF)^33–36^. In the adult human brain, the highest expression of p75^NTR^ is observed in cell types that are among the earliest affected in AD, including cholinergic neurons of the basal forebrain and their cholinoreceptive target populations in the entorhinal cortex and hippocampus^13,37,38^. p75^NTR^ is also expressed by cortical, hippocampal pyramidal and locus coeruleus neurons, with locus coeruleus neurons constituting another population involved in the earliest stages of AD pathology^39^. Within non-neuronal populations, p75^NTR^ expression is upregulated in microglia and astrocytes in pathological settings, including AD and tauopathies^15^.

Taken together, these lines of evidence have motivated preclinical work examining the therapeutic potential for small-molecule modulation of p75^NTR^ to downregulate its degenerative signaling^40^. One such candidate, LM11A-31, is a small molecule based on the structural, physical and chemical features of β-hairpin loop 1 of NGF, a domain of NGF that mediates interaction with p75^NTR^ (ref. ^41^). LM11A-31 functions as a p75^NTR^ modulator to downregulate its degenerative signaling and as an antagonist to pro-NGF-induced degeneration^42,43^. It was found to readily cross the blood–brain barrier following oral administration and was nontoxic in preclinical studies^44,45^. In AD and tauopathy mouse models, oral administration of LM11A-31 reduced excess activation of enzymes contributing to tau post-translational modifications, accumulation of multiple forms of pathological tau species and tau seeding activity, reduced elevations in multiple microglia and astrocyte markers, and decreased the loss of dendritic spines and synapses while improving performance on hippocampal-dependent memory tasks^45,46^. In β-amyloid precursor protein (APP)-transgenic mice, administration of LM11A-31 had no detectable effect on Aβ plaques or brain tissue-derived soluble Aβ levels^47^. These findings, along with in vitro studies demonstrating that LM11A-31 inhibits neurite and synaptic degeneration induced by oligomeric Aβ^43^, suggest that modulation of p75^NTR^ confers resilience to Aβ.

Despite its fundamental functional role in neural and developmental cell biology, the therapeutic potential for targeted modulation of p75^NTR^ in humans has not been tested. In this study, we report the application of a p75^NTR^-based therapy in a human disease setting through a 26-week randomized, double-blind, parallel-group phase 2a safety and exploratory efficacy trial of LM11A-31 in participants with mild to moderate AD dementia. On the basis of studies in preclinical AD-related mouse models and two prior safety and pharmacokinetic studies in healthy human participants (designated as phase 1 and 1b trials), we hypothesized that modulation of p75^NTR^ using LM11A-31 in persons with AD would be well tolerated and would slow AD progression, as measured by biomarkers of synaptic function, degeneration and glial activation (CSF biomarkers, structural magnetic resonance imaging (sMRI) and [^18^F]-fluorodeoxyglucose positron-emission tomography ([^18^F]-FDG PET)). Consistent with phase 2a strategies in AD trials^48^, cognitive measures were included as secondary or exploratory outcomes for assessment of safety and nominal directionality; the study was not of sufficient duration or power to reliably assess effects on potentially slowing the loss of cognitive function.

## Results

### Participant disposition

**Fig. 1:**
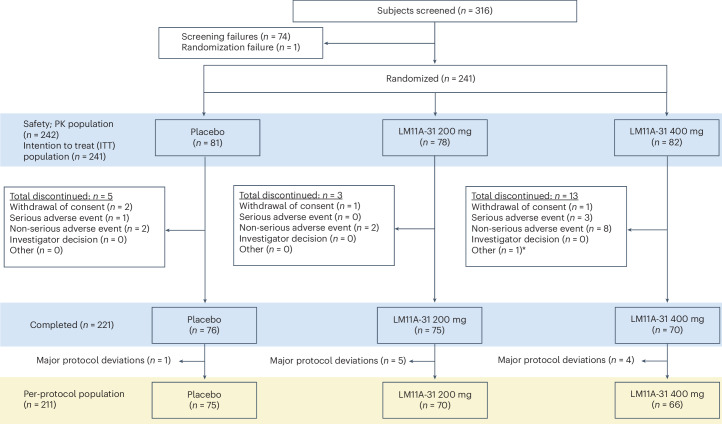
Participant flow diagram for the phase 2a LM11A-31 clinical trial. Examples of major protocol violations included failure to meet inclusion or exclusion criteria (data changed or violation was not detected before dosing), use of prohibited medication that began during the treatment period (Methods), incomplete treatment (<80% compliance over the treatment period), final visit outside prespecified acceptable visit window (182 ± 7 days after baseline visit) or early withdrawal. PK population, pharmacokinetic study population. *Discontinued due to randomization failure.

A total of 316 participants were screened for inclusion; 242 were enrolled in the trial (safety population) and 241 were successfully randomized and accounted for in the intention-to-treat (ITT) population. The first participant was randomized in May 2017 and the last participant completed treatment in June 2020. Data lock was executed in November 2020. Of these individuals, 221 completed the study as outlined in the protocol and 211 completed the study at the 26-week visit (Fig. 1). Analyses of primary, secondary, prespecified exploratory and post hoc exploratory outcomes were based on the ITT dataset.

Baseline characteristics of the trial cohort are outlined in Table 1. All trial participants had a biologically confirmed AD diagnosis (CSF Aβ42 < 550 ng l^−1^ or ratio of Aβ42 to Aβ40 < 0.89). Participants in the twice-daily placebo, 200 mg LM11A-31 and 400 mg LM11A-31 groups did not differ with respect to any key subject variables such as age, sex, race, screening Mini Mental Status Exam (MMSE) score, screening CSF Aβ42 or use of acetylcholinesterase (AChE) inhibitors (AChEIs) (*P* > 0.1 for each; Table 1). There was a slightly higher proportion of carriers of pathogenic apolipoprotein E4 (*APOE4*) alleles in the 400-mg group, although differences between groups did not reach statistical significance (*P* = 0.09).

**Table 1:** Demographic and clinical characteristics of safety population

	Placebo	200 mg	400 mg	Statistic	*P* value
*n*	81	78	83		
Age	72 (8.00)	72 (7.75)	72 (8.00)	*H* = 0.81	0.67
Males, *n* (%)	35 (43.2)	38 (48.7)	40 (48.2)	*χ*^*2*^ = 0.60	0.74
Race white, *n* (%)	81 (100)	78 (100)	83 (100)		
*APOE4* alleles (0/1/2)	34/39/8	37/26/15	30/44/9	*χ*^*2*^ = 8.12	0.09
Screening MMSE	22 (4.00)	22 (5.00)	23 (4.00)	*H* = 3.19	0.20
Screening Aβ42	511 (217)	489 (263)	568 (244)	*H* = 1.67	0.43
Using AChEIs	75 (92.6)	67 (85.9)	76 (91.6)	*χ*^*2*^ = 0.83	0.66

Continuous data are represented as the median (interquartile range) and categorical data are represented as the number of participants (percentage), unless otherwise specified. Chi-squared tests were used to assess differences in categorical variables and two-sided nonparametric ANOVAs (Kruskal–Wallis tests) were used for continuous variables.

### Primary outcome

This study reports the effects of the novel strategy of selectively targeting p75^NTR^ in a human population with disease. Moreover, LM11A-31 constitutes a first-in-class therapeutic agent for p75^NTR^. As such, evaluation of safety and tolerability was of key importance. The study met its primary prespecified endpoint of demonstrating the safety and tolerability of LM11A-31.

In order, the most frequently observed adverse events (AEs) were nasopharyngitis, diarrhea, headache and eosinophilia (Table 2). In most cases, AEs were transient. Nasopharyngitis (17 participants) and diarrhea (13 participants) were significantly more commonly reported in the 400 mg LM11A-31 group compared to placebo (odds ratio (OR) with 95% confidence interval (CI): nasopharyngitis, 5.41 (1.15 to 25.52); diarrhea, 12.22 (1.54 to 97.00); *P* < 0.05 for each). Of these participants, two withdrew due to diarrhea and none withdrew due to nasopharyngitis. Headache was experienced by a total of 12 participants, with two in the placebo group, five in the 200-mg group and five in the 400-mg group (2.53 (0.48 to 13.44)). There were no discontinuations due to headache. There were more total discontinuations in the 400-mg group (12 participants) than in the 200-mg (3 participants) and placebo (5 participants) groups.

**Table 2:** Safety of LM11A-31 in mild to moderate AD

Category		Placebo (*n* = 81)			200 mg LM11A-31 (*n* = 78)			400 mg LM11A-31 (*n* = 83)		
		*n*	%	freq	*n*	%	freq	*n*	%	freq
All AEs		42	51.9	100	47	60.3	109	55	66.3	185
Pretreatment signs and symptoms		7	8.6	9	8	10.3	9	8	9.6	12
TEAEs		41	50.6	91	46	59.0	100	54	65.1	173
Drug relationship	Related	8	9.9	11	12	15.4	15	13	15.7	23
	Not related	36	44.4	80	41	52.6	85	48	57.8	150
Intensity	Mild	32	39.5	69	40	51.3	86	46	55.4	119
	Moderate	11	13.6	19	11	14.1	12	24	28.9	49
	Severe	3	3.7	3	2	2.6	2	5	6.0	5
AE leading to temporary discontinuation		4	4.9	9	5	6.4	5	15	18.1	20
AE leading to permanent discontinuation		3	3.7	3	2	2.6	2	11	13.3	11
SAEs		4	4.9	4	2	2.6	2	7	8.4	7
AE leading to death		1	1.2	1	0	0.0	0	0	0.0	0
Most common TEAEs										
Nasopharyngitis		2	2.5	2	5	6.4	5	10	12.0	16
Diarrhea		1	1.2	1	1	1.3	1	11	13.3	15
Headache		2	2.5	3	5	6.4	6	5	6.0	17
Eosinophilia		0	0.0	0	5	6.4	5	5	6.0	6

*n*, the number of participants exhibiting an event; freq, the total number of events (multiple events may occur per participant); TEAE, treatment-emergent AE.

Eosinophilia occurred in ten participants, with five in the 200-mg group and five in the 400-mg group. Of these ten participants, three were permanently removed from the study. The study drug was discontinued temporarily in two participants. Eosinophil increases were asymptomatic and none were classified as serious AEs (SAEs). Four participants exhibited eosinophil increases to levels greater than 500 per mm^3^ above baseline. These values resolved to within the normal range by each participant’s next scheduled visit with a time range of approximately 1 month. In the six participants with lower levels of eosinophil elevation, four were found to return to a normal level at the 1-month follow-up and two participants discontinued the study before follow-up laboratory testing. Eosinophilia did not occur in the placebo group.

A total of 33 participants (14%) experienced AEs considered to be related to the study medications. Of these participants, 8 (10%) received placebo, 12 (15%) received 200 mg LM11A-31 and 13 (16%) received 400 mg LM11A-31 (Table 2). A total of 15 SAEs occurred in the study across 15 participants. Of these participants, two experienced an SAE before dosing and were considered screening failures. Of the remaining participants, four were in the placebo group, two were in the 200-mg group and seven were in the 400-mg group. One SAE (gastrointestinal bleeding) occurred after 16 consecutive days of dosing and was classified as possibly being related to LM11A-31 treatment. This participant withdrew from the study, was found by endoscopic exam to have a gastric ulcer of unknown duration and fully recovered. No other gastrointestinal bleeding was reported in the study.

Within the ITT population, the study medication was discontinued in 20 participants in total. Reasons for discontinuation were AEs (12 participants; placebo, *n* = 2; 200 mg LM11A-31, *n* = 2; 400 mg LM11A-31, *n* = 8), SAEs (4 participants; placebo, *n* = 1; 400 mg LM11A-31, *n* = 3) and withdrawal of consent (4 participants; placebo, *n* = 2; 200 mg LM11A-31, *n* = 1; 400 mg LM11A-31, *n* = 1). The most common reason for discontinuing the study was gastrointestinal symptoms (seven participants; placebo, *n* = 1; 200 mg LM11A-31, *n* = 1; 400 mg LM11A-31, *n* = 5) followed by eosinophilia (three participants; 200 mg LM11A-31, *n* = 1; 400 mg LM11A-31, *n* = 2). One participant died during the trial. This participant was in the placebo group and cause of death was pancreatic adenocarcinoma.

No significant abnormalities within the placebo or LM11A-31 groups were identified for participant vital signs (blood pressure, heart rate, respiratory rate and body temperature), 12-lead electrocardiogram or clinical laboratory assessment (hematology, biochemistry, coagulation, serology and urinalysis). MRI did not detect findings that raised concern regarding drug safety, including amyloid-related imaging abnormalities (ARIAs).

Assessment with the Columbia Suicide Severity Rating Scale detected no differences among treatment groups (*P* > 0.1).

Given that p75^NTR^ may affect the vascular system^49,50^, it was of particular interest to analyze systolic and diastolic blood pressure values across the three treatment groups. No significant differences in these measures at screening were observed across the three groups (*P*_Kruskal__–__Wallis_ > 0.1 for each). No significant longitudinal differences in systolic blood pressure were observed with a Kruskal–Wallis test (*P* = 0.691). Longitudinal changes in diastolic blood pressure differed significantly among the three groups (*P* = 0.036). The median change in diastolic blood pressure was +1 mm Hg in the placebo group, 0 mm Hg in the 200 mg LM11A-31 group and −2 mm Hg in the 400 mg LM11A-31 group. Post hoc testing with Dunn’s test revealed that the median longitudinal change in diastolic blood pressure was significantly different in the 400 mg LM11A-31 group compared to the placebo group (*P* = 0.010). No other significant differences were detected among groups. The magnitude of longitudinal change in diastolic blood pressure was not clinically significant.

In all, the Data Safety Monitoring Board concluded that LM11A-31 caused no overall safety concerns and its safety profile was compatible with future larger-scale testing. Thus, the primary trial endpoint of safety was met.

### Analysis of secondary and exploratory outcomes

**Extended Data Fig. 1:**
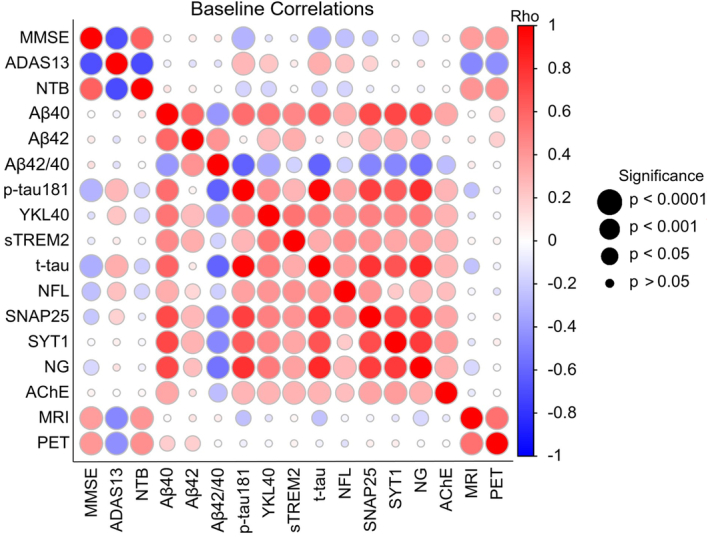
Baseline correlations between biomarker and cognitive endpoints. Correlation matrix showing relationships between cognitive and biomarker measures before treatment (baseline) across all trial participants. The number of participants with data for each variable ranged from 194 to 241 and includes participants regardless of treatment group. Outliers were defined for each variable as datapoints which were more than three median absolute deviations from the median. These values were excluded from correlations. Circles in the correlation matrix represent Spearman’s Rho and are scaled in size based on the significance of the Spearman correlation. All *P* values are uncorrected and statistics were performed two-sided. Aβ42/40, ratio of Aβ42 to Aβ40; ADAS, ADAS-Cog-13.

All secondary endpoints were prespecified in the registrations (EU Clinical Trials: 2015-005263-16; ClinicalTrials.gov: NCT03069014). Prespecified exploratory outcome measures were determined on the basis of the results of preclinical studies^43,46,47,51^ and were described in the statistical analysis plan. Before assessing longitudinal treatment effects on the secondary and the prespecified exploratory outcome measures, we assessed the baseline characteristics of the clinical trial cohort on these measures. To do so, we computed pairwise Spearman correlations among CSF, imaging and cognitive data across all participants at baseline (Extended Data Fig. 1). Overall, the baseline interrelationships among the secondary and prespecified exploratory measures broadly recapitulated those found in prior AD biomarker studies^52–54^.

**Extended Data Fig. 2:**
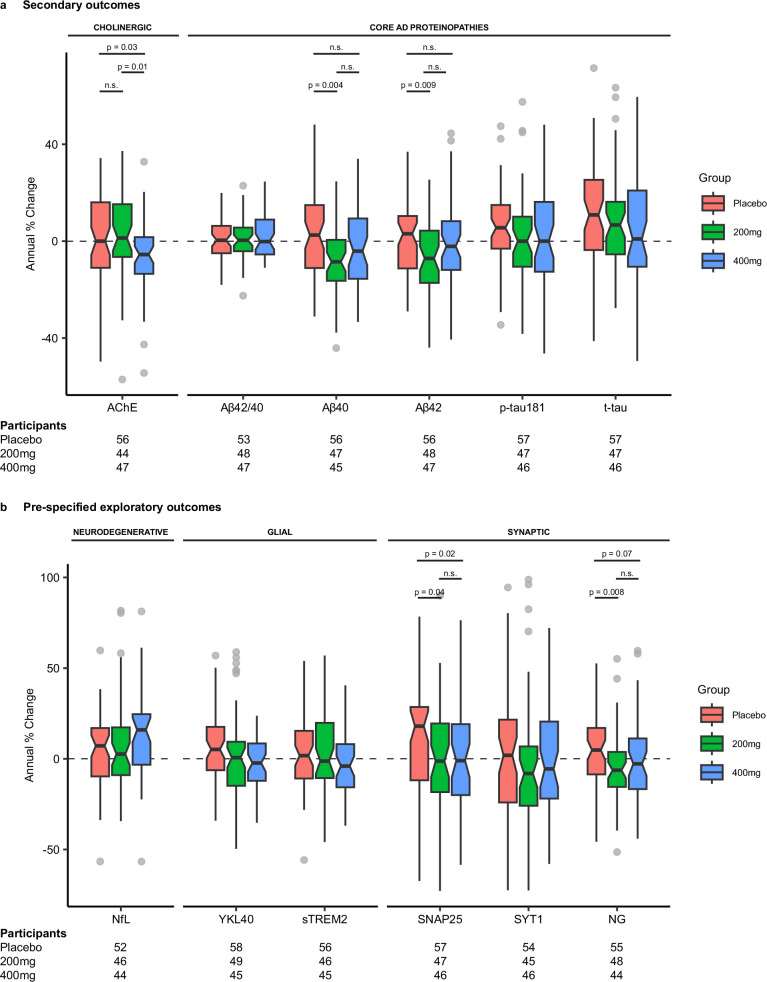
Longitudinal changes in CSF biomarkers across treatment arms. Box plots showing the annual percent change of **a**) secondary and **b**) pre-specified exploratory CSF biomarkers in the placebo (salmon), 200 mg LM11A-31 (green) and 400 mg LM11A-31 (blue) groups. Horizontal lines on box plots represent the median of the distribution. Notches provide 95% confidence intervals of the median, which represent the reliability of within-group change. The lower and upper hinges of the boxplot correspond to the first and third quartiles of the distribution, and the whiskers (vertical lines) extend from the hinge to the largest or smallest value, no further than ±1.5 times the interquartile range from the hinge. Significant Kruskal-Wallis tests (*P*_AChE_ = 0.029; *P*_Aβ40_ = 0.015; *P*_Aβ42_ = 0.032; *P*_SNAP25_ = 0.035; *P*_NG_ = 0.024) were followed by post hoc Dunn’s tests to determine which groups differed significantly from each other. The number of participants included in each statistical test varied due to availability of test results for a given subject and variation in outlier number (3-12 per variable across all trial participants). Sample sizes are indicated below each boxplot. All statistics were performed two-sided and were not adjusted for multiple comparisons, given the exploratory nature of the study. Aβ42/40, ratio of Aβ42 to Aβ40.

**Extended Data Fig. 3:**
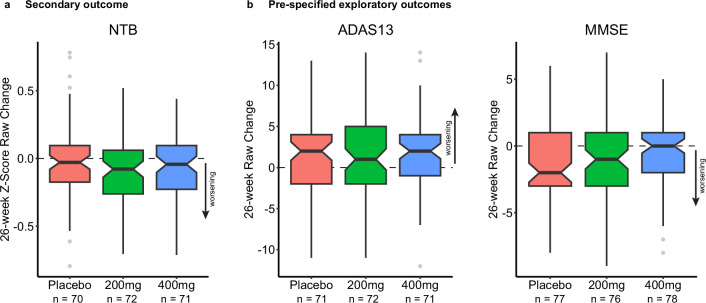
26-week changes in cognitive test scores by dose group. Box plots showing the change in score between the first and last assessment on the **a**) NTB z-score, **b)** ADAS-Cog-13 (ADAS13), and **c**) MMSE in the placebo (salmon), 200 mg LM11A-31 (green) and 400 mg LM11A-31 (blue) groups. Note that y axes are scaled differently in each panel. Horizontal lines on box plots represent the median of the distribution. Notches provide 95% confidence intervals of the median. The lower and upper hinges of the boxplot correspond to the first and third quartiles of the distribution, and the whiskers (vertical lines) extend from the hinge to the largest or smallest value, no further than ±1.5 times the interquartile range from the hinge. Kruskal-Wallis tests did not detect a significant difference between the three groups for any cognitive test (*P*_NTB_ = 0.346; *P*_ADAS_ = 0.964; *P*_MMSE_ = 0.651). All statistics were performed two-sided and were not adjusted for multiple comparisons, given the exploratory nature of the study. ADAS, ADAS-Cog-13.

**Extended Data Fig. 4:**
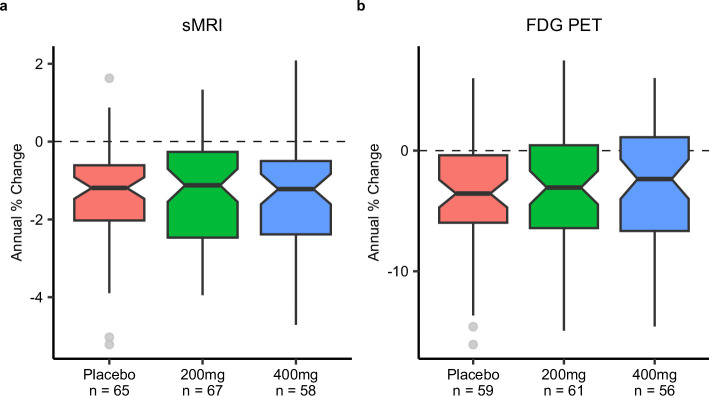
Longitudinal changes in neuroimaging data by dose group. Longitudinal changes in grey matter volume (**a**) or [^18^F]-FDG PET SUVr (**b**) in the placebo (salmon), 200 mg LM11A-31 (green) and 400 mg LM11A-31 (blue) groups within AD-vulnerability region of interest masks (Extended Data Fig. 6). Note that y axes are scaled differently in each panel. Horizontal lines on box plots represent the median of the distribution. Notches provide 95% confidence intervals of the median. The lower and upper hinges of the boxplot correspond to the first and third quartiles of the distribution, and the whiskers (vertical lines) extend from the hinge to the largest or smallest value, no further than ±1.5 times the interquartile range from the hinge. Two-sided Kruskal-Wallis tests did not detect a significant difference between the three groups for MRI (*P* = 0.847) or PET (*P* = 0.649). The number of participants included in each statistical test varied due to availability of test results for a given subject and variation in outlier number. Given the exploratory nature of the study, *P* values were not corrected for multiple comparisons.

Having characterized the relationships among CSF biomarkers, neuroimaging biomarkers and clinical tests at baseline, we next examined whether longitudinal changes differed between placebo and LM11A-31. Results from preclinical studies and a prior phase 1b safety and CSF pharmacokinetic trial (F.M.L., unpublished data) suggest that both doses of LM11A-31 (200 mg and 400 mg twice daily) included in the present trial would reach brain exposure levels consistent with engagement of p75^NTR^-related mechanisms. Consistent with these observations, longitudinal changes in CSF, imaging region-of-interest analyses and cognitive tests did not differ between the two dose arms for 16 of the 17 variables assessed (Extended Data Table 1). Analyses of secondary and exploratory endpoints by dose group are presented in Extended Data Figs. 2–4. For further secondary and exploratory data analyses, we pooled participants from the 200-mg and 400-mg arms into a single LM11A-31 group.

For the analysis of secondary and prespecified exploratory endpoints, longitudinal changes in CSF biomarkers were quantified using an annual percent change calculation^55^ (Methods). Significant differences in median change between placebo and LM11A-31 groups were investigated using Wilcoxon rank sum tests with 95% bootstrap CIs from 5,000 bootstrap iterations.

### Secondary outcomes

**Fig. 2:**
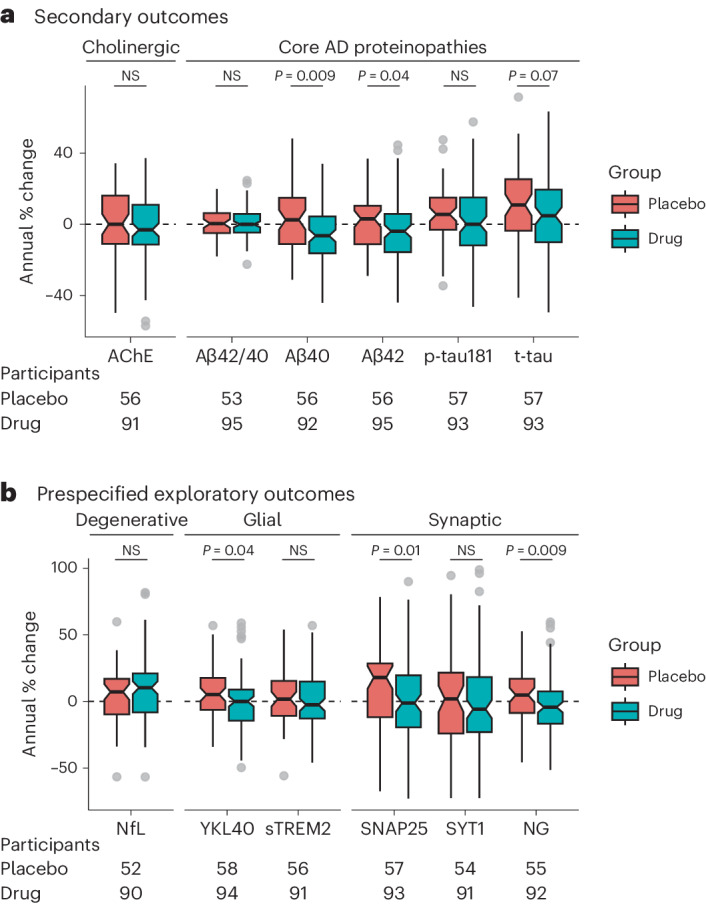
Secondary and prespecified exploratory CSF biomarker endpoints. **a**,**b**, Box plots show the annual percent change values of secondary (**a**) and prespecified exploratory (**b**) CSF biomarkers in the placebo group (salmon) and the drug group (teal). Black horizontal lines in the box plots represent the median of each distribution. Notches provide the 95% CIs of the median, which represent the reliability of within-group change. The lower and upper hinges of the box plot correspond to the first and third quartiles of the distribution and the whiskers (vertical lines) extend from the hinge to the largest or smallest value, no further than ±1.5 times the interquartile range from the hinge. Two-sided Wilcoxon rank sum tests were used to compare longitudinal changes in the placebo and LM11A-31 groups. Participant numbers across the groups vary due to the availability of test results for a given participant and variation in the outlier number (3–12 per variable across all trial participants). The number of participants included in each comparison is presented below each box plot. Given the exploratory nature of the trial, all *P* values are uncorrected. NS, not significant; Aβ42/40, ratio of Aβ42 to Aβ40.

Secondary CSF outcomes consisted of the following core AD biomarkers: total tau (t-tau), tau phosphorylated at Thr181 (p-tau181), Aβ40 and Aβ42. LM11A-31 significantly slowed longitudinal increases in Aβ42 compared to placebo (Fig. 2a; *P*_rank sum_ = 0.037). The difference in median annual percent change of Aβ42 in the LM11A-31 group relative to the placebo group was −6.98% (95% CI, −14.22% to −1.45%). LM11A-31 also significantly slowed longitudinal increases in CSF Aβ40 compared to placebo (Fig. 2a; *P*_rank sum_ = 0.009). The difference in median annual percent change of Aβ40 in the LM11A-31 group relative to the placebo group was −8.96% (95% CI, −17.60% to −1.29%). Longitudinal changes in the ratio of Aβ42 to Aβ40 between the two groups were not significantly different (*P*_rank sum_ = 0.952). The difference in median annual percent change of the ratio of Aβ42 to Aβ40 between LM11A-31 and placebo was −0.42 (95% CI, −2.90% to 2.49%). Overall, these findings indicate that longitudinal AD-related increases in CSF Aβ42 and Aβ40 were slowed or reversed by LM11A-31, while the ratio of Aβ42 to Aβ40 was unaffected.

Longitudinal changes in CSF p-tau181 between the LM11A-31 and placebo groups were not significantly different (Fig. 2a; *P*_rank sum_ = 0.201). The difference in median annual percent change between LM11A-31 and placebo was −5.54% (95% CI, −12.60% to 1.17%). Longitudinal changes in CSF t-tau between the LM11A-31 and placebo groups were not significantly different (Fig. 2a; *P*_rank sum_ = 0.068). The difference in median annual percent change between LM11A-31 and placebo for t-tau was −6.07% (95% CI, −17.45% to 2.71%).

Given the high expression of p75^NTR^ by basal forebrain cholinergic neurons, CSF AChE activity was additionally measured. Longitudinal changes in AChE activity did not differ between placebo and LM11A-31 (*P*_rank sum_ = 0.295). The difference in median annual percent change of AChE activity between placebo and LM11A-31 was −3.12% (95% CI, −10.52% to 3.30%).

**Fig. 3:**
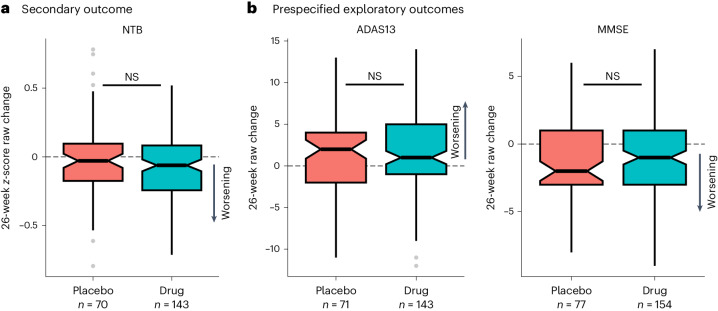
Secondary and prespecified exploratory cognitive measures under placebo and LM11A-31. **a**,**b**, Box plots showing the change in score between the first and last assessments on the NTB *z*-score (**a**), ADAS-Cog-13 (left) and MMSE (right) (**b**) in the pooled LM11A-31 (teal) and placebo (salmon) groups. Note that *y* axes are scaled differently in each panel. Horizontal lines on box plots represent the median of the distribution. Notches provide the 95% CIs of the median, which represent the reliability of within-group change. The lower and upper hinges of the box plot correspond to the first and third quartiles of the distribution and the whiskers (vertical lines) extend from the hinge to the largest or smallest value, no further than ±1.5 times the interquartile range from the hinge. Differences between the drug and placebo groups were not significant using a two-sided Wilcoxon rank sum test for any cognitive test (*P*_NTB_ = 0.185; *P*_ADAS_ = 0.789; *P*_MMSE_ = 0.492). Given the exploratory nature of the trial, all *P* values are uncorrected. ADAS13, ADAS-Cog-13.

**Extended Data Fig. 5:**
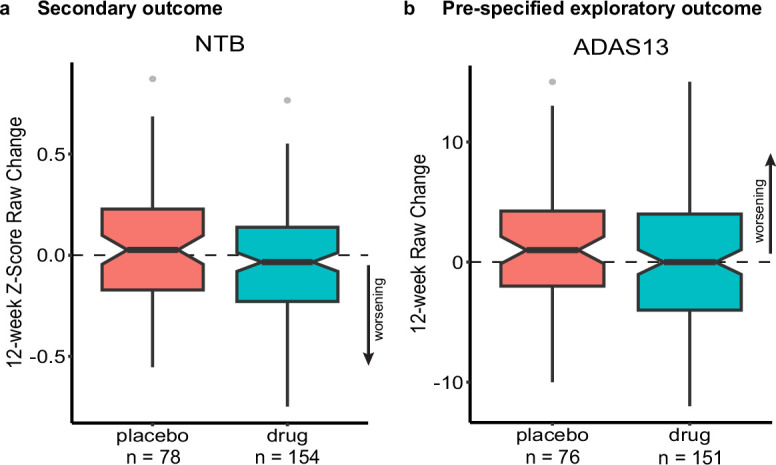
Longitudinal changes in cognitive data at 12-weeks. Box plots show the raw change in cognitive test scores between study baseline and the 12-week visit in the placebo (salmon) and LM11A-31 (teal) groups. Black horizontal lines on the box plots represent the median of the distribution. Notches provide 95% confidence intervals of the median. The lower and upper hinges of the boxplot correspond to the first and third quartiles of the distribution, and the whiskers (vertical lines) extend from the hinge to the largest or smallest value, no further than ±1.5 times the interquartile range from the hinge. **a**) The between-group difference in longitudinal cognitive decline on the NTB was not significant (*P* = 0.156) according to a Wilcoxon rank sum test. **b**) Placebo and drug groups did not differ in their median change scores on the ADAS-Cog-13 (Wilcoxon rank sum *P* = 0.422). All statistics were performed two-sided and were not adjusted for multiple comparisons, given the exploratory nature of the study. ADAS13, ADAS-Cog-13.

While the relatively low power and short duration of the study limited assessment of cognitive and other clinical effects^48^, we determined whether administration of LM11A-31 was associated with potential interval directionality of cognition. The secondary cognitive outcome measure in the trial was a custom neuropsychological test battery (NTB; Methods) that was collected at study baseline, 12 weeks and 26 weeks. The placebo and LM11A-31 groups did not differ in longitudinal cognitive decline on the NTB global *z*-score at 12 weeks (*P*_rank sum_ = 0.156; Extended Data Fig. 5) or 26 weeks (*P*_rank sum_ = 0.185; Fig. 3a). The difference in median change on NTB global *z*-score between the LM11A-31 and placebo groups was −0.06 (95% CI, −0.14 to 0.05) at 12 weeks and −0.03 (95% CI, −0.10 to 0.04) at 26 weeks.

### Exploratory outcomes

Prespecified exploratory CSF biomarkers collected in the trial can be broadly grouped into three domains: (1) synaptic biomarkers, including synaptosomal associated protein 25 (SNAP25), synaptotagmin 1 (SYT1) and neurogranin (NG); (2) the neurodegenerative biomarker neuron-specific intermediate filament neurofilament light chain (NfL); and (3) glial biomarkers, including chitinase-3-like protein 1, also known as YKL40, and soluble triggering receptor expressed on myeloid cells 2 (sTREM2).

Longitudinal analysis of progression of exploratory endpoints of synaptic degeneration focused on CSF SNAP25, SYT1 and NG (Fig. 2b). LM11A-31 significantly slowed longitudinal increases in the presynaptic SNAP25 biomarker compared to placebo (Fig. 2b; *P*_rank sum_ = 0.010). The difference in median annual percent change between LM11A-31 and placebo for SNAP25 was −19.20% (95% CI, −32.19% to −1.47%). The annual percent change of the presynaptic marker SYT1 did not differ significantly between the placebo and LM11A-31 groups (*P*_rank sum_ = 0.426). The difference in median annual percent change of SYT1 between placebo and LM11A-31 was −7.76% (95% CI, −20.13% to 4.99%). LM11A-31 also significantly slowed longitudinal increases in the postsynaptic NG biomarker compared to placebo (Fig. 2b; *P*_rank sum_ = 0.009). The difference in median annual percent change between LM11A-31 and placebo for NG was −9.17% (95% CI, −16.32% to −2.35%). These results suggest that LM11A-31 slows progression of presynaptic and postsynaptic loss, as measured by CSF SNAP25 and NG.

Longitudinal changes in CSF NfL between the LM11A-31 and placebo groups were not significantly different (Fig. 2b; *P*_rank sum_ = 0.315). The difference in median annual percent change between LM11A-31 and placebo for NfL was 3.13% (95% CI, −8.64% to 16.31%).

Longitudinal analysis of glial activation focused on CSF YKL40 and sTREM2. LM11A-31 significantly slowed longitudinal increases in YKL40 compared to placebo (Fig. 2b; *P*_rank sum_ = 0.040). The difference in median annual percent change between LM11A-31 and placebo was −5.19% (95% CI, −14.80% to 2.49%). Lastly, the median annual percent change of sTREM2 in the placebo and LM11A-31 groups did not differ significantly, with a median difference of −4.29% (95% CI, −13.12% to 3.15%; *P*_rank sum_ = 0.172).

Prespecified exploratory cognitive outcomes included global scores on the AD Assessment Scale—Cognitive Subscale (ADAS-Cog-13) and MMSE. ADAS-Cog-13 testing was performed at baseline, at the 12-week time point and at the conclusion of treatment, while the MMSE was performed only before treatment and at the conclusion of treatment (Extended Data Table 2). In addition, we acquired the clinical global impression test (CGI) and a computer-based simulation of Morris water maze testing (Amunet)^56,57^. Collection schedules for cognitive and clinical tests are detailed in Extended Data Table 2.

We observed median decreases of two points on the MMSE, as well as a median increase of two points on the ADAS-Cog-13, in the trial placebo group over the 26-week trial. The magnitudes of these longitudinal changes are consistent with the rate of impairment observed across multiple trial placebo groups in populations with mild to moderate AD^58^. No significant differences in longitudinal cognitive decline were detected between the placebo and LM11A-31 groups on the MMSE or ADAS-Cog-13 at 12 weeks (Extended Data Fig. 5) or 26 weeks (Fig. 3; *P*_rank sum_ > 0.1 for all). For the ADAS-Cog13, the difference in median change between LM11A-31 and placebo was −1 (95% CI, −3 to 1) at 12 weeks and −1 (95% CI, −2 to 2) at 26 weeks. For the MMSE, the difference in median change between LM11A-31 and placebo was 1 (95% CI, −1 to 2) at 26 weeks.

Next, we performed Fisher’s exact tests to determine whether treatment (placebo or LM11A-31) was associated with clinical function ratings, as measured by the CGI. There were no significant differences in the frequencies of group membership for LM11A-31 compared to placebo at 12 weeks (*P* = 1.00) or the final visit (*P* = 0.836) on the CGI (Extended Data Table 3).

Amunet was used to probe spatial memory. No significant effects of treatment were observed on Amunet scores (*P* > 0.10 for all four Amunet memory subdomains; Supplementary Fig. 1).

**Extended Data Fig. 6:**
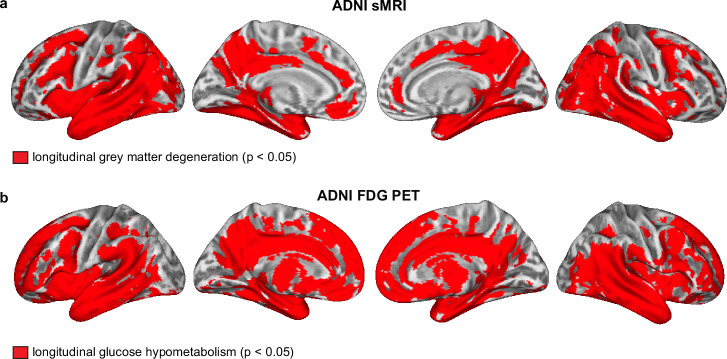
Brain regions vulnerable to AD pathology in the ADNI cohort. Vulnerable brain regions were defined using sMRI and [^18^F]-FDG PET data from an independent sample of participants from the ADNI cohort (n = 54) who met key trial inclusion criteria (see Methods). **a)** Longitudinal patterns of grey matter degeneration were assessed in ADNI over a mean interval of 1 ± 0.2 years. Voxels showing a significant (FWER corrected *P* < 0.05) longitudinal reduction in grey matter volume based on a paired-sample one-sided voxel-wise t test are shown in red. Voxels in red were used as an explicit mask for sMRI analyses of clinical trial data. **b)** Longitudinal reductions in glucose metabolism in the ADNI population over a mean interval of 2.39±   1.96 years. Voxels showing longitudinal reductions in [^18^F]-FDG PET SUVr based on a paired-sample one-sided voxel-wise t-test are shown in red (FWER corrected *P*< 0.05). Voxels shown in red were used as a mask for statistical comparisons of the clinical trial PET data. For both **a)** and **b**), the medial and lateral views shown on the left half of the figure correspond to the left hemisphere of the brain. FWER, family-wise error rate.

We examined whether treatment with LM11A-31 slows longitudinal changes in gray matter integrity, as measured by sMRI, or glucose metabolism, as measured by [^18^F]-FDG PET. To define AD-vulnerable brain regions in an independent cohort, we selected participants from the AD Neuroimaging Initiative (ADNI) with longitudinal sMRI and [^18^F]-FDG PET who met the trial inclusion criteria for age, MMSE score and CSF Aβ42 abnormality (Methods). For each imaging modality, we defined a mask of AD-vulnerable brain regions that exhibited significant longitudinal decreases in gray matter volume or glucose metabolism in the ADNI cohort (Extended Data Fig. 6). We then conducted voxel-wise analyses of variance (ANOVAs) of treatment group (placebo or LM11A-31) by time (baseline or 26-weeks) for the trial sMRI and [^18^F]-FDG PET data, constrained by the corresponding AD-vulnerability masks.

**Fig. 4:**
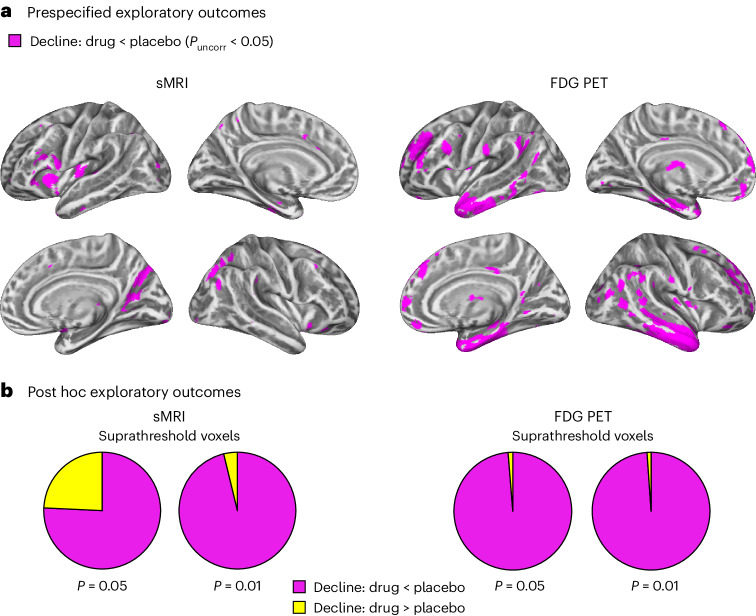
Longitudinal changes in gray matter volume and glucose metabolism in AD-vulnerable brain regions. **a**, Factorial mixed-effects analyses of covariance models examined the two-way interactions between treatment (drug or placebo) and time (before or after treatment). A one-sided *t*-contrast examining the hypothesis-consistent interaction (drug slowing progression over time relative to placebo) revealed that treatment with LM11A-31 slowed longitudinal degeneration (left panels) and glucose hypometabolism (right panels) in the drug group (sMRI, *n* = 127; PET, *n* = 121) compared to the placebo group (sMRI, *n* = 66; PET, *n* = 62). Voxels exhibiting this interaction effect are shown at an uncorrected *P* < 0.05 threshold (magenta) on a population-specific cortical surface. Left and right hemispheres are in the top and bottom rows, respectively. Brain areas exhibiting hypothesis-inconsistent interaction effects are displayed in Extended Data Fig. 7. **b**, The total number of voxels in the a priori AD vulnerability brain areas (total area of pie charts) exhibiting either a hypothesis-consistent (magenta) or a hypothesis-inconsistent (yellow) interaction in each imaging modality (sMRI, left panel; FDG PET, right panel) at increasingly liberal thresholds of uncorrected *P* < 0.01 and *P* < 0.05. Monte Carlo simulations determined that the ratios of voxels exhibiting hypothesis-consistent versus hypothesis-inconsistent effects were significantly higher than those observed on the basis of randomly simulated data for both sMRI and PET (*P* < 0.001 for each; two-sided).

For the voxel-wise sMRI analysis of gray matter volume, a significant hypothesis-consistent treatment group-by-time interaction effect was detected at an uncorrected threshold of *P* < 0.001. Compared to placebo, LM11A-31 slowed rates of gray matter loss in the frontal operculum and posterior parietal cortex. For visualization purposes, these clusters are projected at a more liberal uncorrected threshold of *P* < 0.05 in Fig. 4a (left panels). There were no hypothesis-inconsistent voxels detected at the *P* < 0.001 threshold. For the [^18^F]-FDG PET analysis of brain glucose metabolism, no voxels exhibited a treatment group-by-time interaction effect at the uncorrected threshold of *P* < 0.001. At a more liberal threshold of *P* < 0.05, a hypothesis-consistent treatment group-by-time interaction was detected, where administration of LM11A-31 slowed rates of glucose metabolic decline in regions such as the entorhinal cortex and surrounding temporal cortex, hippocampus, insula and prefrontal cortex (Fig. 4a, right panels).

### Post hoc analyses

**Extended Data Fig. 7:**
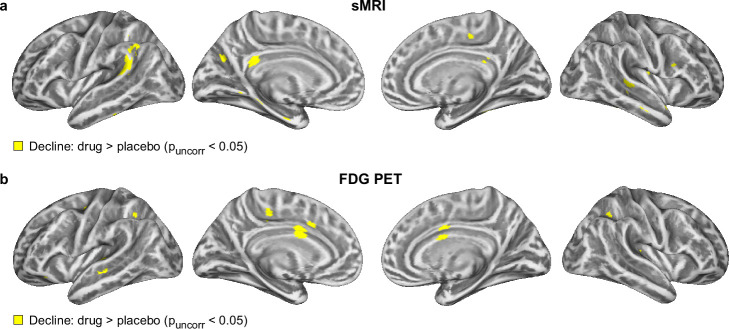
Hypothesis-inconsistent interactions in trial sMRI and [^18^F]-FDG PET Data. Drug group-by-time interactions from voxel-wise factorial mixed ANCOVA models assessing where decline in gray matter volumes (**a**) and glucose metabolism (**b**) is greater in the drug group (sMRI n = 127; PET n = 121) compared to the placebo group (sMRI n = 66; PET n = 62). Voxels exhibiting this effect are shown in yellow (post hoc t contrast; one-sided). Given the exploratory nature of the trial, all *P* values are uncorrected. Hypothesis-consistent interactions (that is, where drug treatment slowed progression of pathology) in sMRI and PET data are shown in Fig. 4. For both **a)** and **b**), the medial and lateral views shown on the left half of the figure correspond to the left hemisphere of the brain.

Although hypothesis-consistent treatment group-by-time interactions were observed in voxel-based exploratory endpoint analyses of both sMRI and [^18^F]-FDG PET data, these findings do not preclude the possibility that a significant majority of subthreshold voxels might exhibit a hypothesis-inconsistent pattern. To test for this possibility, the voxels exhibiting either hypothesis-consistent (LM11A-31 slowing disease progression; Fig. 4a) or hypothesis-inconsistent (LM11A-31 promoting disease progression; Extended Data Fig. 7) treatment group-by-time interactions were counted and expressed as ratios^59^ at increasingly liberal thresholds (*P* < 0.01 and *P* < 0.05, uncorrected) for the sMRI and PET data. The ratios (Fig. 4b) favored the hypothesis-consistent treatment group-by-time interactions in the sMRI (3.1-fold at *P* < 0.05 and 25.5-fold at *P* < 0.01) and PET (76.91-fold at *P* < 0.05 and 89.95-fold at *P* < 0.01) data. Monte Carlo simulations^59^ were then used to test whether the observed majority count ratios of hypothesis-consistent versus hypothesis-inconsistent voxels were significantly different from chance (50:50) at each threshold in the sMRI and [^18^F]-FDG PET datasets. At both thresholds, the ratio of hypothesis-consistent to hypothesis-inconsistent voxels significantly favored a hypothesis-consistent majority (*P* < 0.001 for all, based on 1,000 simulations).

## Discussion

We conducted a phase 2a double-blinded, randomized, placebo-controlled safety and exploratory endpoint trial evaluating the novel therapeutic strategy of targeting p75^NTR^ in a human disease. The trial met its primary endpoint and established the safety of LM11A-31. Safety data revealed that twice-daily oral administration of 200 mg or 400 mg LM11A-31 did not produce safety concerns that would prevent its advancement as a potential AD therapeutic. The most commonly reported AEs in the trial were relatively mild and often transient, including nasopharyngitis, diarrhea and headache, with a small number of participants exhibiting transient, asymptomatic eosinophilia (*n* = 5 in each LM11A-31 dose group). The study population in this trial is reflective of white participants from five European countries. Therefore, it will be important to enroll participants from diverse backgrounds in future trials.

Two of the secondary outcomes (Aβ42 and Aβ40) demonstrated significant drug–placebo differences, although the ratio of Aβ42 to Aβ40 was not affected. No other secondary biomarker or cognitive outcome was statistically significantly different between drug and placebo. Findings from prespecified exploratory outcome measures were consistent with LM11A-31 slowing progression of AD on three biomarker domains (CSF, sMRI and [^18^F]-FDG PET).

Given the novelty of the therapeutic mechanism and the broad signaling effects of p75^NTR^ detected in preclinical work^13,60^, multiple additional biomarkers, particularly those relevant to synaptic integrity and glial status, were included as secondary and exploratory endpoints. Below, connections between the preclinical work and the current study are highlighted for synaptic, glial and core AD biomarkers.

In mouse model studies, LM11A-31 treatment was consistently observed to promote synaptic resilience, as previously reviewed in the literature^11^; LM11A-31 treatment reduced loss of the presynaptic marker synaptophysin in aged mice^61^ and treatment in tau-P301S mice rescued the loss of synaptophysin and the postsynaptic protein PSD95 (ref. ^62^). In the current trial, two CSF presynaptic biomarkers (SNAP25 and SYT1) and one postsynaptic biomarker (NG) were included to examine the effects of p75^NTR^ modulation on synaptic integrity in human AD. Consistent with preclinical findings, LM11A-31 significantly reduced the levels of SNAP25 and NG compared to placebo over the 26-week treatment period (Fig. 2b).

p75^NTR^ is expressed on astrocytes, microglia and oligodendrocytes^18,63^, providing an opportunity for p75^NTR^ modulation to additionally impact non-neuronal cell types affected in AD. In APP^L/S^ mouse models, LM11A-31 reduced histological and PET imaging markers of microglial and astrocyte activation^44,51^. In line with these findings, levels of the glial marker YKL40 were decreased in the LM11A-31 versus placebo group in the present study (Fig. 2b). Further human trials may benefit from characterizing the effects of LM11A-31 by incorporating additional markers of glial status.

Prior preclinical work also implicated an effect of p75^NTR^ modulation with LM11A-31 on tau pathology^43,44,62^. In this trial, LM11A-31 trended toward lowering CSF t-tau compared to placebo (*P* = 0.068), a marker for axonal or neuronal degeneration^64^. Effects of LM11A-31 on p-tau181 and NfL, a marker for axonal degeneration^65^, were not observed in this trial.

Previous studies of APP^L/S^ mice did not observe any effects of LM11A-31 on brain tissue Aβ levels, although mouse CSF levels were not studied^47^. In the present trial, LM11A-31 lowered CSF Aβ42 and Aβ40 levels longitudinally compared to placebo. However, the ratio of Aβ42 to Aβ40 did not differ between the groups (Fig. 2a), suggesting that the treatment is not associated with a change in underlying Aβ pathology. Modulation of p75^NTR^ may reduce both Aβ42 and Aβ40 production through its interactions with the Aβ-generating enzyme β-secretase 1 (BACE-1)^66,67^. Further studies assessing the potential effects of LM11A-31 on p75^NTR^-mediated APP processing will be required.

Currently, in vivo markers of direct p75^NTR^ engagement in humans are lacking. However, the CSF, sMRI and PET biomarkers included in this trial were prespecified on the basis of preclinical research examining pathways and mechanisms regulated by p75^NTR^ (refs. ^13,43–46,68,69^). These include markers related to APP metabolism and Aβ production, synaptic integrity and glial reactivity. Consistent with target engagement, we observed broad effects of LM11A-31 on CSF Aβ40, Aβ42, SNAP25, NG and YKL40. The effects on synaptic measures were further recapitulated by the [^18^F]-FDG PET and sMRI measures, where LM11A-31 slowed declines in gray matter volume and glucose metabolism. Across multiple domains of biomarkers, the direction of these modulatory effects was consistent with slowing pathological progression. To confirm the validity of the selected biomarker panel in the assessment of potential disease-modifying effects of LM11A-31, we also demonstrated that the interrelationships among these biomarkers at baseline were consistent with prior research (Extended Data Fig. 1).

Compared to single-target AD therapeutics, such as anti-Aβ monoclonal antibodies or AChE inhibitors, LM11A-31 regulates multiple core AD pathways in parallel. LM11A-31 modulates p75^NTR^, a deep biology receptor^9^ that has a fundamental role in the control of synaptic integrity, pruning and function^20,69^, and affects multiple mechanisms important in AD, such as tau phosphorylation^46^, inflammation^70^, mitochondrial function^71,72^ and amyloid production^66,67,73^. Consistent with this fundamental role of p75^NTR^ in a broad range of AD pathological cascades, the present clinical trial demonstrated slowing of longitudinal progression of biomarkers of presynaptic, postsynaptic and neuronal integrity (SNAP25, NG and sMRI), synaptic function ([^18^F]-FDG PET) and glial activation (YKL40). The profile of concomitantly affecting both presynaptic and postsynaptic markers of degeneration is particularly notable. Along with the [^18^F]-FDG PET findings, these findings support the hypothesis of slowing synaptic degeneration and will encourage the application of PET-based synaptic quantification in future trials.

In preclinical studies, LM11A-31 administration was able to both prevent^44,47^ and reverse^45^ neuronal and synaptic deficits associated with aging^61^ and mutant APP expression. The ability of LM11A-31 to promote neuronal resilience and restoration suggests that this therapeutic approach could be applied over a broad range of disease stages from presymptomatic to advanced AD. This is in contrast to anti-Aβ monoclonal antibodies that clear protein aggregates but do not directly promote neuronal integrity, theoretically leading to limited therapeutic benefit for persons at later disease stages^7,8,74,75^. The use of LM11A-31 in combination with anti-Aβ therapies might, therefore, produce additive or synergistic effects on protecting synapses.

By design, this phase 2a safety trial had several limitations for detecting cognitive effects, including a small number of participants and a relatively short 26-week study duration^48^. Although the effect was not statistically significant, 26-week treatment with LM11A-31 produced up to 50% slowing of cognitive decline relative to that observed in the placebo group (Fig. 3). Additionally, many of the biomarkers that exhibited a significant AD-slowing drug effect (NG, SNAP25, YKL40, sMRI and [^18^F]-FDG PET) correlated highly with cognitive function at baseline in the trial cohort (Extended Data Fig. 1) and were linked to longitudinal changes in cognition through larger studies with a longer duration^76–78^. Lastly, improved measures of total pathological burden at screening (for example, tau PET) may improve the stratification of participants based on disease stage^79^, thereby improving sensitivity to treatment effects on cognition. Thus, examining the effects of LM11A-31 administration over a longer period with additional strategic biomarkers may reveal that changes in disease pathophysiology are followed by slowing of cognitive decline.

In conclusion, we conducted a placebo-controlled phase 2a trial of LM11A-31, a first-in-class small-molecule modulator of p75^NTR^. The primary safety outcome was met; LM11A-31 was generally well tolerated in a population with mild to moderate AD. Furthermore, the exploratory findings encourage larger trials of longer treatment duration to address the hypothesis that small-molecule modulation of p75^NTR^ might constitute a disease-modifying therapy in AD.

## Methods

### Trial design

This study was a 26-week phase 2a multicenter, randomized, placebo-controlled, double-blind, parallel-group clinical trial of LM11A-31 (LM11A-31-BHS) in participants with mild to moderate AD (EU Clinical Trials identifier: 2015-005263-16; ClinicalTrials.gov identifier: NCT03069014). The trial was initiated at 21 sites located in five European countries: Austria, the Czech Republic, Germany, Spain and Sweden. The trial was conducted in accordance with the Declaration of Helsinki and Good Clinical Practice of the International Council for Harmonization of Technical Requirements for Pharmaceuticals for Human Use (ICH-GCP). All required study documents were submitted to the ethics committees of the participating countries. Each of the five countries involved in the study had a lead site and the institutional review boards (IRBs) at the lead sites provided ethical approval for the study. The IRBs approving the trial were IRB00002556 (Austria), IRB00002091 (Czech Republic), IRB00007525 (Germany), IRB00004959 (Sweden) and IRB00002590 (Spain). The principal investigators of the lead trial sites were Anne Börjesson-Hanson (Sweden; trial coordinating investigator), Reinhold Schmidt (Austria), Jakub Hort (Czech Republic), Oliver Peters (Germany) and Rafael Blesa González (Spain). Participants were enrolled at 18 sites, with the first participant randomized in May 2017 and the last participant completing treatment in June 2020.

Potential study participants were recruited through participating trial sites. Informed consent was obtained before the screening visit. There was no financial incentive to participate in the trial. Study participants were reimbursed for their travel costs.

### Participant eligibility

Participants were eligible for inclusion in the trial if they met the following criteria:

1. Men and women of nonchildbearing potential with a diagnosis of AD according to the McKhann criteria^80^
2. An age of 50–85 years for Austria, Germany, Spain and Sweden or 50–80 years for the Czech Republic
3. MRI or computed tomography assessment within 6 months before study baseline, corroborating the clinical diagnosis of AD and excluding other potential causes of dementia
4. CSF Aβ42 < 550 ng l^−1^ or ratio of Aβ42 to Aβ40 < 0.89
5. MMSE between 18 and 26 (mild to moderate AD)
6. Absence of major depressive disease (Geriatric Depression Scale score < 5)
7. Modified Hachinski Ischemic Scale score ≤ 4
8. Formal education for eight or more years
9. Previous decline in cognition for more than 6 months (based on patient medical records)
10. A caregiver living in the same household or interacting with the participant for a sufficient amount of time each week to ensure administration of drug
11. Living at home or in a nursing home setting without continuous nursing care
12. General health status acceptable for participation in a 26-week clinical trial
13. Able to swallow capsules
14. Stable pharmacological treatment of any other chronic condition for at least 1 month before screening
15. Stable treatment with AChEIs and/or partial *N*-methyl-d-aspartate (NMDA) receptor antagonists for at least 3 months before baseline visit
16. No regular intake of prohibited medications, such as benzodiazepines, neuroleptics, major sedatives, antiepileptics, centrally active antihypertensive drugs (clonidine, l-methyl DOPA, guanidine, guanfacine, etc.), opioid-containing analgesics and nootropic drugs (except ginkgo biloba)
17. Signed consent of the caregiver or participant

There were five protocol amendments, four of which were related to study status, location and recruitment status. One amendment changed the CSF eligibility criteria. The initial criteria were Aβ42 < 530 ng l^−1^ and either t-tau > 350 ng l^−1^ or p-tau181 > 60 ng l^−1^. These criteria were revised to Aβ42 < 550 ng l^−1^ or a ratio of Aβ42 to Aβ40 < 0.89. Of the 242 participants recruited to the trial, the first 13 were recruited under the initial criteria and, thus, met the revised criteria. The full trial protocol is available as Supplementary Note 1.

### Blinding and randomization

Participants were randomized 1:1:1 into placebo, 200 mg LM11A-31 or 400 mg LM11A-31. The randomization list was developed by Data Magik and was structured to allow for a total of at least 240 participants (80 per group), with treatment center as the only stratification variable. A total of 242 participants were finally randomized and treated in the safety population (Fig. 1). ACE pharmaceuticals packaged, labeled and distributed medication packets for the study. Medication kits received by participants were labeled with only individual identification numbers and randomization numbers. The sponsor’s personnel, study sites’ personnel, participants and caregivers were blinded to the assigned treatment.

Participants self-administered medication twice daily (morning and evening) as an oral capsule. A single administration of medication consisted of the following: two capsules of 200 mg placebo (placebo group), one capsule of 200 mg LM11A-31 and one capsule of 200 mg placebo (200 mg LM11A-31 group) or two capsules of 200 mg LM11A-31 (400 mg LM11A-31 group). Placebo capsules contained microcrystalline cellulose with magnesium stearate. All study capsules (LM11A-31 and placebo) were identical in terms of size, color and weight to maintain blinding.

### Determination of sample size

Before the study began, sample size was determined using power calculations that assumed a pooled s.d. of 10 and a two-sided 95% confidence margin. These analyses determined that 51 participants per group were required to demonstrate an effect size of 0.56 between either dose of LM11A-31 and placebo with 80% power and a type 1 error rate of 0.05 (two-tailed), resulting in an initial target of 60 participants per arm for a total of 180 participants. A blinded review of the NTB *z*-score cognitive data from an initial 81 enrolled participants was performed to assess overall pooled variability across the treatment groups. The pooled variability of the NTB was higher than expected and the sample size target was, therefore, increased to 80 participants per arm (240 participants). This was the maximum possible number of participants allowable based on the amount of study medication available.

### Outcome measures

The primary trial outcome was safety (number of AEs or SAEs within the 26-week study period), assessed through AE reporting and participant physical evaluations, including vital signs, blood pressure, 12-lead electrocardiogram, MRI, hematology, blood biochemistry and urinalysis. Clinical safety evaluations were performed using the Columbia Suicide Severity Rating Scale. Secondary biomarker and clinical data were collected and preselected exploratory, longitudinal biomarker and clinical endpoints were also collected. Secondary CSF biomarkers included CSF Aβ40, Aβ42, p-tau181, t-tau and AChE activity. Secondary cognitive outcomes included a composite *z*-score of a custom NTB consisting of a digit span task, a digit symbol substitution task, a category fluency task and a controlled oral word association test (COWAT). Prespecified exploratory biomarker outcomes consisted of the following: the synaptic proteins SNAP25, SYT1 and NG, the microglial protein sTREM2, the astrocytic biomarker YKL40 and the neurodegenerative biomarker NfL. Prespecified exploratory imaging studies included sMRI and [^18^F]-FDG PET. Prespecified exploratory clinical assessments included the MMSE, the ADAS-Cog-13, the Amunet spatial orientation and learning task and the CGI. The CGI provides a measure of clinical function rated by participants’ caregivers as one of seven categorical outcomes (1–3, improvement; 4, no change; 5–7, worsening). A schematic summary of the time points at which the main measures were obtained is provided in Extended Data Table 2.

### CSF biomarker measurements

CSF was collected by lumbar puncture at the screening and final visit (Extended Data Table 2). The samples were analyzed for the core AD CSF biomarkers Aβ42, Aβ40, t-tau and p-tau181 using the Lumipulse technology^81^, on a G1200 instrument. The following kits (name, cat. no.) were used: Lumipulse G β-Amyloid 1–42, 230336; Lumipulse G β-Amyloid 1–40, 231524; Lumipulse G Total Tau Ag, 30312; Lumipulse G pTau 181, 230350 (Fujirebio). The presynaptic proteins SNAP25 and SYT1 were measured using immunoprecipitation followed by mass spectrometry (IP–MS), as described previously in detail^82,83^, using the SMI-81 mouse monoclonal antibody to SNAP25 (Nordic Biosite) for immunoprecipitation that recognizes SNAP25 that is N-terminally acetylated at amino acids 2–11 and anti-CD44 mouse monoclonal antibody clone 41.1 (Synaptic Systems) recognizing the first calcium-binding domain at the N terminus of SYT1. CSF levels of the postsynaptic protein NG were measured using an in-house ELISA method^84^, in which the anti-Ng36 mouse monoclonal antibody (epitope Ng63–75) at a final concentration of 0.5 μg ml^−1^ (100 μl per well) was used as a capturing antibody, while biotinylated anti-Ng2 mouse monoclonal antibody (epitope Ng52–63) at a final concentration of 0.5 μg ml^−1^ (100 μl per well) was used as a detection antibody^85^. CSF sTREM2 concentrations were measured using an in-house Meso Scale Discovery (MSD) immunoassay with streptavidin-coated plates (cat. no. L45SA, MSD), biotinylated goat polyclonal IgG (cat. no. BAF1828, R&D Systems) as a capturing antibody, a mouse monoclonal IgG (cat. no. sc-373828, Santa Cruz Biotechnology) as a secondary antibody and a SULFO-TAG-labeled goat polyclonal anti-mouse antibody (cat. no. R32AC, MSD) for detection, as previously described^86,87^. The CSF level of YKL40 was measured using a commercially available assay (cat. no. DC3L10, R&D Systems) according to the manufacturer’s instructions, using a dilution factor of 1:100 for the samples. CSF NfL was measured using an in-house ELISA method^88^ with the anti-NfL21 (final concentration of 0.5 μg ml^−1^, 100 μl per well) and anti-NfL23 (final concentration of 0.5 μg ml^−1^, 100 μl per well) mouse monoclonal antibodies, both having the core domain of human NfL as the epitope. CSF AChE activity was quantified using an in-house enzymatic Ellman assay^89^, as also described elsewhere in detail^90^. All CSF analyses were performed by board-certified laboratory technicians using methods validated for clinical trials. Baseline and end-of-study CSF samples were analyzed side by side to reduce possible variability. All analyses were performed blinded to the clinical information (see Supplementary Table 2 for further details on CSF antibodies).

### Quantification of longitudinal changes in neuroimaging data

*T*_1_-weighted three-dimensional sMRI scans were obtained before and at the end of treatment (Extended Data Table 2). Longitudinal changes in gray matter volumes were computed in MATLAB, as described previously^91^. Major steps in the sMRI pipeline were longitudinal registration of scans using the serial longitudinal pipeline^92^ in Statistical Parametric Mapping Software version 7771 (https://www.fil.ion.ucl.ac.uk/spm/software/spm12/), segmentation of brain tissue classes in the Computational Anatomy Toolbox (CAT12; version 12.8.1)^93^ and spatial normalization to a population template that was created using the clinical trial sMRI data^94^. Only participants with longitudinal sMRI data (*n* = 206) were analyzed as part of the exploratory outcome analysis.

[^18^F]-FDG PET scans were acquired on the same day as the sMRI scans. Spatial normalization of [^18^F]-FDG PET images relied on deformation fields defined by the sMRI data; therefore, only participants with longitudinal sMRI and PET data (*n* = 197) were analyzed as part of the [^18^F]-FDG PET exploratory outcome analyses. Static-period [^18^F]-FDG PET images were warped to the trial population template and normalized to the mean uptake within a previously defined AD-spared region of interest to produce standardized uptake value ratio (SUVr) images^95^. Detailed quality control information for sMRI and [^18^F]-FDG PET data is available in Supplementary Note 2. The code to perform longitudinal sMRI and PET preprocessing is available on GitHub (https://github.com/hayleyshanks/Longitudinal-MRI-PET-preproc)^96^.

### Quantification of relative changes in CSF biomarkers

Longitudinal changes in CSF biomarkers were quantified using the annual percent change formula^97^ (below) to control for differences in time interval between measurements, while also allowing for the investigation of relative change over time for each participant^97,98^.

$$\textrm{Annual percent change}=\left[{\left(\frac{{\textrm{Final}}}{{\textrm{Screening}}}\right)}^{\frac{365}{{\textrm{Time difference}}\left({\textrm{days}}\right)}}-1\right]{\rm{\times}}100$$

### Definition of AD-vulnerable brain regions

Data from the ADNI (https://adni.loni.usc.edu/)^99^ were used to identify brain regions vulnerable to reductions in gray matter volume and glucose metabolism in an independent AD cohort. The ADNI was launched in 2003 as a public–private partnership, clinical trial-like natural history study, led by principal investigator Michael W. Weiner. The primary goal of ADNI is to test whether serial MRI, PET and other biological markers, as well as clinical and neuropsychological assessment, can be combined to measure the progression of mild cognitive impairment and early AD (for up-to-date information, see www.adni-info.org).

Participants from ADNI-GO-2 or ADNI-3 were included if they met the trial inclusion criteria for age (50–85 years old), MMSE score (18–26) and CSF Aβ abnormality. In the ADNI, CSF Aβ abnormality is defined as <880 pg ml^−1^ (ref. ^100^). ADNI participants were required to have two time points of 3-T structural MRI data (mean interval: 1 year) and [^18^F]-FDG PET data (mean interval: 2.39 years). The final ADNI sample consisted of 54 participants. At baseline, the ADNI sample had a median MMSE score of 24.12 and a median age of 74.5 years. The sample was 57.4% male and 77.8% of participants were carriers of at least one *APOE4* allele.

sMRI and PET preprocessing of ADNI data was performed as described above. Voxel-wise paired-sample *t*-tests in CAT12 (ref. ^93^) assessed reductions in gray matter volume and reductions in glucose metabolism in the ADNI cohort (Extended Data Fig. 6). Analyses were restricted to regions within the clinical trial population template gray matter segment that had a probability of at least 0.1 of belonging to the gray matter.

### Statistical analyses

Statistical analyses were performed using SAS version 9.2 or above, MATLAB 2021b and R version 4.2.2. Statistics for primary trial outcomes were performed in a two-tailed manner with a significance threshold of *P* < 0.05. Statistical analyses of the primary trial outcome (safety) consisted of calculating ORs with 95% CIs for each LM11A-31 dose group relative to placebo. All participants in the safety population were included in these calculations (*n* = 242).

For statistical analyses of exploratory endpoints, participants from the ITT population (*n* = 241) who had longitudinal data for that specific outcome that passed quality control were included (Supplementary Information).

Brain exposure estimations based on pharmacokinetic studies of CSF samples from normal human participants, along with normal and AD model mice administered LM11A-31, suggested that the twice-daily doses of both 200 mg and 400 mg were sufficient for full engagement of targeted mechanisms. In preliminary analyses of dose-specific responses of the exploratory endpoint data, outcome measures were similar across both doses. As a result, data from both doses of drug were pooled for exploratory endpoint analyses. Analyses for each dose group are available in Extended Data Figs. 2–4. Between-dose-group differences of secondary and exploratory endpoints were assessed using Kruskal–Wallis tests. Significant Kruskal–Wallis tests were followed with post hoc Dunn’s tests.

Preplanned analyses were separately performed at the trial outset on each of the primary safety, secondary and prespecified exploratory endpoints (CSF, MRI, [^18^F]-FDG PET and cognitive test data) before aggregation of participant metadata. These metadata included blinded data quality analysis, calculation of data covariates (for example, intracranial volume) and variability in treatment period across participants, as a result of early discontinuations and/or limitations in clinic site access (mean treatment period: 214 ± 39 days). The preplanned analyses did not detect significant differences between drug and placebo in baseline versus post-treatment levels. Follow-up analyses incorporating these metadata were performed by two independent research labs (T.W.S. and E.M.R.) and are discussed in the remainder of this section.

Relative changes in CSF biomarkers were quantified using annual percent change, which accounts for variability in the treatment period and baseline concentration across participants. Relative changes in cognitive test scores were quantified by subtracting each participant’s baseline or screening score from their final test score (change = final score − initial score). For the ADAS-Cog-13 and the NTB *z*-score, the change was additionally quantified at the 12-week visit in this manner (change = 12-week score − initial score; Extended Data Fig. 5). For CSF and cognitive data, outliers were defined as any data point that exceeded three scaled median absolute deviations from the median and were removed before statistical analyses. The exclusion of outliers did not alter the significance of statistical tests at a threshold of *P* < 0.05. Wilcoxon rank sum tests compared whether the longitudinal change differed between the placebo and the LM11A-31 groups. The 95% CIs for differences between the medians of the LM11A-31 and placebo groups were calculated using bootstrapping with 5,000 iterations, as previously described^101^. Nonparametric tests were chosen because change data were not normally distributed according to the Kolmogorov–Smirnov test.

For the MRI and [^18^F]-FDG analyses, we conducted exploratory voxel-wise analyses within an independently defined mask of brain regions vulnerable to AD neurodegeneration. AD-masking approaches were previously applied in the analysis of neuroimaging data in AD clinical trials^102^. Statistical analysis of sMRI and [^18^F]-FDG PET data was performed in CAT12 using voxel-wise flexible factorial models^93^. Models included the drug group (placebo or LM11A-31) and time (baseline or follow-up) as factors. Additionally, we included a factor controlling for participant-specific variables that do not change over time (including sex, *APOE* genotype and trial site or scanner)^93^. Flexible factorial models were restricted to the gray matter regions identified to be vulnerable to AD in the ADNI sample (Extended Data Fig. 6). Drug group-by-time analyses presented in Fig. 4 and Extended Data Fig. 7 are shown at a threshold of *P* < 0.05 (uncorrected, one-tailed).

For analysis of the Amunet data, we performed a 2 × 2 repeated-measures ANOVA with treatment (placebo or LM11A-31) as the between-participant factor and time (baseline or week 26) as the repeated-measures factor for each of the four spatial memory domains probed by Amunet (allocentric, egocentric, allocentric + egocentric and allocentric delayed). The dependent measure was the total error on each memory domain. ANOVAs were performed in a two-tailed manner.

Spearman correlations were used to assess relationships between cognitive and biomarker measures at baseline. Given that these measurements were performed before treatment, Spearman correlations were conducted on all participants regardless of treatment group.

CGI data collected at 12 weeks and the final visit (26-week visit or early discontinuation) were analyzed using Fisher’s exact tests. These tests assessed relationships between treatment group and CGI categorical membership at each time point.

Consistent with an exploratory trial format^48^, statistical analyses presented in this paper were not corrected for multiple comparisons.

### Reporting summary

Further information on research design is available in the Nature Portfolio Reporting Summary linked to this article.

## Online content

Any methods, additional references, Nature Portfolio reporting summaries, source data, extended data, supplementary information, acknowledgements, peer review information; details of author contributions and competing interests; and statements of data and code availability are available at 10.1038/s41591-024-02977-w.

### Supplementary information

Supplementary Information Supplementary Tables 1 and 2, Fig. 1 and Notes 1 and 2.

Reporting Summary

## Figures and Tables

**Extended Data Table 1:**
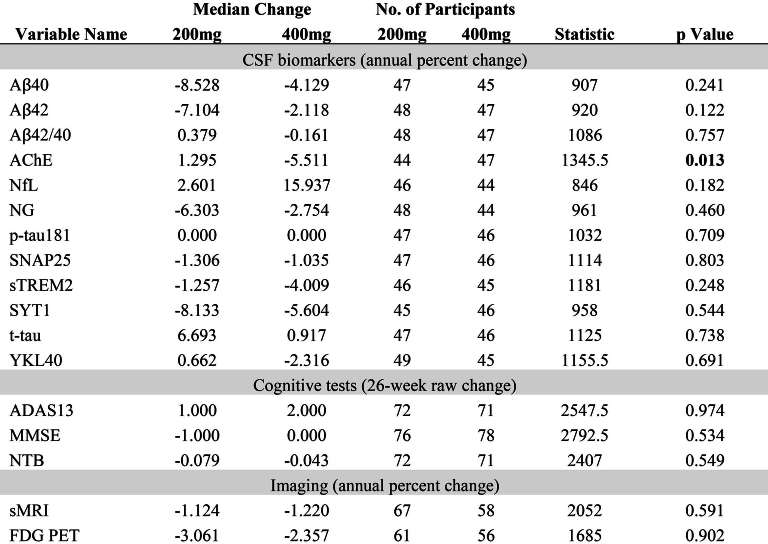
Comparison of median 26-week change between 200 mg and 400 mg LM11A-31 groups

Groups were compared using Wilcoxon rank sum tests (two-sided, uncorrected for multiple comparisons). Data used to derive statistics in this table can be visualized in Extended Data Figs. 2–4. Neuroimaging analyses were restricted to AD-vulnerable brain regions, as defined in an independent AD cohort (Extended Data Fig. 6).

**Extended Data Table 2:**
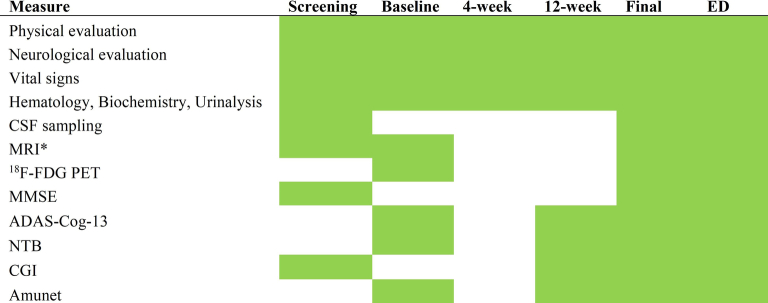
Collection schedule of key trial outcome measures

*MRI was performed at screening if no MRI scan was available within 6 months prior to baseline visit. All patients needed to have an MRI according to the MRI protocol at baseline if no MRI was performed at the screening visit. ED = early discontinuation visit.

**Extended Data Table 3:**
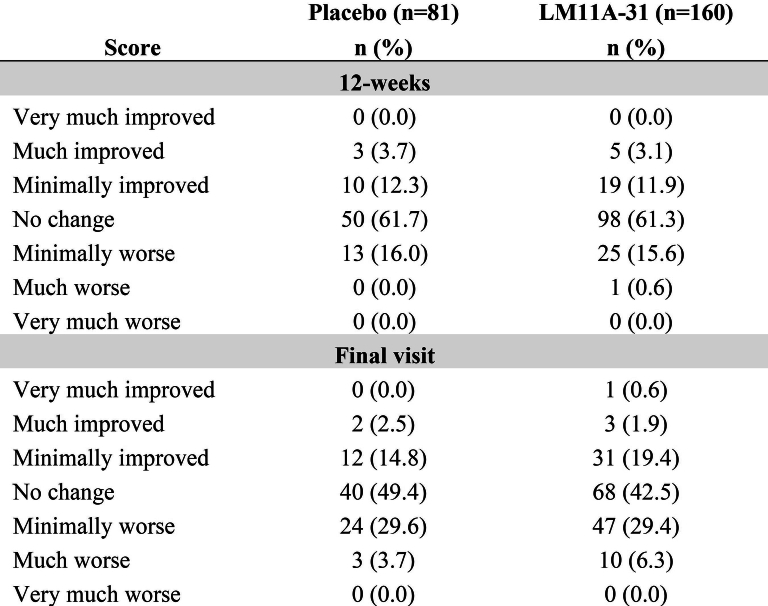
Clinical global impression scale-improvement under placebo and LM11A-31

CGI-Improvement scores at the 12-week and final visit for patients in the intention to treat population. The proportion of participants in each category did not differ by treatment group at the 12-week (p = 1.00) or final timepoint (*p* = 0.836) according to a Fisher’s Exact test. The n refers to the total subjects that were assessed as part of the ITT population, and all percentages are based on the total n.

## Data Availability

Data files containing pseudonymized participant data (baseline characteristics, raw data used to conduct primary and exploratory endpoint analyses reported in this article) can be shared in compliance with current data protection regulations by the EU. All requests for data access should be directed to the corresponding authors. Requests for data will be reviewed and responded to within a 1-month period. Data can be shared through data use agreements for research or academic purposes only.

## Code availability

The custom code to perform the majority count statistics and Monte Carlo simulations (Fig. 4b) developed by K.C. is not publicly available but may be made available to qualified researchers on reasonable request to the corresponding authors. Wrapper scripts to call SPM12 and CAT12 functions for MRI and PET preprocessing are available at https://github.com/hayleyshanks/Longitudinal-MRI-PET-preproc (ref. ^96^).
