# Supplementary material for: p75 neurotrophin receptor modulation in mild to moderate Alzheimer disease: a randomized, placebo-controlled phase 2a trial
Source: Nat Med. 2024 May 17;30(6):1761–70. doi: 10.1038/s41591-024-02977-w (PMC11186782; doi:10.1038/s41591-024-02977-w)
Supplement: Supplementary file 1 — Supplementary Tables 1 and 2, Fig. 1 and Notes 1 and 2. [file 41591_2024_2977_MOESM1_ESM.pdf]

nature medicine

Article

https://doi.org/10.1038/s41591-024-02977-w

p75 neurotrophin receptor modulation in mild to moderate Alzheimer disease: a randomized, placebo-controlled phase 2a trial

In the format provided by the authors and unedited

p75 neurotrophin receptor modulation in mild to moderate Alzheimer's disease: A randomized, placebo-controlled phase 2a trial

List of supplementary information:

Sections of the supplementary information are hyperlinked below:

- Supplementary Table 1: Site Principal Investigators list
- Supplementary Table 2: Extended CSF antibody information
- Supplementary Figure 1: Amunet spatial memory testing
- Supplementary Note 1: Clinical trial protocol
- Supplementary Note 2: Extended imaging methods

Supplementary Table 1 | Trial site Principal Investigators and current affiliations

Site Principle Investigator	Affiliation	Country
**Anne Börjesson-Hanson**	Clinical Trials,<br>Department of Aging, Karolinska University Hospital	Sweden
**Jakub Hort**	Memory Clinic, Department of Neurology, Charles University, Second Faculty of Medicine and Motol University Hospital	Czech Republic
**Ladislav Pazdera**	Vestra Clinics	Czech Republic
**Katerina Sheardova**	Neurology Clinic of Martin Urbanek	Czech Republic
**Martin Valis**	NEUROHK, s.r.o.	Czech Republic
**Daniel Bittner**	Universitätsklinikum Magdeburg	Germany
**Ralf Bodenschatz**	Pharmakologisches Studienzentrum Chemnitz GmbH	Germany
**Andrej Pauls**	Neurologie Sendlinger-Straße<br>Studien- und Gedächtniszentrum	Germany
**Robert Perneczky**	Department of Psychiatry and Psychotherapy, LMU Hospital, Ludwig-Maximilians-Universität München, Munich	Germany
**Oliver Peters**	Charité Universitätsmedizin Berlin<br>Klinik für Psychiatrie und Psychotherapie	Germany
**Irma Schöll**	Zentrum für klinische Forschung	Germany
**Joachim Springub**	Fachübergreifende Gemeinschaftspraxis für Neurologie und Psychiatrie/ Psychotherapie	Germany
**Rafael Blesa González**	Fundación de Gestión Sanitaria del Hospital de la Santa Creu I Sant Pau	Spain
**Mercè Boada Rovira**	Ace Alzheimer Center Barcelona – Universitat Internacional de Catalunya<br><br>Networking Research Center on Neurodegenerative Diseases (CIBERNED), Instituto de Salud Carlos III, Madrid	Spain
**Emilio Franco Macías**	FISEVI<br>Hospital Universitario Virgen del Rocío	Spain
**Raquel Sánchez-Valle**	Alzheimer's disease and other cognitive disorders Unit<br><br>Hospital Clínic de Barcelona, FRCB-IDIBAPS, Barcelona	Spain
**Douglas Imarhiagbe**	LKH Hall, Abteilung für Psychiatrie A, Gerontopsychiatrische Ambulanz mit Gedächtnissprechstunde<br>Milser Straße 10<br>6060 Hall in Tirol	Austria
**Reinhold Schmidt**	Medizinische Universität Graz, Universitätsklinik für Neurologie	Austria

Supplementary Table 2 | Extended CSF antibody information.

	YKL-40	Aβ40	Aβ42	p-tau181	t-tau	sTREM2	AChE	NFL	Neurogranin
**Cat. No.**	K151NHD					L45SA	In house	In house	In house
**Kit lot**	P239035 (384)<br>P257601 (8)								
**Cartridge lot**		4YX0115E	7ZX0121E	5DX0122E	6BX0122E				
**Control lot**		3C0121E	3C0121E	5H1022E	5G0122E				
**Calibrator lot**		4ZX1032E (382)<br>4ZX1063E (10)	5JX1021E (382)<br>5JX1072E (10)	5H0111E (382)<br>5EX1022E (10)	6CX0121E (382)<br>6CX1042E (10)				
**Substrate lot**		AJX1022E	AJX1022E	AJX1022E	AJX1022E				
**Capture Ab lot**						KICO618081			
**Standard lot**						LCL12JL2401			
**Detection Ab lot**						I2917			
**Plate lot**						Z0021461			
**SULFO-ab lot**						WOO184427s			
**Read solution**						Y0140364			
**Calibrator lot**							1,2U 190524		
**ATCH Substrate lot**							160523		
**DTNB Substrate lot**							171102		
**Coating antibody lot**								07/FEB/2017 LB116-17<br>Coated 06-07/apr/2020 (228)<br>and 19-20/nov/2020 (164)	180525 LB155_48<br>Coated 09-10/may/2019
**Biotinylated antibody lot**								Lot okänd<br>Biotinylated 12/oct/2020	LB191_03 181018<br>Biotinylated 11/sep/2020
**Conjugate lot**								25-mar-2020	14-sep-2017
**Substrate lot**								200103	180808

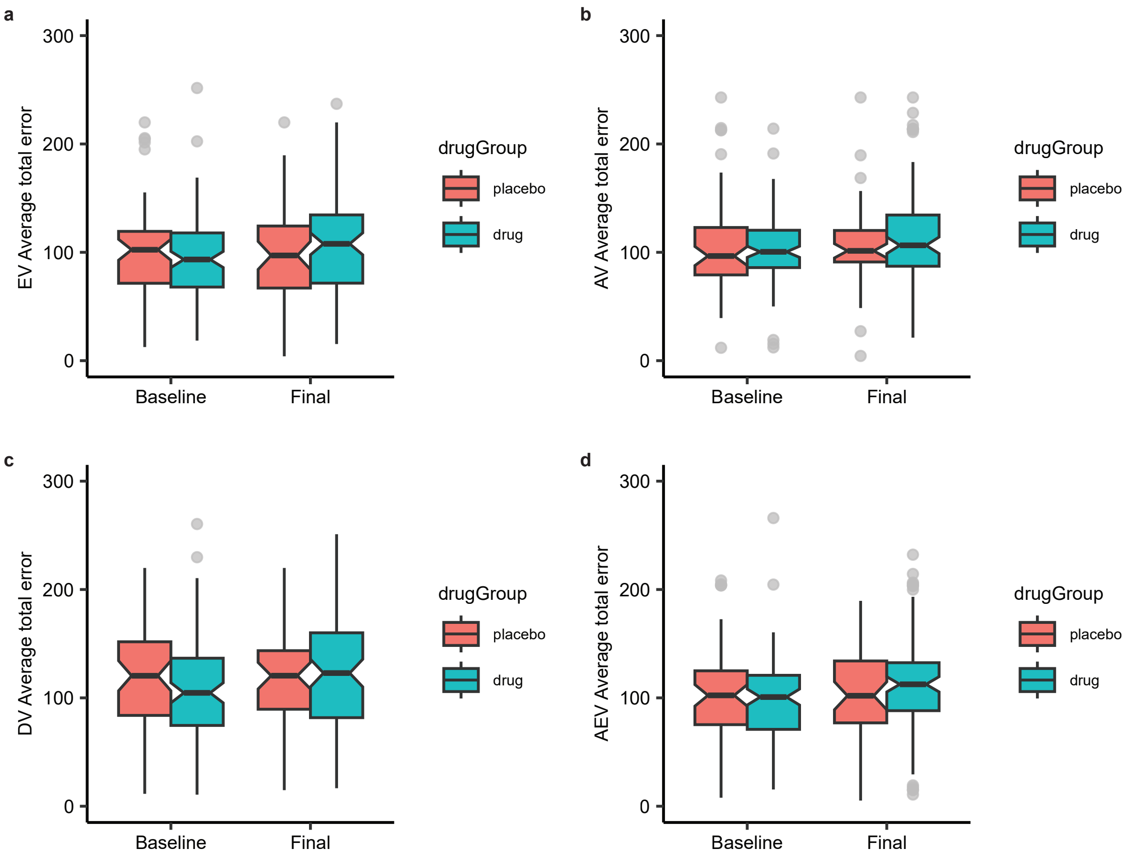

Supplementary Figure 1 | Amunet spatial memory testing. The Amunet is a computerized human version of the Morris Water Maze, designed to probe various components of spatial memory. Boxplots show scores on the four Amunet memory domains at the baseline and final visit for participants in the placebo (salmon) and LM11A-31 (teal) groups. Mixed ANOVA analyses were performed with treatment group (placbeo, LM11A-31) and visit (baseline, final) as factors to assess changes in spatial memory in each group. Significant main effects of visit, or treatment group-by-visit interactions, were not observed in any of the Amunet spatial memory domains. **a**) Egocentric navigation ($n_{placebo}$ = 47; n_{drug} = 88), $p = 0.244$, **b**) allocentric navigation, ($n_{placebo}$ = 47; n_{drug} = 88) $p = 0.146$, **c**) delayed navigation ($n_{placebo}$ = 45; n_{drug} = 87), $p = 0.351$, **d**) allocentric-egocentric navigation ($n_{placebo}$ = 47; n_{drug} = 91), $p = 0.223$. *p* values reflect the significance of the group-by-visit interaction effects, and are not corrected for multiple comparisons. Sample sizes differ due to missing data. Black horizontal lines on box plots represent the median of each distribution. Notches provide 95% confidence intervals of the median, which represent the reliability of within-group change. The lower and upper hinges of the boxplot correspond to the first and third quartiles of the distribution, and the whiskers (vertical lines) extend from the hinge to the largest or smallest value, no further than ±1.5 times the interquartile range from the hinge.

Clinical Trial Protocol
Phase IIa
LM11A-31-BHS
Version 4.1

1 TITLE PAGE

**Study title**	**A 6-months prospective, multi-center, double-blind, placebo-controlled, randomized, adaptive-trial-design study to evaluate safety, tolerability and exploratory endpoints of either placebo or two different oral doses of LM11A-31-BHS in patients with mild to moderate probable Alzheimer's disease**
**Study code**	**NSC15001**
**EudraCT Number**	**2015-005263-16**
**Sponsor**	**PharmatrophiX Inc.**<br>2198 Sterling Avenue<br>Menlo Park<br>California<br>CA 94025<br>US
**Date**	**25 Jul 2019**
**Version**	**Final Version 4.1**

The study will be conducted in compliance with the protocol, ICH GCP Guidelines, the Declaration of Helsinki and any applicable local regulatory requirements.

Once signed, the terms of the protocol are binding for all parties.

CONFIDENTIAL AND PROPRIETARY

The contents of this document are confidential and proprietary to PharmatrophiX Inc. Unauthorized use, disclosure or reproduction is strictly prohibited. It is understood that the information will be disclosed to the Investigator's staff, applicable Independent Ethics Committee(s) / Institutional Review Board(s), Regulatory Authorities, and study participants to obtain informed consent without prior written approval from PharmatrophiX.

2 OVERVIEW

**Study title**	**A 6-months prospective, multi-center, double-blind, placebo-controlled, randomized, adaptive-trial-design study to evaluate safety, tolerability and exploratory endpoints of either placebo or two different oral doses of LM11A-31-BHS in patients with mild to moderate probable Alzheimer's disease**
**Study code**	**NSC15001**
**EudraCT Number**	**2015-005263-16**
**Study phase**	IIa
**Indication**	Alzheimer's disease
**Study period**	June 2016–December 2019
**Individual treatment period**	26-weeks double-blind
**Investigational product**	LM11A-31-BHS
**Active ingredient**	LM11A-31-BHS
**Dose**	200mg bid or 400mg bid of free base
**Dosage form**	Solid, powder-filled hard gelatin capsules for oral administration containing 200 mg LM11A-31-BHS (free base) or placebo
**Responsible for the content of the trial protocol**	Manfred Windisch, Ph.D.,<br>**NeuroScios GmbH**<br>Graz – Austria
**Responsible Biometrician**	David Fleet, M.Sc. C.Stat<br>Data Magik Limited<br>Salisbury UK

3 SIGNATURE PAGES

**Manfred Windisch, PhD**<br>Responsible Scientific Expert<br>NeuroScios GmbH	2019 07 30<br>Date	Signature
**Nikola Helmberg, PhD**<br>Project Manager, NeuroScios GmbH	2019 07 30<br>Date	Signature
**David Fleet, M.Sc C.Stat**<br>Biometrician, Data Magik Ltd	Date	Signature
**Prof. Dr. Agneta Nordberg**<br>Central Reader for PET Scans	Date	Signature
**Prof. Dr. Anne Börjesson Hanson**<br>Coordinating Investigator	Date	Signature
**Anne Chun Longo**<br>CEO, PharmatrophiX Inc.	Date	Signature
**Frank M. Longo, MD, PhD**<br>Chairman of the Board and founder of PharmatrophiX	Date	Signature

3 SIGNATURE PAGES

**Manfred Windisch, PhD**<br>Responsible Scientific Expert<br>NeuroScios GmbH	Date	Signature
**Nikola Helmberg, PhD**<br>Project Manager, NeuroScios GmbH	Date	Signature
**David Fleet, M.Sc C.Stat**<br>Biometrician, Data Magik Ltd	29 July 19<br>Date	Signature
**Prof. Dr. Agneta Nordberg**<br>Central Reader for PET Scans	Date	Signature
**Prof. Dr. Anne Börjesson Hanson**<br>Coordinating Investigator	Date	Signature
**Anne Chun Longo**<br>CEO, PharmatrophiX Inc.	Date	Signature
**Frank M. Longo, MD, PhD**<br>Chairman of the Board and founder of PharmatrophiX	Date	Signature

3 SIGNATURE PAGES

**Manfred Windisch, PhD**<br>Responsible Scientific Expert<br>NeuroScios GmbH	Date	Signature
**Nikola Helmberg, PhD**<br>Project Manager, NeuroScios GmbH	Date	Signature
**David Fleet, M.Sc C.Stat**<br>Biometrician, Data Magik Ltd	Date	Signature
**Prof. Dr. Agneta Nordberg**<br>Central Reader for PET Scans	29.7.19<br>Date	Signature
**Prof. Dr. Anne Börjesson Hanson**<br>Coordinating Investigator	Date	Signature
**Anne Chun Longo**<br>CEO, PharmatrophiX Inc.	Date	Signature
**Frank M. Longo, MD, PhD**<br>Chairman of the Board and founder of PharmatrophiX	Date	Signature

3 SIGNATURE PAGES

**Manfred Windisch, PhD**<br>Responsible Scientific Expert<br>NeuroScios GmbH	Date	Signature
**Nikola Helmberg, PhD**<br>Project Manager, NeuroScios GmbH	Date	Signature
**David Fleet, M.Sc C.Stat**<br>Biometrician, Data Magik Ltd	Date	Signature
**Prof. Dr. Agneta Nordberg**<br>Central Reader for PET Scans	Date	Signature
**Prof. Dr. Anne Börjesson Hanson**<br>Coordinating Investigator	06 AUG 2019<br>Date	Signature
**Anne Chun Longo**<br>CEO, PharmatrophiX Inc.	Date	Signature
**Frank M. Longo, MD, PhD**<br>Chairman of the Board and founder of PharmatrophiX	Date	Signature

3 SIGNATURE PAGES

**Manfred Windisch, PhD**<br>Responsible Scientific Expert<br>NeuroScios GmbH	Date	Signature
**Nikola Helmberg, PhD**<br>Project Manager, NeuroScios GmbH	Date	Signature
**David Fleet, M.Sc C.Stat**<br>Biometrician, Data Magik Ltd	Date	Signature
**Prof. Dr. Agneta Nordberg**<br>Central Reader for PET Scans	Date	Signature
**Prof. Dr. Anne Börjesson Hanson**<br>Coordinating Investigator	Date	Signature
**Anne Chun Longo**<br>CEO, PharmatrophiX Inc.	July 29, 2019<br>Date	[signature]<br>Signature
**Frank M. Longo, MD, PhD**<br>Chairman of the Board and founder of PharmatrophiX	July 29, 2019<br>Date	[signature]<br>Signature

3.1 Coordinating Investigator Signature

Study Title:

A 6-months prospective, multi-center, double-blind, placebo-controlled, randomized, adaptive-trial-design study to evaluate safety, tolerability and exploratory endpoints of either placebo or two different oral doses of LM11A-31-BHS in patients with mild to moderate probable Alzheimer's disease

Trial Number: NSC15001

EudraCT Number: 2015-005263-16

I herewith agree to adhere to the trial protocol and to all documents referenced in the trial protocol.

Name:

Prof. Dr. Anne Börjesson Hanson

Site:

Clinical Drug Research and Intervention Studies

Theme Aging, R&D

Geriatriska kliniken Karolinska

Universitetssjukhuset

Minnesmottagningen, M51

Huddinge

141 86 Stockholm

Date: 06 AUG 2019 Signature: [signature]

3.2 LOCAL SIGNATURE (PRINCIPAL INVESTIGATOR OF SITE)

I have thoroughly read and reviewed the trial protocol.

Having understood the requirements and conditions of this trial protocol, I agree:

- To perform the clinical study according to this protocol, international Good Clinical Practice principles and Regulatory Authority requirements;
- To provide direct access to source data/documents (source document verification);
- To permit study-related monitoring, audits, IEC/IRB review, and Regulatory Authority inspections;
- To use the study material, including medication only as specified in this protocol;
- To adhere the following time schedule: The first patients will be enrolled January 2017 and therapy is foreseen to be completed in July 2018;
- To report to the responsible Study Safety Officer, within one working day, any Adverse Event that is serious, whether considered treatment-related or not;
- To sign this Trial Protocol before the study formally starts.

I understand,

- That any changes to the protocol must be made in the form of an amendment, which has the prior written approval of PharmatrophiX.
- That the content of this protocol is confidential and photocopying is not allowed;
- That any violation of this protocol may lead to early termination of the study site.

____________________	________	____________________
Principal Investigator<br>(Print Name)	Date	Signature

Stamp of investigational site:

4
TABLE OF CONTENT

1 TITLE PAGE 1
2 OVERVIEW 2
3 SIGNATURE PAGES 3
3.1 Coordinating Investigator Signature 4
3.2 LOCAL SIGNATURE (PRINCIPAL INVESTIGATOR OF SITE) 5
4 6
5 SYNOPSIS 10
6 INVESTIGATORS AND STUDY ADMINISTRATIVE STRUCTURE 18
7 LIST OF ABBREVIATIONS AND DEFINITION OF TERMS 20
8 INTRODUCTION 23
8.1 Clinical Experience 24
8.2 Risk/Benefit Assessment 25
8.2.1 Principal hazards of the medicinal product identified in non-clinical studies 25
8.2.2 Suggested mechanisms and exposure thresholds for such untoward effects exposure margins in the suggested trial based on systemic AUC and Cmax rather than dose 26
8.2.3 Possible risks to trial subjects in the proposed study 27
Toxicity Assessment 28
Pharmacokinetic Assessment 29
8.2.4 Principal hazards of the medicinal product identified in clinical studies 29
8.2.5 Measures taken to monitor and manage predicted risks 30
9 RESEARCH STRATEGY 31
9.1 General Considerations 34
9.2 Appropriateness of measurements 34
9.3 Treatment Groups 34
9.4 Choice of study medication dosage 35
9.5 Duration of the treatment 35
9.6 Randomization 35
9.7 Selection of Study Population 36
9.7.1 Inclusion criteria 37
9.7.2 Exclusion criteria 38
9.8 Removal of patients from treatment or assessment 39
9.9 Premature termination or suspension of the study 40
10 STUDY OBJECTIVES 41
10.1 Primary/ Safety Variables 41
10.1.1 Vital Signs 42
10.1.2 Blood pressure (BP - systolic and diastolic) 42

10.1.3 12-Lead ECG 42
10.1.4 MRI 43
10.1.5 Laboratory 43
10.1.6 C-SSRS 43
10.2 Efficacy/ Exploratory Endpoints 43
10.2.1 Regional Brain Glucose Metabolism (^{18}F-FDG-PET) 43
10.2.2 CSF Biomarker 43
10.2.3 Neurological Testing battery (NTB) 44
10.2.4 Alzheimer's Disease assessment scale – 13items (ADAS-Cog 13 items) 44
10.2.5 Spatial Navigation Testing with Amunet 45
10.2.6 Clinical Global Impression Scale 45
10.2.7 Geriatric Depression Scale (GDS) 46
10.3 Pharmacokinetics 46
10.4 Diagnostic Variables 46
10.4.1 Laboratory 46
10.4.2 ApoE genotyping 46
10.4.3 CSF AD specific lab details 47
10.4.4 Mini-Mental State Examination (MMSE) 47
10.4.5 Hachinski Ischemic Scale 47
11 STUDY MEDICATION (LM11A-31-BHS) 48
11.1 Identity of the investigational product(s) 48
11.2 Investigational drug product packaging 48
11.3 Treatments administered 50
11.4 Selection of doses in the study 51
11.5 Selection and timing of dose for each subject 51
11.6 Blinding 51
11.7 Prior and Concomitant Therapy 52
11.8 Prohibited Concomitant Medications 53
11.9 Permitted Concomitant Medications 53
11.10 Treatment Compliance and Drug Accountability 53
11.11 Drug Accountability at the Site 54
12 LABORATORY 55
12.1 Diagnostic Parameters 55
12.2 Safety Blood and Urinalysis 56
12.3 Pharmacokinetics 56
12.4 Lumbar puncture for CSF collection 56
13 STUDY PROCEDURES 58
13.1 Flow Chart 58

13.2 Screening Procedures at Visit 1 59
13.3 Baseline Procedures at Visit 2: 59
13.4 Procedures at Visit 3 (4 Weeks): 60
13.5 Procedures at Visit 4 (12 Weeks): 61
13.6 Procedures at Visit 5 (26 Weeks): 61
13.7 Procedures at Early Discontinuation Visit 62
14 SAFETY REPORTING, PHARMACOVIGILANCE 64
14.1 Adverse Events (AEs) 64
14.2 Documentation 64
14.3 Definition of severity 65
14.4 Definition of causality relationship with investigational product 65
14.5 Categories of actions taken and outcome 66
14.5.1 Action(s) taken to drug: 66
14.5.2 Outcome 66
14.6 Serious Adverse Event (SAE) 66
14.7 Expedited Reporting to Competent Authorities and Ethics Committees 67
14.7.1 Definition of serious and severe 67
14.7.2 Monitoring of Patients with Adverse Events 67
14.7.3 Unexpected Adverse Events 67
14.7.4 Expected Adverse Events 67
15 DATA QUALITY CONTROL AND QUALITY ASSURANCE 68
16 STATISTICAL METHODS AND DETERMINATION OF SAMPLE SIZE 69
16.1 Adaptive Design 69
16.1.1 Interim Data Review 69
16.2 Analysis Populations 70
16.3 Missing Data 70
16.4 Data Analysis 70
16.5 Evaluation of Safety and Tolerability Parameters 71
16.5.1 Adverse events 71
16.5.2 Clinical laboratory test values 71
16.5.3 Vital signs and ECG parameters 71
16.5.4 Physical examination 71
16.5.5 Columbia-Suicide Severity Rating Scale 71
16.5.6 MRI 71
16.6 Exploratory/ Efficacy Analyses 71
16.6.1 Cognitive Tests 72
16.6.2 Other Tests 72
16.6.3 Separate PET Scan Data Analyses 73

16.6.4 Demographic and Baseline Characteristics 73
16.6.5 Diagnostic Criteria 73
16.6.6 Sample Size 73
16.6.7 Sample Size Justification 73
17 ETHICS AND REGULATORY 74
17.1 Independent Ethics Committee (IEC) or Institutional Review Board (IRB) 74
17.2 Regulatory Authority 74
17.3 Ethical conduct of the study 74
17.4 Patient information and consent 74
17.5 Insurance 75
17.6 Data handling and record keeping 75
18 FINANCING 76
19 PUBLICATION POLICY 76
20 APPENDIX 1 - World Medical Association Declaration of Helsinki 77
21 APPENDIX 2 - Elements of Informed Consent 81
22 APPENDIX 3 - Mini Mental State Examination (MMSE) 83
24 APPENDIX 4 -Digit Span Test 86
25 APPENDIX 5 – Category Fluency Test 88
26 APPENDIX 6 - Controlled Oral Word Association Test (COWAT) 89
27 APPENDIX 7 - Digit Symbol Substitution Test 90
28 APPENDIX 8 - Alzheimer’s Disease Assessment Scale - Cognitive Items 91
29 APPENDIX 9 - Geriatric Depression Scale 15-Item - Short Version 98
30 APPENDIX 10 - Spatial Orientation and Learning (AMUNET) 99
31 APPENDIX 11 - Clinical Global Impression – Severity/Improvement 103
32 APPENDIX 12 - The Modified Hachinski Ischemic Scale 104
33 APPENDIX 13 - C-SSRS 105
34 APPENDIX 14 - Administrative Matters 107
34.1 Responsibilities of the Investigator 107
34.2 Protocol Amendments 107
34.3 Sponsor’s Termination of Study 107
34.4 Electronic Case Report Form Instructions 107
34.5 Monitoring 107
34.6 Archiving of Data 108
34.7 Audits 108
35 REFERENCES 109

5 SYNOPSIS

**Study title**	**A 6-months prospective, multi-center, double-blind, placebo-controlled, randomized, adaptive-trial-design study to evaluate safety, tolerability and exploratory endpoints of either placebo or two different oral doses of LM11A-31-BHS in patients with mild to moderate probable Alzheimer's disease**
**Study code**	NSC15001
**EudraCT No.**	2015-005263-16
**Study phase**	IIa
**Sponsor**	PharmatrophiX Inc.
**Study Drug**	LM11A-31-BHS
**Manufacturer(s) of the investigational / reference product**	**ACE Pharmaceuticals BV**<br>PO BOX 1262<br>NL-3890 BB Zeewolde<br>The Netherlands
**Indication**	Alzheimer's disease
**Coordinating Investigator**	Prof. Dr. Anne Börjesson Hanson
**Study Centers**	20 in Austria, Germany, Czech Republic, Sweden and Spain

**Time Table**	Approval trial protocol:	June 2016
	First patient first visit:	January 2017
	Last patient first visit:	June 2019
	Last patient last visit:	December 2019
	Draft study report:	February 2020
	Final study report:	March 2018
**Duration of Treatment**	26 weeks	
**Study Objective(s)**	**Primary:**<br>To investigate the safety and tolerability of 200mg bid and 400mg bid doses of LM11A-31-BHS (free base) administered for a period of 26 weeks in comparison to placebo.<br>• Safety will be assessed through adverse event reporting, clinical laboratory, ECG and a standard range of patient	

	physical evaluations including a suicide severity rating scale (C-SSRS).<br><br>**Secondary/Exploratory:**<br>• Exploratory investigation of relevant biomarkers for AD and drug mechanism including regional brain glucose metabolism (18F-FDG-PET) and CSF measures.<br>• The assessment of exploratory clinical endpoints including a composite of the specific cognitive tests performed (the NTB composite standardized Z score).<br>• The investigation of the pharmacokinetics of 200mg bid and 400mg bid doses of LM11A-31-BHS (free base) administered for a period of 26 weeks.
**Evaluation criteria:**	**Safety variables**<br><br>Adverse events (AEs), Serious adverse events (SAEs), Clinical Diagnostics, Vital signs (blood pressure, heart rate, respiratory rate, body temperature), ECG, Laboratory assessment (hematology, biochemistry, coagulation, serology and urinalysis), Columbia-Suicide Severity Rating Scale (C-SSRS), MRI scans (analyzed by a central reader)<br><br>**Exploratory/ Candidate Efficacy variables**<br>• CSF-Biomarkers (tau, ptau, Aβ40, Aβ42, AchE activity)<br>• Regional brain glucose metabolism (^{18}FDG-PET)<br>• NTB:<br>o Digit Span Test<br>o Category Fluency Test<br>o COWAT<br>o Digit Symbol Substitution Test (DSST)<br>• ADAS-cog 13 items<br>• SPATIAL ORIENTATION AND LEARNING (Amunet)<br>• Geriatric Depression Scale (GDS)<br>• Clinical Global Impression Scale – Severity/Improvement (CGI-I/CGI-S)
**Patient population, diagnosis and main criteria for inclusion**	240 male and female patients with mild to moderate probable Alzheimer's disease according to McKhann (2011) criteria with ages 50-85 years (50-80 years in Czech Republic) and<br>• MMSE ≥18 and ≤26<br>• GDS <5<br>• Stable treatment with one of the acetylcholinesterase inhibitors donepezil (Aricept ®), galantamine (Razadyne®), or rivastigmine (Exelon) or the partial NMDA receptor antagonist with memantine (Namenda®) at least 3-months before baseline Visit or Combination of both treatments mentioned above

Inclusion criteria

1. Men and women (non-childbearing potential) with a diagnosis of Alzheimer's disease according to McKhann (2011) criteria
2. Age 50-85 years (50-80 in Czech Republic)
3. MRI or CT assessment within six months before baseline, corroborating the clinical diagnosis of AD and excluding other potential causes of dementia, especially cerebrovascular lesions (see exclusion criteria, number 3)
4. CSF AD specific biomarker profile; positive, defined as CSF Aβ42 <550 ng l^{-1} or a Aβ 40/42 ratio < 0,89.
5. Mild to moderate stage of Alzheimer's disease according to MMSE ≥18 and ≤26
6. Absence of major depressive disease according to GDS of < 5
7. Modified Hachinski Ischemic Scale ≤4
8. Formal education for eight or more years
9. Previous decline in cognition for more than six months as documented in patient medical records
10. Other Countries: "A caregiver available and living in the same household or interacting with the patient a sufficient time each week and available if necessary to assure administration of drug"

    Czech Republic: "Patients with moderate Alzheimer's Disease must have a caregiver available and living in the same household. Patients with mild AD must have a caregiver either living in the same household or a caregiver interacting with the patient, providing personal care for the patient during at least 10 hours per week and available if necessary to assure administration of drug"
11. Patients living at home or nursing home setting without continuous nursing care
12. General health status acceptable for a participation in a 6-month clinical trial
13. Ability to swallow capsules
14. Stable pharmacological treatment of any other chronic condition for at least one month prior to screening
15. Stable treatment with one of the acetylcholinesterase inhibitors donepezil (Aricept ®), galantamine (Razadyne®), or rivastigmine (Exelon) or the partial NMDA receptor antagonist with memantine (Namenda®) at least 3-months before baseline Visit or Combination of both treatments mentioned above
16. No regular intake of prohibited medications as noted in Section 11.8.
17. Signed informed consent by the patient, (in Germany and Czech Republic, examined and verified to be mentally capable by an independent physician/ neurologist) prior to the initiation of any study specific procedure. Signed consent of the caregiver (see inclusion criteria 10).

Exclusion criteria

1. Failure to perform screening or baseline examinations

2. Hospitalization or change of chronic concomitant medication one month prior to screening or during screening period
3. Clinical, laboratory or neuro-imaging findings consistent with:
   - Other primary degenerative dementia, (dementia with Lewy bodies, fronto-temporal dementia, Huntington's disease, Creutzfeldt-Jakob Disease, Down's syndrome, etc.)
   - Other neurodegenerative condition (Parkinson's disease, amyotrophic lateral sclerosis, etc.)
   - Cerebrovascular disease (major infarct, one strategic or multiple lacunar infarcts, extensive white matter lesions > one quarter of the total white matter)
   - Other central nervous system diseases (severe head trauma, tumors, subdural hematoma or other space occupying processes, etc.)
   - Seizure disorder
   - Other infectious, metabolic or systemic diseases affecting central nervous system (syphilis, present hypothyroidism, present vitamin B12 or folate deficiency, serum electrolytes out of normal range, juvenile onset diabetes mellitus, etc.)
4. A current DSM-IV diagnosis of active major depression, schizophrenia or bipolar disorder
5. Clinically significant, advanced or unstable disease that may interfere with primary or secondary variable evaluations, and which may bias the assessment of the clinical or mental status of the patient or put the patient at special risk, such as:
   - chronic liver disease, liver function test abnormalities or other signs of hepatic insufficiency (ALT, AST, Gamma GT, alkaline phosphatase > 2.5 ULN)
   - Respiratory insufficiency
   - Renal insufficiency (serum creatinine >2mg/dl) or creatinine clearance ≤ 30 mL/min according to Cockcroft-Gault formula). In case of creatinine clearance ≤30 mL/min, an alternative verification of the renal function must be completed using Cystatin C analysis. In case of normal level of Cystatin C, the patient can be included
   - Heart disease (myocardial infarction, unstable angina, heart failure, Cardiomyopathy within six months before screening)
   - Bradycardia (heart beat <50/min.) or tachycardia (heart beat >95/min.)
   - <u>For Austria, Germany, Spain and Sweden</u>: Hypertension (>180/95) or hypotension (<90/60) requiring treatment with more than three drugs
   - <u>For Czech Republic</u>: Hypertension (>160/95) or hypotension (<90/60) requiring treatment with more than three drugs

- AV block (type II / Mobitz II and type III), congenital long QT syndrome, sinus node dysfunction or prolonged QTcB-interval (males >450 and females >470 msec)
- Uncontrolled diabetes defined by HbA1c >8.5
- Malignancies within the last five years except skin malignancies (other than melanoma) or indolent prostate cancer
- Metastases

6. Disability that may prevent the patient from completing all study requirements (*e.g*. blindness, deafness, severe language difficulty, etc.)
7. Women who are fertile and of childbearing potential
8. Chronic daily drug intake of ≥ 14 days or expected for ≥ 14 days:
   - benzodiazepines (except lorazepam ≤ 1mg for sleeping disorders only), neuroleptics or major sedatives
   - Antiepileptics
   - Centrally active anti-hypertensive drugs (clonidine, l-methyl DOPA, guanidine, guanfacine, etc.)
   - Opioid containing analgesics
9. Nootropic drugs (except Ginkgo Biloba)
10. <u>Austria, Germany, Spain and Sweden</u>: Suspected or known drug or alcohol abuse, i.e. more than approximately 60 g alcohol (approximately 1 liter of beer or 500 ml of wine) per day, indicated by elevated MCV significantly above normal value at screening.

    <u>Czech Republic</u>: Suspected or known drug or alcohol abuse, i.e. more than approximately 20 g alcohol per day for females (500 ml of beer or 250 ml of wine) and 30g alcohol per day for males (approximately 750 ml of beer or 375 ml of wine) indicated by elevated MCV significantly above normal value at screening.
11. Suspected or known allergy to any components of the study treatments
12. Enrollment in another investigational study or intake of investigational drug within the previous three months
13. Any condition, which, in the opinion of the investigator, makes the patient unsuitable for inclusion
14. If patient is in any way dependent on the sponsor or the principal investigator or if the patient is accommodated in an establishment on judicial or administrative order

Methodology Multi-center, randomized, double-blind, placebo-controlled, parallel group study design with 3 treatment arms

**Number of patient visits**	Five visits (including screening)

Study Schedule

Visit	Week	Day
**V1** (Screening)	up to - 8	- 56 (-7d)
**V2** (Baseline)	0	1
**V3**	4	28 (+/-7d)
**V4**	12	84 (+/-7d)
**V5** (Final visit)	26	182 (+/-7d)

Investigational product, dose schedule and route of administration

Capsules: 200mg of LM11A-31-BHS free base (IMP)

Oral administration

Dose Schedule twice daily (morning & evening):

- **1 x 200mg of LM11A-31-BHS** and 1 x 200mg of placebo or
- **2 x 200mg of LM11A-31-BHS**

Total daily dosages are either 400mg beside placebo or 800mg LM11A-31-BHS free base.

The study drug is administered for 26 weeks, double-blind period.

Reference product, dose, schedule and route of administration

Capsules: 200mg of Placebo (microcrystalline cellulose)

Oral administration

Dose Schedule twice daily (morning & evening):

- **1 x 200mg of placebo** and 1 x 200mg of LM11A-31-BHS or
- **2 x 200mg of placebo**

Total daily dosages are either 400mg beside placebo or 800mg placebo (microcrystalline cellulose).

The placebo is administered for 26 weeks, double-blind period.

Adverse Events

AE spontaneous reporting and questionnaire, clinically relevant changes of laboratory values, vital signs, ECG

Total number of patients, statistical rationale provided

Fifty-one (51) patients per group are sufficient to demonstrate an effect size difference of 0.56 between either active dose or placebo control with 80% power and type 1 error rate 0.05 (two tailed).

Importantly this group size is also sufficient to estimate the difference in raw scores between the treatment means (x) with an

accuracy of 4.5 points with 95% two-sided confidence interval (i.e.: Lower limit margin = x-3.9, assuming pooled SD=10).

Allowing for an approximate drop-out rate of 15% then, a grand total of 240 patients (80 patients per group) will be recruited to the study at 20 centers in a ratio of 1:1:1 (200mg bid, 400mg bid, placebo).

In view of the stage of development and limited availability of prior drug use information a blinded data review will be performed to assess the variability of the primary efficacy endpoints. If appropriate after the data review the trial may be adapted and the required sample size may be further increased.

Statistical methods

Safety endpoints

All safety evaluations will be summarized as interval or categorical summaries, as appropriate. The overall incidence of adverse events, together with the top three most frequently reported adverse events, will be analyzed using a binary logistic model to demonstrate differences between the treatment groups and placebo.

Exploratory Efficacy Endpoints

All exploratory efficacy variables, including the NTB composite score, will be analyzed at the endpoint (Final visit). The analyses will involve the change from baseline calculated after 6 months (26 weeks) of treatment and will involve an Analysis Of Covariance Variance (ANCOVA) model incorporating the baseline score and country as covariates.

Additional analyses will involve evaluation of the time course of treatment response over the treatment period, using a repeated measures mixed model Analysis of Covariance (ANCOVA) for evaluation. MRI and 18F-Pet Scan variables will be analyzed in a descriptive and exploratory manner. Categorical endpoints will be analyzed using a Cochran Mantel-Haenszel (CMH-row mean scores difference), chi-squared test or Logistic regression models (as appropriate).

Output from statistical models (adjusted means and odds ratio estimates with 95% confidence intervals, statistics and associated p-values) will also be produced.

Summaries

All variables will be summarized using descriptive statistics, i.e. number (%) of patients for categorical variables and mean, SD (standard deviation), median, minimum, maximum for continuous variables. Descriptive statistics will be produced by treatment group.

Interim Data Review

A single overall statistical analysis will be performed at the conclusion of the project.

No formal interim analyses are planned and therefore no impact on the experiment wise type 1 error rate is envisaged.

However, in order to enable a review and reassessment of the project's power expected at the outset, a 'blinded' re-estimation of the pooled variability will be performed when the DSMB review is completed.

This will involve investigation of the primary cognitive composite of the NTB Z-score at week 26 with supporting information provided from each component item.

'Adaptation' in the design of this project will take the form of a potential increase in the total number of patients recruited and analyzed at the conclusion. This will be dependent on the demonstration of a larger variability estimate than that initially planned. The power calculations will then be repeated using the new pooled variability estimate, the sample size will be increased accordingly and the protocol will be amended.

No reduction of the sample size is intended to be done, if the variability estimate is smaller than initially planned.

Committees

A DSMB (Drug safety and monitoring board) will oversee and control the trial. This committee will be constituted of three independent clinical experts, one delegate of the Sponsor and one delegate of the clinical research organization (CRO). One independent statistician will be available to advise committee members. Prospectively defined sets of criteria, as outlined in the prospectively drafted "code of conduct" or charter, will be developed to guide the committee on anticipated risk decisions.

6 INVESTIGATORS AND STUDY ADMINISTRATIVE STRUCTURE

Internal Responsibilities:

Name	Function	Contacts
Anne Chun Longo	CEO	PharmatrophiX Inc.<br>2198 Sterling Avenue<br>Menlo Park, CA 94025<br>USA<br>anne@pharmatrophix.com
Dr. Frank Longo	Chairman of the Board and founder of PharmatrophiX	PharmatrophiX Inc.<br>2198 Sterling Avenue<br>Menlo Park, CA 94025<br>USA<br>frank@pharmatrophix.com

External Responsibilities: (CRO)

Manfred Windisch, PhD	Scientific Expert	NeuroScios GmbH<br>Willersdorferstrasse 7<br>A-8061 St. Radegund/Graz<br>AUSTRIA<br>Tel: +43 (0) 3132 40 444 - 11<br>Fax: +43 (0) 3132 40 444 - 20<br>mwindisch@neuroscios.com
Nikola Helmberg PhD	Project Manager	NeuroScios GmbH<br>Willersdorferstrasse 7<br>A-8061 St. Radegund/Graz<br>AUSTRIA<br>Tel: +43 (0) 3132 40 444 - 12<br>Fax: +43 (0) 3132 40 444 - 20<br>nhelmberg@neuroscios.com
David Fleet	Statistician	Data Magik Ltd<br>David Fleet M.Sc., C.Stat<br>Laburnum House<br>East Grimstead<br>Salisbury<br>UK SP5 3RT<br>Tel: +44 (0)1722 712972<br>Mob: +44 (0)7533 002238<br>dfleet@neuroscios.com
Daniel Funk	Study Safety Officer / Clinical Trial Pharmacovigilance	NeuroScios GmbH<br>Willersdorferstrasse 7<br>A-8061 St. Radegund/Graz<br>AUSTRIA<br>Tel: +43 (0) 3132 40 444 - 15<br>Fax: +43 (0) 3132 40 444 - 20<br>ctpv@neuroscios.com

External Responsibilities: (Clinic)

Name	Title	Address
Prof. Dr. Agneta Nordberg	Central Reader for PET Scans	Department of Neurobiology, Care Sciences and Society, Center for Alzheimer Research, Division of Translational Alzheimer Neurobiology,<br>Karolinska Universitetssjukhuset<br>Minnesmottagningen, M51<br>Huddinge<br>141 86 Stockholm
Prof. Dr. Reinhold Schmidt	Central Reader for MRI	Department of Neurology<br>1st subst. Head and Chair of Department<br>Auenbruggerplatz 22<br>8036 Graz
Prof. Dr. Anne Börjesson Hanson	Coordinating Investigator	Clinical Drug Research and Intervention Studies<br>Theme Aging, R&D<br>Geriatriska kliniken Karolinska Universitetssjukhuset<br>Minnesmottagningen, M51<br>Huddinge<br>141 86 Stockholm

Laboratory

	Name	Address
Inclusion and Safety parameters	Labor Dr. Spranger und Partner	D-85051 Ingolstadt, Lindberghstrasse 9-13; Germany
PK Samples and CSF analysis	Prof. Dr. Kaj Blennow	Clinical Neurochemical Laboratory<br>Inst. of Neuroscience and Physiology<br>The Sahlgrenska Academy at Gothenburg University<br>Mölndal Hospital<br>SE-431 80 Mölndal<br>Sweden
Plasma PK Samples	QUINTA-ANALYTICA s.r.o.	Prazska 18c/1486<br>102 00 Praha 10<br>Czech Republic

7 LIST OF ABBREVIATIONS AND DEFINITION OF TERMS

**ACh**	Acetylcholine
**AChE**	Choline Acetyltransferase
**AD**	Alzheimer's Disease
**ADAS-COG**	Alzheimer's Disease Assessment Scale
**ADDF**	Alzheimer Drug Discovery Foundation
**AE**	Adverse Event
**ALT**	Alanin-Aminotransferase
**ANCOVA**	Analysis of Covariance
**API**	Active Pharmaceutical Ingredient
**ApoE**	Apolipoprotein E
**AST**	Aspartate-Aminotransferase
**AV**	Atrioventricular
**bid**	Bis in diem, twice a day
**BMI**	Body Mass Index
**BP**	Blood Pressure
**BUN**	Blood Urea Nitrogen
**CEO**	Chief Executive Officer
**CHMP**	Committee for Medicinal Products for Human Use
**COWAT**	Controlled Oral Word Association Test
**CPK**	Creatine Phosphokinase
**CRF**	Case Report Form
**CRO**	Contract Research Organization
**CSF**	Cerebrospinal Fluid
**C-SSRS**	Columbia–Suicide Severity Rating Scale
**CT**	Computerized Tomography
**DLB**	Dementia with Lewy Bodies
**DSMB**	Drug Safety and Monitoring Board
**DSM-IV-TR**	Diagnostic and Statistical Manual of Mental Disorders, Fourth Edition
**DSST**	Digit Symbol Substitution Test
**ECG**	Electrocardiogram
**EDTA**	Ethylene Diamine Tetraacetic Acid
**EMA**	European Medicines Agency
**FDA**	Food and Drug Administration
**FDG-PET**	2-[18F]Fluoro-2-deoxyglucose positron emission tomography
**gamma-GT**	Gamma Glutamyltransferase
**GCP**	Good Clinical Practice
**GDS**	Geriatric Depression Scale
**GMP**	Good Manufacturing Practice
**IAP**	Interim Analysis Plan
**ICH-GCP**	International Conference on Harmonization – Good Clinical Practices

**IEC**	Institutional Ethics Committee
**IF**	Investigator's File
**IMP**	Investigational Medicinal Product
**IND**	Investigational New Drug
**IRB**	Institutional Review Board
**ITT**	Intention-to-treat
**IWRS**	Interactive Web Response System
**LOCF**	Last observation carried forward
**LDH**	Lactate dehydrogenase
**MAD**	Multiple Ascending Dose
**MCH**	Mean Corpuscular Hemoglobin
**MCV**	Mean Corpuscular Volume/ Cell Volume
**MedDRA**	Medical Dictionary for Regulatory Affairs
**mg**	Milligram
**MHIS**	Modified Hachinski Ischemic Scale
**MMSE**	Mini-Mental State Exam
**MRI**	Magnetic Resonance Imaging
**msec**	Millisecond
**MWM**	Morris Water Maze
**NGF**	Nerve Growth Factor
**ND**	"Not Done"
**NIA**	National Institute on Aging
**PD**	Pharmacodynamics
**PET**	Positron-Emission-Tomography
**PK**	Pharmacokinetic
**p.o.**	Per os (oral dose)
**PP**	Per protocol
**q.d.**	Quaque Die (Engl. Once Daily)
**QRS**	QRS Interval
**QT**	QT Interval
**QTc**	QT Interval (corrected)
**QTcB**	QT Interval (Bazett's correction)
**RPR**	Rapid Plasma Reagin
**RR**	Space between R-waves in ECG
**SAD**	Single Ascending Dose
**SAE**	Serious Adverse Event
**SAP**	Statistical Analysis Plan
**SD**	Standard Deviation
**SGPT**	Serum Glutamic Pyruvic Transaminase
**SI**	Sensitivity Index

**SMPC**	Summary of Product Characteristics
**SOP**	Standard Operating Procedure
**SSO**	Study Safety Officer
**SUSAR**	Suspected Unexpected Serious Adverse Reaction
**T½**	Elimination Half-life
**T4**	Thyroxine
**TEAE**	treatment emergent adverse event
**trk**	Tropomyosin receptor kinase
**TSPO**	Translocator Protein
**V**	Visit
**TSH**	Thyroid Stimulating Hormone
**WMA**	World Medical Association

8 INTRODUCTION

Current and planned AD trials remain largely focused on antibody- or enzyme-based strategies to lower Aβ levels with efforts now shifted to earlier, pre-dementia stages in high-risk individuals. The rationale is that Aβ-lowering treatments, which failed in mild to moderate stages, might be more effective in preventing progression if applied in earlier pre-dementia stages. One limitation to this approach is that Aβ lowering alone might not be sufficiently biologically robust in the overall context of AD mechanisms, which likely include additional age-related, inflammatory and other processes, and thus will have minimal efficacy, even at early stages. Indeed, the first results from a, pre-dementia phase 3 study testing application of the gantenerumab Aβ antibody, demonstrated no efficacy (Roche website, December 19, 2014 media release). A particularly concerning shortcoming of the requirement for pre-symptomatic status in current trial trends, is that the treatment needs of individuals who have already progressed to early and moderate dementia stages of AD (currently some 40M worldwide) will not be met. A second recent therapeutic approach is the development of antibodies designed to clear pathological forms of the tau protein after they have been formed. These trials are in early stages and no phase 2 efficacy data is yet available. Tau aggregate clearing treatments will unfortunately not affect other amyloid-related and other AD degenerative mechanisms, and moreover, finding ways to prevent the formation itself of toxic tau forms (as LM11A-31 appears to do) might be much more effective. A third emerging approach consists of attempts to inhibit brain inflammatory mechanisms, which include pathological activation of brain inflammatory cells including microglia and their release of toxic cytokines and complement factors. Preclinical studies in AD mouse models demonstrate that LM11A-31 inhibits microglial activation. Tau-based efforts remain in early stages, primarily in early pre-clinical animal studies and we are not aware of other orally bioavailable small molecules capable of inhibiting microglial activation in AD mouse models.

The proposed small molecule strategy addresses the challenges of therapies limited to either Aβ or tau and will change trial and clinical approaches because it addresses each of the above three mechanistic areas concomitantly; and moreover, accommodates quite well the important amyloid hypothesis but at the same time is not confined to it. If our pilot trial aim is achieved, an entirely novel target (p75 receptor) and mechanism of action (modulation of a broad and fundamental signaling network directly involved in AD) that concomitantly captures amyloid, tau and inflammatory mechanisms will be carried forward to larger scale AD trials.

Rationale. The rationale of the 26-week duration study is to test LM11A-31 for a maximum duration feasible for a first-time IIa safety and exploratory endpoint trial that allows optimal detection of effects on clinical and biomarker endpoints and any safety issues. The mechanism of action of LM11A-31 inhibiting Aβ effects on tau phosphorylation/misfolding/oligomerization, synaptic function as indicated by measurement of long-term potentiation (LTP), degeneration of synaptic spines, tau oligomer levels and other mechanisms indicates that cognitive effects (and indeed FDG-PET and CSF ptau/AChE activity effects) could very well be observed over weeks to months. Its mechanism of action of slowing degeneration might also start to be manifest at the 26-week time point, hence overall effects on endpoint measures at 6 months could be result of both short-term and mid-term disease and mechanism modification effects. An 18-month trial would be optimal for detection of disease slowing but it would not be feasible at this safety and proof of concept stage to conduct an 18 month trial.

Goal and Expected Outcomes. The goal of this AD Pilot is to conduct a prospective, double-blind, multicenter, phase IIa exploratory safety, feasibility and proof-of-concept trial in mild to moderate Alzheimer's disease patients with the orally bioavailable p75 neurotrophin receptor ligand LM11A-31-BHS dosed twice daily for 26 weeks. Successful completion of this trial will provide the safety, endpoint and statistical basis for the design and execution of a phase 2b/3 efficacy trial. It will also

bring to the AD field a much-needed new set of target mechanisms and will help pioneer the strategy of the concomitant targeting of multiple fundamental AD-related pathological processes.

LM11A-31-BHS targets an unmet need. There are no treatment options proven to slow progression of underlying neurodegenerative processes in AD – so called 'disease modifying' therapies. Multiple single-target phase 3 trials of amyloid-beta lowering treatments failed to demonstrate therapeutic effects in mild to moderate AD pointing to the possibility that such strategies might be effective if applied in earlier, pre-dementia stages. However, the recent reported failure of the first such pre-dementia phase 3 trial, testing the amyloid antibody gantenerumab (Roche press release), further indicates that additional underlying AD mechanisms, such as those involving tau, inflammatory and other processes will need to be targeted. The p75 neurotrophin receptor is linked in multiple ways to signaling networks that play critical roles in synaptic failure and degeneration in AD and that involve each of these mechanistic areas. Receptor validation studies demonstrate that its default degenerative signaling plays a critical enabling role in amyloid-induced degenerative signaling. Its expression is upregulated in AD and it is expressed essentially by all of the neuronal populations affected in AD. LM11A-31 is a first-in-class orally bioavailable, small molecule p75 receptor ligand with outstanding brain penetration. With prior ADDF, R21 and NIA U01 funding and published work, it has been shown to engage its target in vivo and to modulate p75 signaling such that it inhibits amyloid-induced: degenerative signaling, synaptic failure, and neuronal degeneration; tau pathological phosphorylation, misfolding, mislocalization and oligomer formation; and microglial activation. No effects on amyloid levels were found. Successful pre-IND studies made possible FDA approval for phase 1 testing. A phase 1 safety trial with SAD and MAD arms in young and elderly normal subjects, successfully conducted on time and on budget by PharmatrophiX, demonstrated no significant adverse events at exposures some 7-8-fold higher than exposures demonstrating maximum efficacy in AD mouse models.

Specific Aim. Conduct a prospective, double-blind, multicenter, phase IIa exploratory safety, feasibility and proof-of-concept trial in mild to moderate Alzheimer's disease patients with the orally bioavailable p75 neurotrophin receptor ligand LM11A-31-BHS dosed twice daily for 26 weeks.

Specific Objectives. The proposed phase IIa safety and proof-of-concept trial will constitute the first testing in AD patients. The trial will include a 80 patient vehicle group, and 80 patients each at a low (200 mg/bid) and high (400 mg/bid) dose incorporating an adaptive design, to allow eventual adaptation in the dosing regimen, based on results from a scheduled interim analysis. The adaptive design will also allow potential expansion of the trial size. The trial will further assess LM11A-31 safety along with its ability to affect the following endpoints: cognitive and other clinical measures; biomarkers including FDG-PET; along with CSF amyloid, tau, p-tau and AchE activity. Statistical and power analysis of endpoint data will examine trends for effects, relationships between clinical assessments and biomarkers and will make possible the design of a phase 2b/3 efficacy trial.

8.1 Clinical Experience

Phase 1 single dose study. Extensive preclinical studies in animals and a clinical phase I trial on healthy human subjects showed that LM11A-31 is generally well tolerated and safe. The phase I clinical trial was a randomized, double-blind, placebo-controlled, single ascending dose (SAD) and multiple ascending dose (MAD, 10 days) study in healthy young male and elderly male and female subjects.

In summary, LM11A-31 at doses equivalent to those likely to be well tolerated in additional human studies, has been found to be effective across: i) three different AD mouse models; ii) in a very late-stage AD mouse model; and iii) in a non-transgenic aged-mouse model. These three exceptional qualities constitute an exceptionally strong profile for a small molecule poised to enter human AD

trials. Indeed, we are not aware of another small molecule or antibody that has been shown to reverse fundamental morphological neurodegenerative endpoints in late-stage AD models, particularly a small molecule or antibody ready for human AD trials.

Summary of phase 1 safety and PK study:

- LM11A-31 and its metabolite exhibited linear pharmacokinetics after single oral doses as exposure was proportional to dose over a range from 100-900 mg of free base.
- Upon multiple dosing, LM11A-31 and its major metabolite aminoethyl morpholine (AEM) appears to reach steady state by approximately 5 days post dose.
- In elderly adults, there is an apparent food effect for both parent and metabolite, with fed subjects having a higher LM11A-31 exposure than fasted subjects.
- Preliminary results suggest a gender difference in LM11A-31 exposure, with female subjects having a greater exposure than males when receiving unit doses.
- LM11A-31-BHS, administered as single doses of 100 mg, 300 mg, 600 mg, and 900 mg free base, and as multiple doses of 300 BID and 600 mg free base QD, was generally safe and well tolerated.
- Following treatment with a single dose of LM11A-31-BHS, 1 of 40 subjects experienced a total of 1 treatment emergent adverse event (TEAE), (postural dizziness considered possibly related to study drug).
- During administration of multiple doses of LM11A-31-BHS, 7 of 16 subjects experienced a total of 8 TEAEs, 6 of which were considered unrelated to study drug. The remaining 2 events were considered possibly (flatulence) or remotely (nocturia) related to study drug.
- There were no deaths, (serious adverse events) SAEs, severe TEAEs, and no subjects were discontinued because of an AE. All TEAEs were mild.
- No clinically significant laboratory results, physical examination findings, vital signs, ECG findings, or positive scores on C-SSRS assessments were observed.

8.2 Risk/Benefit Assessment

8.2.1 Principal hazards of the medicinal product identified in non-clinical studies

In rats and dogs, LM11A-31-BHS was well tolerated at single doses of 1000 and 2000 mg/kg. In 28-day studies in rats and dogs, no observed adverse effect levels (NOAELs) were 600 and 1000 mg/kg, respectively. Lethality in rats (2000 mg/kg) was associated with renal toxicity (tubular necrosis). LM11A-BHS was negative in an *in vitro* mouse lymphoma gene mutation test and in an *in vivo* micronucleus test in rats up to 2000 mg/kg/day (PMX419UF-124192/LM11A31-101 – CLINICAL STUDY REPORT). In 6-month toxicity non-clinical studies with both **rats** and **dogs** it could be clearly demonstrated that doses of 750 mg/kg BID (1500 mg/kg/day) in rat and 1000 mg/kg QD in dog were well-tolerated and did not cause unexplained mortality or renal toxicity.

It was further shown that the early acute mortality in rats and dogs in previous studies was caused by the marked acidity (pH of 0.98-1.7) of the dosing solutions. Inadvertent aspiration of these highly acidic dosing solutions led to acute pulmonary compromise and death. Therefore, dose solution pH adjustments were made, and the 6-month toxicity studies were uneventful.

In a **6-Month GLP Dog Study** (CiToxLab 15/101-118K), LM11A-31-BHS was given to male and female Beagle dogs at initial doses of 100, 300, 600 or 1000 mg/kg once daily by gavage for 26 weeks. Severe vomiting at 600 and 1000 mg/kg was observed, showing a clear dose-response. Vomiting was also noted in the other treated groups but with lower frequency and severity. On Study Day 4, one male dog treated with 1000 mg/kg was found dead. The clinical data (vomiting)

and macroscopic findings including non-collapsed lungs and foamy material within the trachea are consistent with acute aspiration leading to acute pulmonary compromise and death. Following this event, dosing was suspended pending an investigation of the dosing formulation pH, which revealed that the dosing solutions were all strongly acidic with concentration response. The dosing solution pH was subsequently adjusted to pH = 4.5-6.0 with NaOH. Dosing was restarted with adjustments to dose, dose concentrations and pH. The doses in the restarted study were 0, 100, 300, 500 and 750 mg/kg/day for 26 weeks; there was no further morbidity or mortality in the study. Treatment-related clinical signs consisted of reversible salivation, vomiting and liquid feces in both sexes at doses of 300 mg/kg and higher. The frequency and severity of most of the clinical signs were dose dependent and erratic. Reversible decreases in mean body weights or body weight gains with inconsistent corresponding changes in food consumption were also evident at doses of 300 mg/kg and higher. Minimal hepatic extramedullary hematopoiesis (EMH) was noted in some animals at 500 and 750 mg/kg. EMH was present in only one high dose animal following recovery suggesting that this effect was reversible. Toxicokinetic evaluations at steady-state indicated that AUC_{0-t} for both parent and metabolite AEM increased in approximate proportion with dose from 100 to 750 mg/kg/day, but increases in C_{max} were less than dose-proportional. No accumulation of parent or metabolite occurred following repeated daily oral administration of LM11A-31-BHS across the entire dose range, and no significant gender differences in exposures of either parent or metabolite were observed.

In a **6-Month GLP Rat Study** (CiToxLab 15/101-118P), LM11A-31-BHS was given to male and female Sprague-Dawley rats (200, 500, 1000, 1300, 1500 mg/kg/day) in divided doses twice daily by oral gavage for 26 weeks. As in the dog study, the dose solutions were adjusted to pH of 4.5-6. There was no mortality, clinical signs of toxicity or changes in food consumption, body weight, or neurological function based on a detailed assessment during the study attributed to treatment with LM11A-31-BHS administration. No toxicologically important changes in ophthalmology, hematology, clinical chemistry, coagulation, urinalysis, or macroscopic and histopathological examinations occurred in LM11A-31 treated animals. Reversible, minor increases in liver weights at doses of 500 mg/kg and higher and kidney weights at doses of 1000 mg/kg/day occurred without any correlating histopathological change (IND 113708, Serial No. 0013, PTX, LM11A-31-BHS, confidential).

8.2.2 Suggested mechanisms and exposure thresholds for such untoward effects exposure margins in the suggested trial based on systemic AUC and Cmax rather than dose

In the **6-month dog toxicity study**, LM11A-31-BHS was well tolerated at all dose levels. Minimal and reversible ovarian changes in the high-dose group were attributed to body weight loss and decreased food consumption and were not considered a direct effect of the test article. The NOAEL in both sexes was considered to be 750 mg/kg/day which corresponded to gender mean Cmax value for LM11A-31 of 17,250 ng/mL and an AUC0-24hr of 31,900 ng.h/mL during Week 26.

The NOAEL in both sexes of **Sprague-Dawley rats** was considered to be 1500 mg/kg/day (750 mg BID), which corresponded to a gender mean C_{max} value for LM11A-31of 8980 ng/mL and an AUC_{0-24hr} of 36,600 ng•h/mL during Week 26.

Table 1 contains the safety margin information based on AUC and Cmax exposures for 600 mg BID in humans, implying even greater safety margins for 400 mg BID. In the current phase 2a study human participants will receive 400mg BID (800 mg LM11A-31-BHS per day) as the highest dose. Setting exposure limits based on the maximum non-lethal dose, $1/10^{th}$ the exposure at the non-lethal dose (NOAEL) in the **6-month** studies would conservatively yield a clinical target for C_{max} of 898/1725 ng/mL and for AUC of 3660/3190 ng•hr/mL based on the rat and dog non-lethal dose (aka the NOAELs). Alternatively, $1/10^{th}$ safety margins from the NOAEL are generally applied for selecting starting doses for Phase 1; dose escalation in Phase 1 is normally dependent upon clinical safety and pharmacokinetics, within reason; 10-fold safety margins are not continuously applied

during dose escalation and development for compounds that appear relatively safe in toxicology studies. Rather it is conventional, if supported by the nonclinical data, to dose-escalate in humans towards the NOAELs until a dose-limiting toxicity is observed. Nevertheless, our safety margins based on data from our 6-month toxicology studies support human doses of 400 mg BID (Table 1).

Table 1: LM11A-31--PK Data and Safety Margins Based on the 6-Month Toxicology Program

	**Gender Mean Pharmacokinetic Data**		**Human Safety Multiple 600mg BID**
	**C_{max} (ng/mL)**	**AUC_{0-24hr} (ng•hr/mL)**	**C_{max}/AUC Multiple**
Elderly Human Volunteers-<br>600 mg QD-Day 10*	1372	2468**	
Rat NOAEL 750 mg/kg BID (1500 mg/kg/day)	8980	36,600	7x/15x
Dog NOAEL 750 mg/kg/day QD	17,250	31,900	13x/13x

* Average of males and females, as there was no gender difference

** BID estimate (2x QD AUC)

8.2.3 Possible risks to trial subjects in the proposed study

In livers, minimal diffuse hypertrophy of periportal hepatocytes was observed in male and female rats at the terminal necropsy in a rat 28-day study. Hepato-renal histological lesions were reversible, and there were no lesions in rats given 200 or 600 mg/kg. Lethality in rats (2000 mg/kg) was associated with renal toxicity (tubular necrosis), which was a very high dose given, far beyond the highest dose used for humans. Reversible, minor increases in liver weights at doses of 500 mg/kg and higher and kidney weights at doses of 1000 mg/kg/day occurred without any correlating histopathological change in a 6-Month GLP Rat Study (CiToxLab 15/101-118P)(IND 113708, Serial No. 0013, PTX, LM11A-31-BHS, confidential).

In a 6-Month GLP Dog Study (CiToxLab 15/101-118K) minimal hepatic extramedullary hematopoiesis (EMH) was noted in some animals at 500 and 750 mg/kg. EMH was present in only one high dose animal following recovery suggesting that this effect was reversible.

In a phase 1 safety study, the administration of LM11A-31-BHS as single doses of 100 mg, 300 mg, 600 mg, and 900 mg free base, and as multiple doses of 300 BID and 600 mg free base QD, was generally safe and well tolerated. There were no clinically significant abnormalities in clinical laboratory tests, vital signs, ECGs, physical examinations, or Columbia Suicide Severity Rating Scale (C-SSRS) assessments, as described below under safety assessment.

Overall, the risk to benefit assessment is considered as highly favorable, given the greater than 10 x safety margins expected in human PK, based on data with 6 months of testing in rats and dogs, and having reached 900 mg in humans in 10-day testing.

The rationale for the use of 800 mg (400 mg bid) in the human phase 2 study is that the half-life in plasma is short at about 1 hour, and in animal brains about 3 hours. To date there are no data available for the half-life in human brains. According to the fact that minor increases in liver weights at doses of 500 mg/kg and higher were reversible, and kidney weights at doses of 1000 mg/kg/day occurred without any correlating histopathological change in a 6-Month GLP Rat Study, and due to the fact that there were no clinically significant abnormalities in clinical laboratory tests in a phase 1 study with healthy young and elderly subjects, it is very unlikely to expect any liver or renal toxicity in humans in the phase 2a study. However utmost care will be taken during the study, and blood values with special focus on liver enzymes and relevant renal parameters will be cautiously monitored during the study, as also described under 8.2.4.

Of note is that a Study Safety Officer (SSO), provided by NeuroScios, as well as a Medical Monitor (MM), designated by the sponsor, will be available for constant surveillance of subject safety during the whole period of the study, as described in more detail under 8.2.4.

Toxicity Assessment

In a GLP 28-day toxicity study in rats with a 14-day recovery period, conducted with once daily oral doses of 200, 600, and 2000 mg/kg, lethality, reversible renal tubular necrosis, and reversible hepatocellular hypertrophy were observed in survivors at 2000 mg/kg/day. One female rat died and had the most severe renal lesions. There were no adverse effects and no effects on ophthalmologic, clinical pathologic, macroscopic, and microscopic evaluations in animals receiving 600 mg/kg. The NOAEL was 600 mg/kg for 28 days. Test article-associated microscopic findings were seen in the kidneys and the livers of 2000 mg/kg males and females (15/15). In the kidneys, multifocal bilateral coagulative necrosis (minimal or mild) was observed in the female found dead on day 4, the male euthanized moribund on day 10, and in males at the terminal necropsy. In the livers, minimal diffuse hypertrophy of periportal hepatocytes was observed in males and females at the terminal necropsy. Hepato-renal histological lesions were reversible. There were no lesions in rats given 200 or 600 mg/kg (Ref. IB, p.31-33 4-9: 28-Day Rat Study Design (200, 600, 2000 mg/kg QD). In a subsequent 28-day rat study, no renal toxicity was identified at doses up to 2000 mg/kg either once daily (QD) or 1000 mg/kg BID; the NOAEL was 1500 mg/kg/day (750 mg/kg BID) in this study. Importantly, there was no morbidity/mortality at 1000 mg/kg BID, and there was no evidence of renal tubular necrosis or any changes in a broad panel of renal biomarkers at doses of 2000 mg/kg. In a 28-Day GLP Rat Study (SRI-M173-15) 750 mg/kg BID was identified as the NOAEL. In **dogs**, single oral doses up to 2000 mg/kg were well tolerated. BID oral dosing at 1000 mg/kg/dose for 7 days was generally well tolerated. In a GLP 28-day toxicity study in dogs with a 14-day recovery period, conducted with once daily oral doses of 100, 300, and 1000 mg/kg, there was salivation and vomiting at 300 and 1000 mg/kg/day; LM11A-31-BHS was generally well tolerated. There were no effects on ophthalmologic, electrocardiographic, clinical pathologic, macroscopic, and microscopic evaluations. The NOAEL was 1000 mg/kg. LM11A-31-BHS was negative in a bacterial reverse mutation test, mouse lymphoma gene mutation test, and in an *in vivo* micronucleus test in rats at doses of 2000 mg/kg/day (LM11A-31-BHS_IVB_20160901_v2.3, under 4.4 Toxicology). Under 8.2.1 it was stated that in a **6-Month GLP Rat Study** (CiToxLab 15/101-118P), LM11A-31-BHS was given to male and female Sprague-Dawley rats (200, 500, 1000, 1300, 1500 mg/kg/day) in divided doses twice daily by oral gavage for 26 weeks. There was no mortality, clinical signs of toxicity or changes in food consumption, body weight, or neurological function based on a detailed assessment during the study attributed to treatment with LM11A-31-BHS administration. No toxicologically important changes in ophthalmology, hematology, clinical chemistry, coagulation, urinalysis, or macroscopic and histopathological examinations occurred in LM11A-31 treated animals. Reversible, minor increases in liver weights at doses of 500 mg/kg and higher and kidney weights at doses of 1000 mg/kg/day occurred without any correlating histopathological change (IND 113708, Serial No. 0013, PTX, LM11A-31-BHS, confidential).

In a **6-Month GLP Dog Study** (CiToxLab 15/101-118K), LM11A-31-BHS was given to male and female Beagle dogs at 0, 100, 300, 500 and 750 mg/kg/day for 26 weeks, and there was no further morbidity or mortality observed in the study.

These findings were followed up with careful monitoring in the SAD/MAD program, including exploring many renal urinary biomarkers, which showed that there was no evidence of renal impairment in the clinical program at doses up to 600 mg QD in elderly volunteers over 10 days.

In summary, the toxicology studies demonstrated that the compound was extremely safe, supported by the high NOAEL doses, and that there were no severe side effects in the phase 1 clinical trials.

Pharmacokinetic Assessment

LM11A-31 was rapidly absorbed after oral administration in mice, rats, and dogs with an observed maximum concentration in plasma (C_{max}) occurring at the first blood sampling time point of 5 to 20 minutes. The oral bioavailability was moderate ranging between 22% in mice to 64% in rats. Approximate dose proportional increases in systemic exposure were observed in rats over a dose range of 5 to 500 mg/kg. LM11A-31 had a high plasma clearance (CL) and high volume of distribution in mice, rats, and dogs. The plasma CL was greater than the liver blood flow in all species studied indicating extrahepatic CL of the compound. The volume of distribution was greater than the total body water indicating extensive tissue distribution. The apparent terminal elimination t1/2 after IV administration was short in all species studied ranging from 0.36 hours in rats to 1.4 hours in dogs (Encl 6 LM11A-31-BHS_IB_20160901_v2.3 sig). Incubation of LM11A-31 with blood and cryopreserved hepatocytes from mice, rats, dogs, monkeys, and humans, and analysis of plasma samples collected from rats and dogs indicated that the major metabolic pathway of LM11A-31 was amide hydrolysis forming L-isoleucine and 4-(2aminoethyl)-morpholine (AEM). AEM as a **major metabolite** in all species tested was pharmacologically inactive. There was limited plasma protein binding in humans, no P450 inhibition and a limited amount of parent drug was recovered in urine. There were no pharmacokinetic or metabolic risks identified in rats or dogs (Encl 6 LM11A-31-BHS_IB_20160901_v2.3 sig).

8.2.4 Principal hazards of the medicinal product identified in clinical studies

In the clinical studies, the SAD/MAD Phase 1 study with healthy subjects, there was no evidence of any toxicity at doses up to 600 mg QD in elderly volunteers for 10 days.

Summary of Safety assessment in a phase 1 study with Healthy Young and Elderly Subjects

SAD: No deaths, SAEs, severe treatment-emergent adverse events (TEAEs), or withdrawals due to TEAEs occurred during the SAD part of the phase 1 study, as mentioned under summary of Phase I and PK study in chapter 8.1. Overall, 3 of 52 subjects (5.8%) experienced a total of 3 treatment emergent adverse events (TEAEs). Of these 3 subjects who experienced TEAEs during the SAD part of the study, 2 were treated with placebo, and 1 was treated with 100 mg LM11A-31-BHS free base. The subject who received LM11A-31-BHS experienced mild dizziness that was considered possibly related to treatment with LM11A-31-BHS. No TEAEs were reported in LM11A-31-BHS treated subjects in SAD Cohorts 2-6, and no subjects in the SAD part of the study experienced severe or moderate TEAEs. Additionally, none of the TEAEs that occurred during the SAD part of the study were considered probably related to treatment with study drug.

MAD: A total of 10 TEAEs occurred in 8 of the 20 subjects (40.0%) enrolled into the MAD part of the study. Seven subjects treated with LM11A-31-BHS and 1 subject treated with placebo experienced TEAEs during the MAD part of the study. The most frequently reported TEAEs, by system organ class were gastrointestinal disorders (4 subjects [20.0%]), including the preferred terms constipation (2 subjects [10.0%]), flatulence (1 subject [5.0%]), and gingival pain (1 subject [5.0%]). Overall, 6 of the 8 subjects who experienced TEAEs during the MAD portion of the study experienced TEAEs that were considered unrelated to treatment with study drug. The remaining 2 subjects who experienced TEAEs during the MAD experienced TEAEs that were considered remote (nocturia) or possibly (flatulence) related to treatment with study drug. No subjects experienced TEAEs that were considered probably related to treatment with study drug. All of the TEAEs that occurred during the MAD part of the study were mild. In subjects treated with 300 mg LM11A-31-BHS free base BID for 10 days, 5 subjects (62.5%) experienced 5 TEAEs. None of these TEAEs, by preferred term, occurred in more than 1 subject, and all were considered unrelated to treatment except for 1 (flatulence), which was considered possibly related to LM11A-31-BHS. All TEAEs that occurred in subjects treated with 300 mg LM11A-31-BHS free base BID for 10 days were mild. In subjects treated with 600 mg LM11A-31-BHS free base QD for 10 days, 2 subjects (25.0%) experienced 3 TEAEs. None of these TEAEs, by preferred term, occurred in more than 1 subject. One

subject experienced TEAEs that were considered unrelated to treatment, and the other subject experienced a TEAE (nocturia) that was considered remotely related to treatment with LM11A-31-BHS. All TEAEs that occurred in subjects treated with 600 mg LM11A-31-BHS free base QD for 10 days were mild.
In summary, no deaths, SAEs, severe TEAEs, or withdrawals due to TEAEs occurred during the SAD part of the study. No deaths, SAEs, severe TEAEs, or withdrawals due to TEAEs occurred during the MAD part of the study. During administration of multiple doses of LM11A-31-BHS, 7 of 16 subjects (43.8%) experienced a total of 8 TEAEs. During administration of multiple doses of placebo, 1 of 4 subjects (25.0%) experienced a total of 2 TEAEs. The majority of TEAEs that occurred during the MAD part of the study (6 subjects [30.0%]) were unrelated to treatment with study drug and all were mild. There were no clinically significant abnormalities in clinical laboratory tests, vital signs, ECGs, physical examinations, or Columbia Suicide Severity Rating Scale (C-SSRS) assessments.
Since the majority of TEAEs in the MAD study were unrelated to the IMP, it is not assumed that even with an increased dose of 400 BID there will be any new TEAEs.

8.2.5 Measures taken to monitor and manage predicted risks

During the whole period of the study, a Study Safety Officer (SSO), provided by NeuroScios, as well as a Medical Monitor (MM), designated by the sponsor will be available for constant surveillance of subject safety. An independent Data Safety Monitoring Board (DSMB) will periodically oversee subject's safety and will be immediately informed in case of any proposed serious adverse reactions throughout the study. In case of any serious concerns regarding the safety of the enrolled subjects, the DSMB will immediately stop the clinical trial.
The first meeting of the DSMB will be scheduled approximately 6 months after study initiation, provided that no suspected unexpected serious adverse reaction (SUSAR) has occurred beforehand. Interim meetings will take place on a 6-months basis afterwards, in order to evaluate possible reactions to LM11A-31-BHS and to evaluate reported (serious) adverse events ("AE/SAE") and define their relevance. The second meeting of the DSMB will be scheduled to take place after the Database Lock of the clinical trial, and after all treatment arms assigned to subjects are disclosed. This meeting will take place to evaluate possible reactions to LM11A-31-BHS, to evaluate reported (serious) adverse events ("AE/SAE") and define their relevance, and to provide a report to establish the updated toxicity profile of LM11A-31-BHS. Apart from these scheduled meetings, the DSMB will be involved if there is a suspected case of a SUSAR, and any subject death with a suspected causal relationship to IMP administration. The DSMB will evaluate the event on the basis of a report provided by the investigator and all relevant data required for a full evaluation. The DSMB will provide a written report detailing its judgment on the nature of the event and its causal relationship to LM11A-31-BHS administration. In addition, the DSMB will provide Pharmatrophix, Inc. with a recommendation regarding continuation of the clinical trial as well as possible additional safety measures to be taken. Other DSMB meetings may be requested by Pharmatrophix, Inc., Investigators or DSMB board members. Every reasonable effort shall be made to convene the entire DSMB for each scheduled meeting. The timing of meetings will attempt to accommodate the calendars of the three members. The DSMB will convene in person or by teleconference.
At each meeting, the members of the DSMB will review following data:

- Serious Adverse Events (SAEs)
- Clinically significant abnormal lab findings (especially liver enzymes, urinary renal biomarkers)
- Withdrawals due to AE/SAE
- ECG changes triggering withdrawal
- other data submitted by Pharmatrophix, Inc. and/or Investigators for DSMB review

9 RESEARCH STRATEGY

According to the Alzheimer's Association, some 5.5million individuals in the United States (US) and > 40 million in the world have Alzheimer's disease (AD) and more than 95% of these are aged 65 years or older. One in 8 Americans age 65 and older, and almost one-half of those aged 85 and older have AD. Mild cognitive impairment is in most cases, an early stage of AD. Of the approximately 40 million Americans older than 65, 10-20% of those who do not have a dementia have mild cognitive impairment, thus more than 10 million Americans are likely suffering from early and late stages of AD. In addition to the incalculable human costs for individuals and their families, AD cost the US an estimated $183 billion dollars in 2011. With aging demographics, the number of affected individuals and the cost of care are in the early stages of an exponential rise with costs expected to reach $1.1 trillion by 2050.

To date, there are no effective disease-modifying treatments for AD. The goal of achieving a therapy that slows the underlying progression of neuronal degeneration has largely focused on efforts to reduce the accumulation of amyloid beta (Aβ) either by inhibition of enzymes necessary for Aβ production or by immunization against the Aβ peptide. A critical barrier to progress in this field is that a wide range of mechanisms that are not necessarily addressed by simply lowering Aβ (such as those involving tau and inflammation) are likely additional significant contributors to AD related synaptic failure and degeneration. It has been difficult to develop therapies that modulate a sufficiently broad range of key underlying degenerative mechanisms. In early studies, our team hypothesized that the signaling network of the p75 neurotrophin receptor has multiple points of integration with key degenerative signaling networks involved in AD (see Figure 1; Nguyen et al., 2014[1]). Along with receptor validation studies (described below), an orally bioavailable, highly brain penetrant small molecule ligand (LM11A-31) was developed that modulates signaling of the p75 neurotrophin receptor and thereby affects the broad and fundamental signaling networks directly involved in AD and linked to the key mechanisms involving amyloid, tau and microglial activation. LM11A-31 is a non-peptide, orally bioavailable, highly brain penetrant (brain to plasma ratio > 1), water soluble small molecule (MW = 243) ligand which modulates signaling of the p75 neurotrophin receptor. It was originally identified by the Longo-Massa research team at UCSF as part of an in silico screening program searching for small molecules with favorable CNS drug features that incorporate the shape and physical chemistry of the loop 1 p75 receptor binding domain of neurotrophins and hence might bind to p75 and function as p75 ligands[2]. The structure of LM11A-31 is shown below in Figure 2 and its physical and pharmacological properties are further described in the Investigators Brochure. LM11A-31 was found to bind to p75 and prevent proNGF from binding to p75, but did not bind to Trk neurotrophin or Cerep panel receptors, its lack of

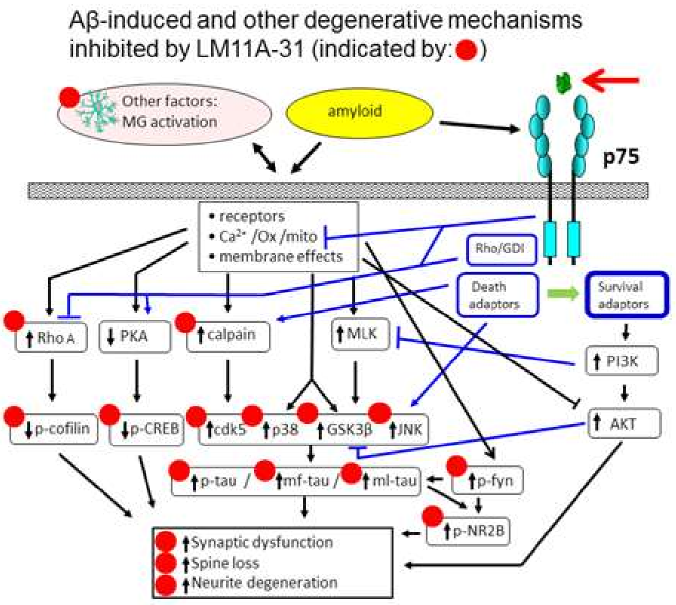

Figure 1: Aβ-induced and other degenerative mechanisms inhibited by LM11A-31-BHS

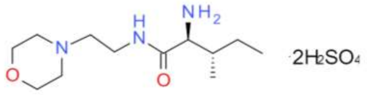

Figure 2: Chemical Structure of LM11A-31-BHS

binding to Trk receptors accounts for its inability to promote pain mechanisms as nerve growth factor does through its TrkA receptor[3].

P75 is expressed by most of the neuronal populations vulnerable in early and advanced stages of AD, its expression is up regulated in AD and it plays a potent role enabling amyloid beta (Aβ)-induced degenerative signaling within neurons (Figure 1).

In terms of target validation, in vitro studies in which Aβ oligomers are added to matured hippocampal neurons derived from wild type or p75-/- neurons; and in vivo studies, in which APP-L/S mice are crossed with p75-/- mice demonstrate that removal of wild type p75 prevents Aβ-induced neurite degeneration[4]. This finding is consistent with the known profile of p75 which in its default unliganded mode or in the presence of its predominant CNS ligand, proNGF, promotes degenerative signaling. Removal of functional p75 (i.e. p75-/- mice) prevents its degenerative signaling, and targeting it with our LM11A-31 small molecule ligand shifts its signaling patterns from pro-degenerative to pro-survival and in doing so inhibits Aβ-induced signaling, neural degeneration and synaptic dysfunction. One of the several strong points of our strategy is that we acknowledge that Aβ has many neuronal targets and that we are not dependent on blocking its interaction with one specific target but instead inhibiting its ability to induce a broad pattern of degenerative signaling regardless of its specific target(s). In vitro studies demonstrate that LM11A-31 blocks the following oligomeric Aβ effects: activation of four tau S/T kinases (GSK3β, JNK, CDK5, p38); excess RhoA/cofilin activation; excess FYN activation; excess tau phosphorylation/missfolding/ mislocalization/ oligomerization; and impairment of LTP in HC slices[3, 5], (also Yang et al, 2014 Society for Neuroscience Abstracts Online 2014). Unlike application of specific inhibitors such as those targeting a tau or FYN kinases, LM11A-31 does not inhibit the baseline/physiological function of these signaling/enzyme proteins and is therefore less likely to cause the side effects that might be caused by direct inhibitors of FYN or tau kinases. Indeed, the AD therapeutic field has been hampered by side effects occurring through inhibition of physiological enzymes. LM11A-31 protective effects are specific to p75 in that these effects are lost in p75-/- neurons and p75 blocking antibody inhibits its effects. The Cerep screen for binding to other receptors is negative. LM11A-31 has a favorable PK profile including brain to plasma ratios well above 1.0 and a brain half-life of 3-4 hours following a single oral administration. In vivo target engagement with p75 was verified by its ability to induce p75 cleavage, a proximate step in binding by its NGF/proNGF natural ligands. It has been applied in vivo, through once-daily oral dosing, to three AD mouse models (APP-L/S, Tg2576 and Ts65DN) at stages in which pathology is already started and in each case found to inhibit neuronal degeneration as determined by blinded quantitative, unbiased, morphological imaging techniques [1, 3, 6]. In the APP-L/S and Ts65DN models, behavioral deficits (MWM-DMPT, NOR, CFC, Y-maze, Impaired Sociability) were assessed in a core behavioral laboratory and found to be reversed, consistent with our signaling model and HC slice LTP data. In very late stage APP-L/S mice, a one month treatment

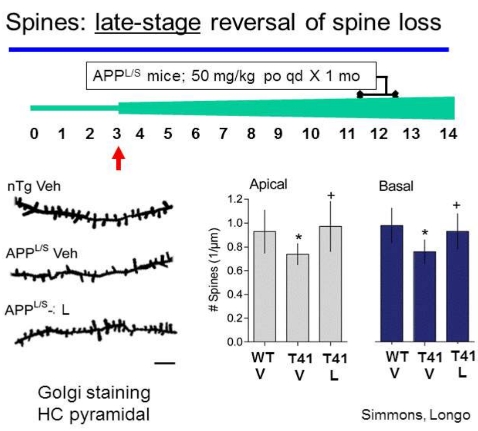

Figure 3: Late-stage reversal of dendritic spine loss

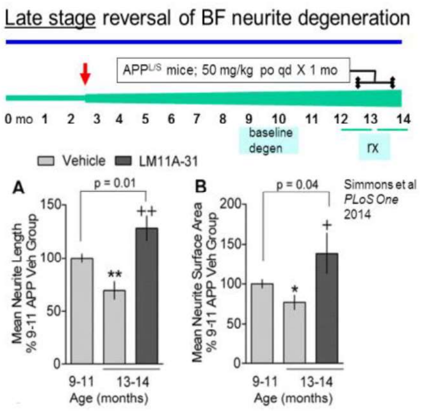

Figure 4: Late-Stage reversal of BF neurite degeneration

period led to an actual reversal of basal forebrain neurite degeneration[6] and a reversal of hippocampal neuron spine loss[3] (Figures 3 and 4) indicating a particularly robust biological effect. LM11A-31 has also been shown to reverse age-related basal forebrain cholinergic neuron degeneration in 2-year-old wild type mice (Xie et al, Society for Neuroscience Online Abstracts 2013), hence its anti-degenerative properties are not dependent on the unique features of transgenic mouse models.

Effects of LM11A-31 in APP-L/S mice have also been evaluated using mouse PET imaging applying the GE-180 TSPO protein ligand as a marker for microglial activation. GE-180 is a third generation TPSO PET ligand that achieves substantially higher binding affinity and specificity than the first generation PK11195-type ligands and has raised considerable promise as a PET modality for monitoring microglial activation in AD and other neurodegenerative diseases[7, 8]. Through a collaboration with the Stanford Department of Radiology (Drs. Michelle James and Sam Gambir), APP-L/S mice were treated once daily for three months with LM11A-31 as applied for the above morphological and behavioral studies. As shown here in Figure 5 (in vivo mouse PET, 7-9 mice/group ± SE; *p < 0.05), GF-180 detects significantly increased signal in APP-L/S mice compared to wild type and this increase was blocked by LM11A-31. Inhibition of microglial activation in these mice was confirmed using CD68 IHC (marker of activated microglia) (data not shown). These studies were presented at the Society for Neuroscience 2014 meeting (James et al., SNS 2014 Abstracts on Line, featured in ALZFORUM News, December 10, 2014, on line) and a manuscript is in preparation. While GE-180 PET will likely not be available for our proposed IIa studies, this mouse work confirms through yet another modality and research team that LM11A-31 effects microglial activation in this mouse model and if GE-180 becomes validated for human trials, it could be quite useful as part of a subsequent phase 2b/3 LM11A-31 trial.

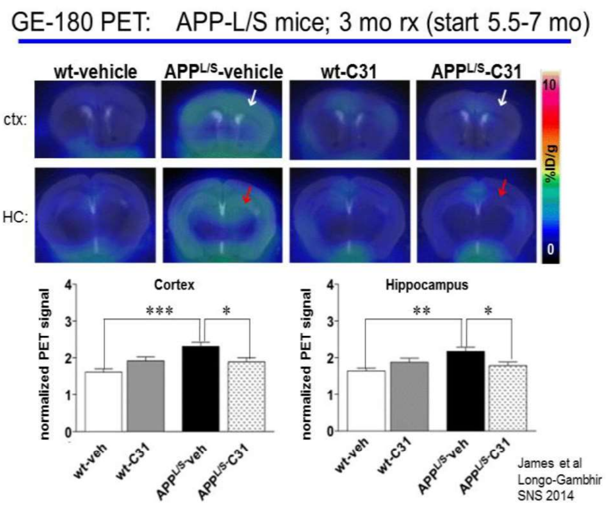

Figure 5: PET Scan Analysis

In dose-response studies in APP-L/S mice[1] a once daily dose of 50 mg/kg (38 mg/kg free base form) led to a maximum therapeutic effect on neurite degeneration; a dose of 25 mg/kg (19 mg/kg free base form) led to 80% of the maximum effect and a dose of 5 mg/kg (3.8 mg/kg free base form) resulted in a small trend for a protective effect that did not reach significance. Remarkably, a study led by Xie and Meeker at UNC in collaboration with Dr. Longo (Xie et al, Society for Neuroscience, Abstracts on Line 2013) in reversing basal forebrain cholinergic neuron atrophy in aged wild type mice gave an almost identical dose response. The human equivalent dose, based on the FDA-recommended BSA calculations, of 38 mg/kg is 104 mg in a 70 kg human. Thus phase 1 doses of 900 mg/day (free base equivalent) reached in the SAD studies and 600 mg/day reached in the MAD studies, tested doses > 5-fold above the equivalent doses (as calculated by standard BSA conversion) in mice giving maximal effect on inhibiting degeneration. Phase 1 studies indicated that drug exposure in humans was some 30-40% greater for a given dose than that expected from BSA-based mouse to human conversion; therefore, human exposures in our phase 1 trial were > 7-8-fold higher than those achieving maximal therapeutic effect in mice. Hence, we expect that the proposed phase IIa doses of 400 mg (200 mg bid) and 800 mg (400 mg bid) per day will be well tolerated and result in drug exposures in AD patients > 5-fold that achieving maximal therapeutic levels in AD mouse models. LM11A-31 has been applied in two forms using HCl or bisulfate counter ions

(LM11A-31-BHS) which exhibit similar LM11A-31 pharmacokinetics but with the BHS salt form amendable to human application. Preclinical safety and phase 1 trials were conducted with LM11A-31-BHS.

9.1 General Considerations

A phase IIa, multi-center, randomized, placebo-controlled, parallel group clinical study with a 26-week double-blind treatment duration and three treatment arms with two consisting of two doses of LM11A-31 (200 mg/bid and 400 mg/bid of free base with adaptive dose design) and one comprising placebo.

The primary goal of the present phase IIa clinical trial is to evaluate the safety and tolerability of two doses of LM11A-31-BHS administered orally twice daily for 26 weeks versus matched placebo. Safety monitoring will include the full extent of phase 2 clinical and laboratory testing.

Exploratory and efficacy endpoints will include: regional brain glucose metabolism via FDG-PET; CSF biomarkers (tau, p-tau, Aβ40, Aβ42, AChE activity) and cognitive/behavioral measures including the NTB consisting of Digit Span Test, Category Fluency Test, the Digit Symbol Substitution Test (DSST) and the Controlled Oral Word Association Test (COWAT). In addition, the ADAS-Cog 13 items, the spatial orientation and learning with Amunet, the Digit Symbol Substitution Test, the Geriatric Depression Scale (GDS) and the Clinical Global Impression – Improvement test (CGI-I) will be performed. Plasma and CSF drug levels will also be measured.

As outlined in the FDA guidelines for CNS affecting drugs, the Columbia-Suicide Severity Rating Scale (C-SSRS) will also be conducted within the course of the trial. LM11A-31 outcomes in mouse trials are particularly potentially related to: human FDG-PET (improved synaptic function); p-tau (inhibition of tau phosphorylation); and multiple cognitive effects including (Amunet) spatial orientation (effects on the MWM-DTMP and 4 other mouse behavioral studies). We do not expect an effect on CSF Aβ but this measurement will serve diagnostic purposes.

9.2 Appropriateness of measurements

In this study, assessments and scales are included which have been well validated for evaluating the clinical efficacy of marketed and novel compounds for treatment of AD. The tests allow assessment of change in several of the central functions that are impaired in AD. They are also suitable for measuring the time course of disease and the time course of drugs action.
A Neurological Testing Battery (NTB) consisting of Digit Span Test, Category Fluency Test, the Digit Symbol Substitution test and the COWAT will be used. In addition, the ADAS-Cog 13 items, CGI and the AMUNET will be applied.
MMSE measures key aspects of cognitive performance such as memory and executive function.
The GDS is a screening test for depressive symptoms in the elderly. This test is ideal for evaluating the clinical severity or absence of depression.
The battery of tests and scales reflect state of the art for drug testing in AD.

9.3 Treatment Groups

While patients are unlikely to benefit from placebo treatment, a placebo group is required in order to differentiate any investigational drug effect from any improvement that may occur solely due to the close care and medical oversight given to patients under trial conditions. It is well-known that placebo effects do occur as a result of intensive support of patients during a clinical trial and in an AD specific trial, positive cognitive performances could occur within the first 2-3 months as a result. This finding is supported in publications describing clinical trials in AD.

In addition, minimal restrictions on concomitant medication will be implemented (see Inclusion and Exclusion Criteria – Sections 9.7.2 ; Prohibited Concomitant Medication – Section 11.8)

9.4 Choice of study medication dosage

Patients will be randomly assigned to LM11A-31-BHS 200 mg bid, 400mg bid or placebo. Results of Phase I showed that LM11A-31 is generally well tolerated and safe. The phase I clinical trial was a randomized, double-blind, placebo-controlled, single ascending dose (SAD) and multiple ascending dose (MAD) study in healthy young male and elderly male and female subjects. The rational of the 26-week duration is to test LM11A-31 for a maximum duration feasible for a first-time IIa safety and exploratory endpoint trial that allows optimal detection of effects on clinical and biomarker endpoints and any safety issues.

Additional information can be found within the Investigator's Brochure.

9.5 Duration of the treatment

A 26-week treatment period has been chosen, as fewer months of treatment are often insufficient to differentiate significant treatment effects from placebo effects in this population of AD patients at this recruitment level. Conversely, the 26-week period also ensures that patients receiving placebo are not placed at significant risk or disadvantage due to their participation in this study. Since the expected disease progression during the natural course of AD in this population of patients is estimated to be a 3-6 points deterioration within six months (using ADAS-Cog scale), subsequent benefit from either eventual LM11A-31-BHS treatment, or from an alternate approved drug, is possible. And, there is no medication currently approved or available to modify AD progression at this time.

The study period was chosen in accordance with recommendations of the CHMP guideline on medicinal products for treatment of AD (CPMP/EWP/553/95 Rev.1) suggesting that controlled clinical trials in AD should last at least six months. The 26-week duration of the double-blind treatment is therefore used to maximize the likelihood of detecting a difference between drug and placebo and to gain clinically relevant information for any subsequent evaluation of LM11A-31-BHS in confirmatory treatment trials.

9.6 Randomization

Randomization introduces an element of chance to the assignment of treatments. This also tends to produce treatment groups which are similar with respect to distributions of prognostic factors. In conjunction with blinding, randomization helps to avoid possible bias in the selection and allocation of patients. Eligible patients will therefore be randomized to the treatment groups in a ratio of 1:1:1, 200 mg bid LM11A-31-BHS (80 patients), 400 mg bid LM11A-31-BHS (80 patients) and placebo (80 patients), following recruitment.

Data Magik Ltd (DML) will produce the SAS program code to output the randomization list containing patient randomization numbers and treatment codes. This SAS program code will be transferred to an independent unblinded statistician who will output the live randomization list on behalf of DML and upload it to the Kapture IWRS system. ACE Pharmaceuticals will be packaging and labelling all medication for this study, and distributing the medication to sites when requested through the DML Kapture EDC system. The randomization process will be managed and controlled centrally by the Kapture IWRS. Thus, the patients will be randomized in the IWRS system by the site investigators (Kapture IWRS) and allocated a patient randomization number and a medication kit pertaining to a treatment arm according to the list supplied by ACE Pharmaceuticals to Data Magik (unblinded DML application developer). Full details of these procedures involved are given

in the study-specific DML IWRS User Requirement Specification, and ACE- Data Magik Medication-IWRS Data Transfer Specifications.

Each patient participating in the trial will receive medication in packages labelled with their specific kit randomization and Patient ID. Due to anticipated expiration dating issues, study centers may be supplied with individual patient medication kits on an ongoing basis in order to ensure that the investigational products are fully qualified for use. Patients will receive their individual medication kits in alignment with their study visits and no additional study visits are envisioned for the purpose of re-supply of investigational product.

9.7 Selection of Study Population

A total of 240 male and female patients will be enrolled in the phase IIa clinical trial. All patients must be capable of giving informed consent and have diagnosed mild to moderate probable Alzheimer's disease according to McKhann et al. (2011)[9].

The result of a MRI or CT scan performed within the six months prior to baseline should be consistent with the diagnosis of mild to moderate probable AD, corroborating the clinical diagnosis and excluding other potential causes of dementia, especially cerebrovascular lesions (see exclusion criteria, number 3). Patients who fulfil all of the inclusion criteria and none of the exclusion criteria listed below will be eligible for enrollment after a screening period of eight weeks (56 days).

Based on preclinical studies in several mouse models of Alzheimer's disease we expect that subjects receiving verum could have a reasonable chance of benefiting from the test compound. Currently there are no treatments available that have been demonstrated to modify the neurodegenerative process occurring in Alzheimer's disease. When administered to Alzheimer mouse models once per day for durations ranging from 1-3 months by oral gavage, LM11A-31-BHS has been shown to slow as well as reverse degeneration of neuronal connections (neurites) in the basal forebrain and cerebral cortex[1, 3, 6]. In these same studies, and consistent with the ability of LM11A-31-BHS to inhibit degenerative signaling induced by amyloid-, the drug inhibited the accumulation of pathological forms of the tau protein. Unpublished but publicly presented studies (Longo) demonstrate that LM11A-31-BHS inhibits the accumulation of oligomeric forms of tau in these same mouse model studies. In a well-established model of synaptic failure induced by amyloid, LM11A-31-BHS normalized synaptic function after 30 minutes of treatment[5]. Thus, within the 6-month treatment period, it is very possible that subjects might experience some degree of improvement in synaptic and hence cognitive function.

It is important to note that the mechanism of action of LM11A-31-BHS is not limited to Alzheimer's pathology. LM11A-31-BHS inhibits degenerative signaling of the p75 neurotrophin receptor and in doing so inhibits a wide spectrum of degenerative processes within neurons, including those related to aging. In unpublished work presented at the Society for Neuroscience (Longo), LM11A-31-BHS, administered over 1-4 month periods, was shown to reverse atrophy and neurite degeneration of basal forebrain cholinergic neurons. Such atrophy is thought to contribute to age-related cognitive loss and the loss of response to acetylcholinesterase medication. This age-related beneficial effect provides an additional mechanism through which verum might be expected to promote positive cognitive effects in test subjects.

Subjects will be monitored with multiple cognitive assessments, as well as with FDG-PET for the purpose of detecting improvements in synaptic and cognitive function. While the proposed study is not designed to treat and monitor subjects over a long time period, it is also possible that treatment with LM11A-31-BHS over the proposed 6-month period, might slow overall long term progression. It has been clarified in the revised submission that the mechanism of action of LM11A-31-BHS, while highly effective in processes considered to underlie degeneration and synaptic impairment

in Alzheimer's disease, is not entirely specific to Alzheimer's disease. LM11A-31-BHS inhibits degenerative signaling of the p75 neurotrophin receptor and in doing so inhibits a wide spectrum of fundamental signaling networks within neurons, including mechanisms related to excess calcium influx, mitochondrial impairment, calpain activation, excess rhoA activation and degenerative stress kinase activation. Accordingly, LM11A-31-BHS has been found to be effective in pre-clinical models of traumatic brain injury[10], spinal cord injury[11], cisplatin-induced neuropathy[12] and HIV-induced dementia[13]. In unpublished work presented at the Society for Neuroscience (Longo), LM11A-31-BHS has been shown to reverse atrophy and neurite degeneration of basal forebrain cholinergic neurons in wild type aged mice in addition to its ability to inhibit degeneration of these neurons in Alzheimer mouse models. These neurons undergo degeneration in Lewy Body and vascular dementia, the two most common other causes of dementia after Alzheimer's disease. This broad spectrum of effects indicate that LM11A-31-BHS might well be effective in multiple forms of dementia beyond Alzheimer's disease. Given these mechanism of actions, LM11A-31-BHS is much more likely to be effective in non-Alzheimer's forms of dementia than other therapies currently in trials, including those targeting amyloid levels, amyloid production and accumulation of toxic forms of tau.

9.7.1 Inclusion criteria

1. Men and women (non-childbearing potential) with a diagnosis of Alzheimer's disease according to McKhann (2011) criteria
2. Austria, Germany, Spain, Sweden: Age 50 - 85 years

   Czech Republic: Age 50-80 years
3. MRI or CT assessment within six months before baseline, corroborating the clinical diagnosis of AD and excluding other potential causes of dementia, especially cerebrovascular lesions (see exclusion criteria, number 3)
4. CSF AD specific biomarker profile; positive, defined as CSF Aβ42 <550 ng l−1 or a Aβ 40/42 ratio <0,89.
5. Mild to moderate stage of Alzheimer's disease according to MMSE ≥18 and ≤26
6. Absence of major depressive disease according to GDS of < 5
7. Modified Hachinski Ischemic Scale ≤4
8. Formal education for eight or more years
9. Previous decline in cognition for more than six months as documented in patient medical records
10. Other Countries: A caregiver available and living in the same household or interacting with the patient for a sufficient time each week and available if necessary to assure administration of drug

    Czech Republic: "Patients with moderate Alzheimer's Disease must have a caregiver available and living in the same household. Patients with mild AD must have a caregiver either living in the same household or a caregiver interacting with the patient, providing personal care for the patient during at least 10 hours per week and available if necessary to assure administration of drug"
11. Patients living at home or nursing home setting without continuous nursing care
12. General health status acceptable for a participation in a 6-month clinical trial
13. Ability to swallow capsules
14. Stable pharmacological treatment of any other chronic condition for at least one month prior to screening
15. Stable treatment with one of the acetylcholinesterase inhibitors donepezil (Aricept ®), galantamine (Razadyne®), or rivastigmine (Exelon) or the partial NMDA receptor antagonist with memantine (Namenda®) at least 3-months before baseline Visit or Combination of both treatments mentioned above

16. No regular intake of prohibited medications as noted in Section 11.8.
17. Only applicable for Germany and Czech Republic:
    Signed informed consent by the patient, examined and verified to be mentally capable by an independent physician/ neurologist, prior to the initiation of any study specific procedure. Signed consent of the caregiver (see inclusion criteria 10)

All inclusion criteria and none of the exclusion criteria listed below have to be achieved to include a patient.

9.7.2 Exclusion criteria

1. Failure to perform screening or baseline examinations
2. Hospitalization or change of chronic concomitant medication one month prior to screening or during screening period
3. Clinical, laboratory or neuro-imaging findings consistent with:
   - Other primary degenerative dementia, (dementia with Lewy bodies, fronto-temporal dementia, Huntington's disease, Creutzfeldt-Jakob Disease, Down's syndrome, etc.)
   - Other neurodegenerative condition (Parkinson's disease, amyotrophic lateral sclerosis, etc.)
   - Cerebrovascular disease (major infarct, one strategic or multiple lacunar infarcts, extensive white matter lesions > one quarter of the total white matter)
   - Other central nervous system diseases (severe head trauma, tumors, subdural hematoma or other space occupying processes, etc.)
   - Seizure disorder
   - Other infectious, metabolic or systemic diseases affecting central nervous system (syphilis, present hypothyroidism, present vitamin B12 or folate deficiency, serum electrolytes out of normal range, juvenile onset diabetes mellitus, etc.)
4. A current DSM-IV diagnosis of active major depression, schizophrenia or bipolar disorder
5. Clinically significant, advanced or unstable disease that may interfere with primary or secondary variable evaluations, and which may bias the assessment of the clinical or mental status of the patient or put the patient at special risk, such as:
   - chronic liver disease, liver function test abnormalities or other signs of hepatic insufficiency (ALT, AST, Gamma GT, alkaline phosphatase > 2.5 ULN)
   - Respiratory insufficiency
   - Renal insufficiency (serum creatinine >2mg/dl) or creatinine clearance ≤ 30 mL/min according to Cockcroft-Gault formula). In case of creatinine clearance ≤30 mL/min, an alternative verification of the renal function must be completed using Cystatin C analysis. In case of normal level of Cystatin C, the patient can be included
   - Heart disease (myocardial infarction, unstable angina, heart failure, Cardiomyopathy within six months before screening)
   - Bradycardia (heart beat <50/min.) or tachycardia (heart beat >95/min.)
   - <u>For Austria, Germany, Spain and Sweden</u>: Hypertension (>180/95 or hypotension (<90/60) requiring treatment with more than three drugs
   - <u>For Czech Republic</u>: Hypertension (>160/95) or hypotension (<90/60) requiring treatment with more than three drugs
   - AV block (type II / Mobitz II and type III), congenital long QT syndrome, sinus node dysfunction or prolonged QTcB-interval (males >450 and females >470 msec)
   - Uncontrolled diabetes defined by HbA1c >8.5
   - Malignant tumors within the last five years except skin malignancies (other than melanoma) or indolent prostate cancer

- Metastases

6. Disability that may prevent the patient from completing all study requirements (*e.g*. blindness, deafness, severe language difficulty, etc.)
7. Women who are fertile and of childbearing potential
8. Chronic daily drug intake of ≥ 14 days or expected for ≥ 14 days:
    - benzodiazepines (except lorazepam ≤ 1mg for sleeping disorders only), neuroleptics or major sedatives
    - Antiepileptics
    - Centrally active anti-hypertensive drugs (clonidine, l-methyl DOPA, guanidine, guanfacine, etc.)
    - Opioid containing analgesics
9. Nootropic drugs (except Ginkgo Biloba)
10. Austria, Germany, Spain and Sweden: Suspected or known drug or alcohol abuse, *i.e*. more than approximately 60 g alcohol (approximately 1 liter of beer or 500 ml of wine) per day, indicated by elevated MCV significantly above normal value at screening

    Czech Republic: Suspected or known drug or alcohol abuse, i.e. more than approximately 20 g alcohol per day for females (500 ml of beer or 250 ml of wine) and 30g alcohol per day for males (approximately 750 ml of beer or 375 ml of wine) indicated by elevated MCV significantly above normal value at screening
11. Suspected or known allergy to any components of the study treatments
12. Enrollment in another investigational study or intake of investigational drug within the previous three months
13. Any condition, which, in the opinion of the investigator, makes the patient unsuitable for inclusion
14. If patient is in any way dependent on the sponsor or the principal investigator or if the patient is accommodated in an establishment on judicial or administrative order.

9.8 Removal of patients from treatment or assessment

In accordance with the informed consent and the Declaration of Helsinki, each patient may discontinue the study at any time for any reason without any penalty or loss of benefits to which the patient is otherwise entitled. Reason(s) for a premature termination should be documented in the CRF. The final examination should be performed in such cases for safety reasons.

Once randomized, every possible effort will be made to ensure that all patients remain in the study for the full 26-week duration of the study. This will ensure that investigator bias does not influence the treatment outcome.

Patients may be withdrawn from the study by the investigator at any time for any of the following reasons:

- When a patient is generally not compliant with study procedures
- When there are doubts that a patient is generally complying with intake of study medication and use of allowed concomitant medication (see Section 11.10 regarding dose interruption or non-compliance with dosing schedule)
- When a patient moves to a new location too far away from the study center, that further participation in the study becomes questionable
- If the caregiver is no longer available and no substitute is taking over

Absolute withdrawal criteria for this study are:

- Safety reasons in the best interests of the patient

- Patient withdraws at his/her request
- The patient is lost to follow-up

In addition, the following ECG changes would trigger withdrawal of a patient in accordance with ICH topic E14: "Clinical Evaluation of QT/QTc Interval Prolongation and Proarrhythmic Potential for Non-Antiarrhythmic Drugs (CHMP/ICH/2/04)".

- QTcB changes > 60 msec relative to baseline
- Absolute QTcB (or at low heart rates, uncorrected QT > 500 msec)
- QTcB dispersion > 100 msec
- Change in QTcB dispersion across the 12 leads of more than 100% compared to baseline

Upon withdrawal from the study, each patient must be followed up for a final examination including all Visit 5 measurements. Patients discontinuing the study must be fully documented up to the time of discontinuation.

Dropouts and withdrawals will not be replaced. Patients who are withdrawn from the study due to AEs will be treated according to standard clinical strategies and will be followed up until they are resolved or stabilized, or until a plausible explanation for the cause of the event has been found. All pertinent information concerning the outcome of the AE will be documented in the case report form.

In emergency situations, when the identity of the study drug must be made known to the investigator in order to provide appropriate treatment, the investigator can unblind a patient anytime via the IWRS. If the blind has been broken, the investigator must justify why the blind was broken in the IWRS. Automated notification will be sent to the study safety officer (SSO) in case the investigator unblinds a patient.

Conditions developing during the course of the study listed in the exclusion criteria as well as protocol deviations will not necessarily lead to the patient's withdrawal but must be properly documented.

9.9 Premature termination or suspension of the study

The clinical trial can be prematurely terminated or suspended by the Sponsor after re-assessing the risks of the study.

The study must be prematurely terminated for the following reasons:

- Occurrence of unexpected serious adverse reactions jeopardizing the health of the patient population, as assessed by the DSMB
- New scientific data on the investigational product do not justify continuation of the clinical study
- Serious and/or persistent non-adherence to the protocol, ICH GCP and/or applicable regulatory requirements by an Investigator/institution

Furthermore, the Sponsor has the right to terminate the study for any reason at any time. If the study is prematurely terminated or suspended for any reason, the investigator must inform the patients and assure appropriate follow-up for the patients. The Sponsor should promptly inform the investigators/institutions, and the Regulatory Authority(-ies) of the termination or suspension and the reason(s) for them, as specified by the applicable regulatory requirement(s). The IEC/IRB should also be informed promptly and provided the reason(s) for the termination or suspension by the Sponsor or by the investigator/institution, as specified by the applicable regulatory

requirement(s). The investigator is responsible for informing all patients of a premature termination or suspension of the trial.

10 STUDY OBJECTIVES

Primary/Safety Objectives

Safety will be assessed through adverse event reporting and a standard range of patient physical evaluations including a suicide severity rating scale (C-SSRS). Primary goals are

- to investigate the safety and tolerability of 200mg bid and 400mg bid doses of LM11A-31-BHS (free base) administered for a period of 26 weeks in comparison to placebo.

Secondary/Exploratory

PK parameters for LM11A-31 and its aminoethyl morpholine metabolite in plasma will be measured. LM11A-31 and its aminoethyl morpholine metabolite will also be measured in selected CSF samples that are otherwise obtained for biomarker purposes. Furthermore, to investigate the supporting efficacy and pharmacodynamic effects, regional brain glucose metabolism (18F-FDG-PET) and CSF biomarker assessment (tau, p-tau, Aβ40, Aβ42, AChE activity) will be performed at screening and after LM11A-31-BHS administration for a period of 26 weeks.

Exploratory efficacy will be assessed using a composite of the specific cognitive tests performed (the NTB composite standardized Z score). Additional efficacy measurements will be performed through a carefully selected range assessments including:

- NTB:
  - Digit Span Test
  - Category Fluency Test
  - COWAT
  - Digit Symbol Substitution Test (DSST)
- ADAS-cog 13 items
- Spatial Orientation and Learning (Amunet)
- Geriatric Depression Scale (GDS)
- Clinical Global Impression Scale – Severity (CGI-S) and -Improvement (CGI-I)

To ensure sensitivity and reliability of exploratory efficacy assessments, the same rater for a given instrument should always assess the status of a given patient at all visits. All raters should have completed training or confirmed their training for particular psychometric test.

The efficacy and diagnostic assessments will be performed at the times indicated in the study schedule (Flow Chart 13.1) and are described below.

If patients discontinue treatment prematurely during the double-blind treatment phase, all of the assessments should be performed as scheduled for Visit 5. If a patient discontinues treatment prior to Visit 3 (Day 28), then this patient will be included in the intent-to-treat population based upon the unscheduled, termination testing.

10.1 Primary/ Safety Variables

All adverse events will be evaluated and documented. Values from laboratory evaluations and vital signs will be recorded at all study visits. Clinically relevant changes in laboratory values will be recorded on the eCRF by the site investigator and all clinically relevant changes from baseline and

reference values will be identified and forwarded to the study safety officer for evaluation. The investigator will evaluate whether a laboratory value that is out of the pre-determined range is considered as clinically significant or not clinically significant. Clinically relevant changes will be considered as AEs (see 14.1, Safety reporting).

10.1.1 Vital Signs

Vital signs will be performed at every visit. Vital signs should be measured before blood is drawn for laboratory tests. Measurements of systolic and diastolic blood pressure, heart rate and respiratory rate should be made after the patient has been in sitting position for 5 minutes. Body temperature, height (only at visits 1, 5), BMI (only at Visits 1, 5) and weight are subsequently measured.

10.1.2 Blood pressure (BP - systolic and diastolic)

The Blood pressure will be measured by means of a standard manual or an automatic blood pressure measuring device. Nevertheless, the same method should be used in individual patients during the entire study period. The same arm will be used for each measurement of blood pressure.

10.1.3 12-Lead ECG

A 12-lead ECG will be performed in supine posture at Visits 1, 3, 4, 5 and early discontinuation visit. These evaluations will either be performed at the investigational site or at alternate local site if the required equipment is not available. The first evaluation of the ECG will be done and/or reviewed by a listed principal or sub-investigator to assess the patient's status and determine his/her ability to participate safely in the study. A printout of the ECG will be kept in the Patient File, signed and dated by the responsible investigator. Abnormalities will be documented in the CRF.

QT will be corrected using Bazett's formula (QTc = QT / square root of RR).

Reference ranges for 12-lead ECG parameters are:

Heart rate:	40-90 bpm
PR:	120-210 msec
QRS:	70-120 msec
QTc:	350-430 msec (350 - 450 msec for females)

In the event of QT interval prolongation (i.e., QTc > 450 msec in males or >470 msec in females) at screening, the patient cannot be enrolled into the study.

A second ECG copy will be sent to a central ECG reader for manual over-read and measurement of QT/QTc.

In addition, the following ECG changes would trigger withdrawal of a patient in accordance with ICH topic E14.

- QTcB changes > 60 msec relative to baseline
- Absolute QTcB (or at low heart rates, uncorrected QT > 500 msec)
- QTcB dispersion > 100 msec
- Change in QTcB dispersion across the 12 leads of more than 100% compared to baseline

All ECGs will be sent via e-mail to the central reader Dr. Föchterle (office@foechterle.at). The central reader will evaluate the ECG results and send back his analysis and additional explanations, if possible concerns regarding the patient's safety can be seen in the ECG results.

10.1.4 MRI

Central rating of medial temporal lobe according to Scheltens Score (Central rating should include all other MRI parameters). The right and left medial temporal structures will be rated separately, and an overall estimate will be created using the average of the two ratings.

10.1.5 Laboratory

Blood specimen and urine samples will be analyzed for the following items.

10.1.5.1 Hematology:

Erythrocytes, hemoglobin, hematocrit, MCH, MCV, MCHC, reticulocytes, leucocytes, neutrophils, eosinophils, basophils, lymphocytes, monocytes and thrombocytes.

10.1.5.2 Blood Biochemistry:

Total bilirubin, ALT, AST, LDH, alkaline phosphatase, gamma-GT, creatinine, Blood Urea Nitrogen, uric acid, CK, sodium, potassium, inorganic phosphate, calcium, chloride, magnesium, albumin, total cholesterol, triglycerides, glucose, total protein, INR (prothrombin time), homocysteine, creatinine clearance and cystatin C.

10.1.5.3 Urinalysis:

Glucose, pH, albumin, ketones, red and white blood cells, specific gravity, nitrites, urobilinogen, bilirubin.

10.1.5.4 Additional Lab for Screening:

Coagulation variables (aPTT, Fibrinogen, D-Dimer, INR) are measured at each visit. Anticoagulated patients are excluded from the study, yet for safety reasons these data are collected.).

10.1.6 C-SSRS

Prospective suicidal ideation and behavior assessments will be conducted with the Columbia Suicide Severity Rating Sale (C-SSRS) test as stipulated for clinical studies on CNS active drugs in the FDA guidance for industry "Suicidal Ideation and Behavior: Prospective Assessment of Occurrence in Clinical Trials". The C-SSRS captures the occurrence, severity, and frequency of suicide-related thoughts and behaviors during the assessment period. The scale includes suggested questions to solicit the type of information needed to determine if a suicide-related thought or behavior occurred. The C-SSRS will be conducted by trained personnel at the clinical facility at every visit.

10.2 Efficacy/ Exploratory Endpoints

10.2.1 Regional Brain Glucose Metabolism (^{18}F-FDG-PET)

At baseline and final visit the specific ^{18}F FDG-PET scan for early detection of AD[14] will be conducted. Quantitative and qualitative estimates of the cerebral glucose rate may be done by using FDG-PET as evident metabolic reduction is already present in patients at early stages of Alzheimer' s disease [15, 16].

10.2.2 CSF Biomarker

CSF samples will be obtained at screening visit and final visit via lumbar puncture to determine levels of tau, p-tau, Aβ40, Aβ42 and AChE activity. Aliquots of CSF will also be made available for emerging CSF biomarker methods such as measurement of tau oligomers and for measurement of drug level in selected patients based on time period between last dose of drug and CSF sampling.

10.2.3 Neurological Testing battery (NTB)

10.2.3.1 Digit Span

Study participants are read sequences of numbers are and in the first phase of the test required to repeat them as heard (Digits Forward). In the second phase of the test, Digits Backwards, study participants are required to repeat the sequence in the reverse order. Two trials are administered for each sequence length and 1-point is awarded for each sequence correctly repeated.

Testing will be performed at Visits 2, 4 and 5, as well as at the early discontinuation visit, if applicable.

Rater: Trained psychologist or physician

10.2.3.2 Category Fluency Test

In this test, study participants are required to generate words from a specific category (usually animals) in one minute. Performance across the minute is scored according to acceptable rules to yield the total number of correct responses. This test measures working memory and other aspects of executive function, including planning, strategy and aspects of language and especially fluency.

Testing will be performed at Visits 2, 4 and 5, as well as at the early discontinuation visit, if applicable.

Rater: Trained psychologist or physician

10.2.3.3 Controlled Oral Word Association test (COWAT)

The COWAT measures a person's ability to make verbal associations to specified letters (i.e., C, F, and L), evaluates the spontaneous production of words beginning with a given letter and is able to detect changes in word association fluency often found with various disorders.

COWAT testing will be performed at Visits 2, 4 and 5, as well as at the early discontinuation visit, if applicable.

Rater: Trained psychologist or physician

10.2.3.4 Digit Symbol Substitution Test

In the Digit Symbol Substitution Test (DSST), the patient is required to match symbols with their corresponding digit. The test consists of 9 digit symbols, which are to be matched with their corresponding numerical digit. The patients will have limited amount of time to enter the correct symbol for each digit.

DSST will be performed at Visits 2, 4 and 5, as well as at the early discontinuation visit, if applicable.

Rater: Trained psychologist or physician

10.2.4 Alzheimer's Disease assessment scale – 13items (ADAS-Cog 13 items)

Alzheimer's Disease assessment scale – 13 items (total score = 85) is used as a parameter for efficacy. ADAS-cog is a psychometric instrument designed to evaluate the severity of cognitive and non-cognitive behavioral dysfunctions characteristic of people with AD. The cognitive portion assesses memory, language and praxis functions. Most items are rated on a scale of 0 to 5, where 0 indicates no impairment and 1 to 5 indicates very mild (1), mild (2), moderate (3), moderately severe (4) or severe impairment (5) respectively. Other items are rated on the presence or absence of a characteristic number of errors or severity of errors. The total scores from the ADAS-cog sub-scales range from 0 (no impairment) to 85 (errors in all sub-tests).

A positive (i.e. increasing) change of the score indicates cognitive worsening. The spontaneous decline in performance in patients with AD is associated with about an 8 to 9-point increase in ADAS-cog score over one year.

ADAS-Cog will be assessed at Visits 2, 4 and 5, as well as at the early discontinuation visit, if applicable.

Rater: Neurologist, psychologist or other personal (listed as either Principal Investigator or Sub-Investigator) with documented training in psychometric rating.

10.2.5 Spatial Navigation Testing with Amunet

Impaired orientation in space is a frequently reported symptom in AD patients[17, 18]. *Hort et al* concluded that spatial navigation impairment occurs early in the development of AD and can be used for monitoring of the disease progression or for evaluation of presymptomatic AD[19].

The two modes of spatial navigation include egocentric and allocentric navigation. Egocentric navigation uses information about distances and angles from the subject positions processing proprioreceptive information, whilst allocentric navigation is hippocampus dependent and uses a flexible representation of a distal landmarks ensemble independent of actual subject positions[20]. The parietal cortex including precuneus[21], and especially the hippocampus, is involved in spatial navigation performance[22]. Impairments are particularly found in patients suffering from memory deficits related to the hippocampal area – correlating to prodromal AD pathologic findings[23], presumably a signal for preclinical AD[19].

Memory paradigms used with human study participants suffering from AD typically feature tests of episodic verbal memory, paired associative learning or visual recognition memory. These tasks are very different to the memory paradigms in rodents, where the Morris water maze (MWM) is employed in preclinical studies for the development of new medicinal products for AD[24].

Amunet, the computer-based simulation of MWM, tests the two basic types of navigation: world-centered – allocentric (hippocampus-dependent) and body-centered – egocentric (parietal cortex-dependent). Both of these paradigms are controlled by the structures involved in early Alzheimer's disease pathology. Allocentric navigation is independent of an individual's position and distal cues are used for navigation, while egocentric navigation depends on an individual's position and the start position is used for navigation[24]. Amunet computer simulation is a map view of the arena projected on computer screen where the participant uses a touch screen to identify the target position. The arena in the computerized version of the MWM was shown as a large white circle with the start position (medium-sized red circle) and 2 orientation cues (yellow and green lines) on its perimeter. A small red circle inside the arena represents the goal[24].

Amunet will be conducted at Visits 2, 4 and 5, as well as at the early discontinuation visit, if applicable.

Rater: Trained psychologist or physician

10.2.6 Clinical Global Impression Scale

The Clinical Global Impressions Scale - Severity (CGI-S) and - Improvement (CGI-I) will be used by the investigator to evaluate (at screening visit) and compare the patient's condition to the previous study visits (CGI-I). The screening visit (Visit1) will serve as a basis for the assessment. The subjective categorical values of the CGI-I[25] are as follows:

1=very much improved since the initiation of treatment;
2=much improved;
3=minimally improved;

4=no change from baseline (the initiation of treatment);
5=minimally worse;
6= much worse;
7=very much worse since the initiation of treatment.

The CGI-S will be assessed at Visit 1, and CGI-I for evaluation of improvement of subject's condition at Visit 4, 5 and early discontinuation visit, if applicable.

Rater: Neurologist, psychologist or other personal (listed as either Principal Investigator or Sub-Investigator) with documented training in psychometric rating.

10.2.7 Geriatric Depression Scale (GDS)

The Geriatric Depression Scale (GDS) is a useful validated screening tool to facilitate assessment of depression in older adults especially when baseline measurements are compared to subsequent scores. The GDS Short Form consists of 15 Items and takes about 5 to 7 minutes to be completed. Of the 15 items 10 indicate the presence of depression when answered positively, while the rest indicate depression when answered negatively. A score of 0 to 4 is normal, depending on age, education, and complaints; a score of 5 to 8 indicates a mild depression, 9 to 11 reflects a moderate depression, 12-15 a severe depression. Evaluation of the GDS will be performed at screening and only patients with GDS score<5 will be enrolled in the study.

The GDS will be assessed at Visits 1, 2, 3, 4, 5 and early discontinuation visit, if applicable.

Rater: Trained psychologist or physician

10.3 Pharmacokinetics

Plasma Samples will be collected to investigate the pharmacokinetics PK parameters for LM11A-31 and its aminoethyl morpholine metabolite. The time between PK sampling and last intake of study drug will be recorded. Levels of LM11A-31 and its aminoethyl morpholine metabolite will also be measured in selected CSF samples that are otherwise obtained for studies of CSF biomarkers.

10.4 Diagnostic Variables

10.4.1 Laboratory

Folic acid and vitamin B12 will be analyzed to define eligible patients (see inclusion/exclusion criteria). TSH, free T4 and TPPA test for syphilis will be analyzed at screening to define eligible patients.

10.4.2 ApoE genotyping

At screening, DNA samples will be collected for the assessment of the ApoE ε4 allele status. Therefore, 2.7 ml blood will be collected in an EDTA tube. The samples will be send to the central laboratory (Labor Dr. Spranger und Partner; D-85051 Ingolstadt, Lindberghstrasse 9-13; Germany) for the analysis by using PCR technique. DNA samples will be stored until the end of the trial and destroyed thereafter. Subjects will be blinded to the ApoE genotyping results within the scope of the study.

The results will be forwarded to the sponsor at the end of the study. But, consideration will be given to patient confidentiality concerning the distribution and availability of their genetic data (EMEA, ICH Topic E15 Definitions for genomic biomarkers, pharmacogenomics, pharmacogenetics, genomic data and sample coding categories, 2008).

10.4.3 CSF AD specific lab details

A CSF AD specific biomarker profile is defined positive if CSF Aβ42 <550 ng l^{-1} or a Aβ40/42 ratio <0,89.

The results of all patients will be forwarded to the sponsor at the end of the study.

10.4.4 Mini-Mental State Examination (MMSE)

The MMSE[26] is a frequently used screening instrument for AD drug studies. The instrument provides for evaluation of orientation, memory, attention, concentration, naming, repetition, comprehension, ability to create a sentence and to copy two intersecting polygons. This examination is frequently used by physicians in the original diagnosis of AD, and in its subsequent progression, because it can be easily performed in the routine care of patients. A lower score indicates more cognitive impairment. The highest (best) score is 30. Patients with MMSE scores above 26 or below 18 at baseline Visit 1 will be excluded.

The MMSE will be assessed at Visits 1, 5 and early discontinuation visit, if applicable.

Rater: Trained psychologist or physician

10.4.5 Hachinski Ischemic Scale

The Hachinski Ischaemic Score (HIS) is a commonly used clinical tool for the differentiation of the most common dementia types, Dementia of Alzheimer's Type (DAT) and Vascular Dementia (VaD). It has a cut-off score ≤ 4 for DAT and ≥ 7 for VaD[27].

11 STUDY MEDICATION (LM11A-31-BHS)

11.1 Identity of the investigational product(s)

Investigational drug product formulation and encapsulation:

Investigational Medicinal Product (IMP), LM11A-31-BHS, is imported packaged at and shipped by ACE Pharmaceuticals PO BOX 1262, NL-3890 BB, Zeewolde, The Netherlands in accordance with Good Manufacturing Practices (GMP).

Description and Composition of the DRUG Product

The LM11A-31-BHS drug product will consist of 361 mg LM11A-31-BHS drug substance (200 mg base), weighed and dry-filled into size 0 green hard gelatin capsules (Capsugel®) packed in PVC-PE-PVdC/Alu blister strips.

Placebo capsules

The placebo product will consist of microcrystalline cellulose with 0.5 – 1% magnesium stearate into size 0 green hard gelatin capsules (Capsugel®) packed in PVC-PE-PVdC/Alu blister strips. There will be no information about randomization transferred from the manufacturing company to the sponsor. To preserve blinding there will be no differences between LM11A-31-BHS capsules and placebo capsules in shape, size or color. The manufacturing organization will be strictly independent from the sponsor's activities.

11.2 Investigational drug product packaging

Hard gelatin capsules containing the study medication will be packaged in FORMPACK® (3-ply cold-formable aluminum plastic laminates) blisters. Each blister will contain 20 capsules for 5 days of treatment. They will be arranged in two rows of 10 capsules each with labelling as shown in Figure 6.

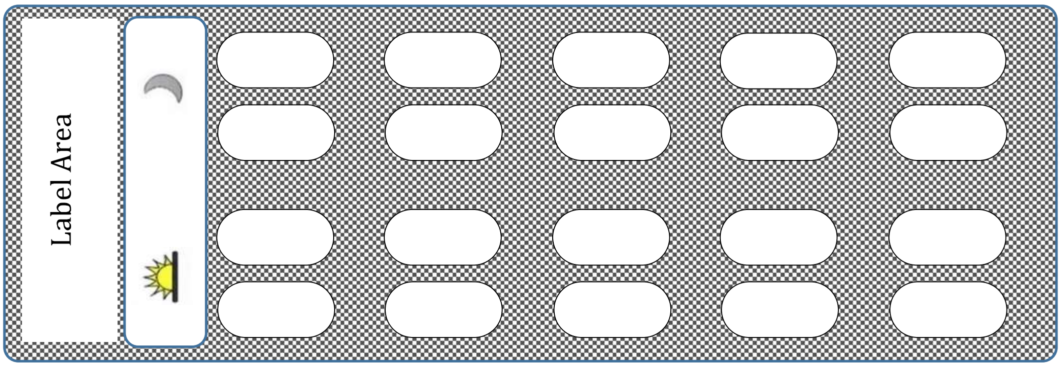

Figure 6: Blister Configuration

Each blister will contain 20 capsules. Capsules will be arranged in two vertical rows of ten capsules each, whereas lower row indicates the morning and the upper row the evening intake. The label is indicated by the rectangular box at the left side.

Medication will be dispensed one box per patient to the study centers identified by a unique three-digit randomization kit number. From these boxes, medication kits will be dispensed at each visit, starting with Baseline (V2: 2 kits -that is 10 blister cards, V3: 2 kits, V4: 4 kits). Each medication kit contains 5 blister cards (corresponding to 5 x 5 x 4=100 capsules). According to this distribution scheme, patients will have sufficient medication to continue treatment until the next visit is scheduled, even if full use is made of visit windows of plus 7 days at V5 (day 189). Unused medication and empty blister cards should be returned at each visit.

A medication label will be attached to each blister. Blister cards will be labelled in the local language. Multilingual labels will be printed in Swedish, Czech and German. Labels will identify the clinical study number (EudraCT), kit randomization number, sponsor, lot number and expiration date of the medication. A facsimile of the label in English is shown below.

In addition, a medication label will be attached to each kit describing the administration and storage of the investigational product, clinical study number (EudraCT), patient kit, randomization number, sponsor, emergency contact telephone number, lot number and expiration date of the medication. In addition, these labels will be printed in the Swedish, Czech and German language. A facsimile of the label in English is shown below. Further information will be provided in a booklet.

The five-digit patient identification number and the visit number will need to be written by the investigator onto the visit box after study medication has been assigned via the IWRS to a patient.

Re-labelling of the study medication expiry dates might be done during the study, since on-going study medication stability data will be collected during the trial re-labeling will be done by a qualified and/or trained person in agreement with local regulatory requirements.

Facsimile for blister labelling:

Clinical trial:	EudraCT no.
**NSC15001**	**2015-005263-16**
Visit No. **X**	Patient identification:
Randomization no.: 000	_ _ /_ _ _
LOT #: XXXXXX	EXP: MM-YYYY

Facsimile of medication box labelling:

Clinical trial: **NSC15001**	EudraCT no. **2015-005263-16**
Visit No. **X**	Patient identification: _ _ /_ _ _
Randomization no.: 000	EXP: MM-YYYY
LOT #: XXXXXX	

Capsules for oral administration: LM11A-31-BHS 200 mg or Placebo.

The blister card contains 20 capsules.

Swallow **TWO** capsules, marked with a sun symbol, with water each morning at breakfast and **TWO** capsules, marked with a moon symbol, each evening at dinner. Take capsules at the same time each day.

Do not store over 25°C. Store in a dry place and protect from light.

Keep out of reach of children.

Please bring all blister cards, including those with unused capsules, to every clinical visit with you.

For clinical trial use only.

Investigator: ________________________________

Sponsor: PharmatrophiX Inc., 2198 Sterling Avenue, Menlo Park, CA 94025, USA

Contact Number for Inquiries: +43 3132 40 444-10

11.3 Treatments administered

LM11A-31-BHS or the matching placebo will be administered twice daily as 2 capsules of identical appearance in a blinded manner:

2 capsules with 200mg placebo each in the morning and in the evening (total daily dosage of LM11A-31-BHS = 0mg).

1 capsule with 200mg (free base) LM11A-31-BHS and 1 capsule with placebo in the morning and in the evening (total daily dosage of LM11A-31-BHS = 400mg).

2 capsules with 200mg (free base) LM11A-31-BHS in the morning and in the evening (total daily dosage of LM11A-31-BHS = 800mg).

There is no hint in previous collected preclinical or clinical data that food consumption has any effect on the absorption of LM11A-31-BHS.

No intolerabilities were seen during the previous clinical trials either, but it is recommended to take the capsules together with food (breakfast and dinner) to enhance the tolerability.

Patients who meet all the eligibility criteria at the screening and baseline visits (Visits 1 and 2) will be dispensed study medication containing investigational product at each visit from baseline (Visit 2) onwards until visit 4. Patients will be instructed to take two capsule with water twice daily in the morning and in the evening.

On the first treatment day, the patients will be randomized to either placebo (n=80) or 200mg bid (n=80) or 400mg bid (n=80) LM11A-31-BHS.

A 1:1:1 (dose1: dose 2: control) allocation ratio will be used throughout the central randomization process and will be structured to allow for a total of at least 240 evaluable patients (80 per group) with center stratification and appropriate block size. If sample size adjustment is required at the interim analysis, the 1:1:1 allocation ratio will continue, as well as center stratification (including additional sites if required).

Patient randomization numbers and treatment codes will be agreed upon in advance between the clinical and statistical team and documented on a study-specific randomization request form. The electronic listing and SAS dataset prepared will be stored securely in password-protected files.

Each study center will receive a certain number of patient medication packages at the beginning of the study. Due to possible updating of the expiry date for the investigational product during the course of the trial, individual Patient Kits (or medication supply) may be shipped to investigational sites in a staged manner. The investigational sites will be provided with sufficient individual patient medication in accordance with their anticipated enrollment rate for the first two months. Thereafter the study sites will be supplied with additional medication according to the actual enrollment rate determined. The NeuroScios project manager or delegate will monitor recruitment and will contact the manufacturer for additional supply as appropriate.

Table 2: Dose schedule overview

	Morning Dose		Evening Dose		Total LM11A/d:
**Dose 1:**	LM11A 200mg	Placebo 200mg	LM11A 200mg	Placebo 200mg	400mg
**Dose 2:**	LM11A 200mg	LM11A 200mg	LM11A 200mg	LM11A 200mg	800mg
**Control:**	Placebo 200mg	Placebo 200mg	Placebo 200mg	Placebo 200mg	0mg

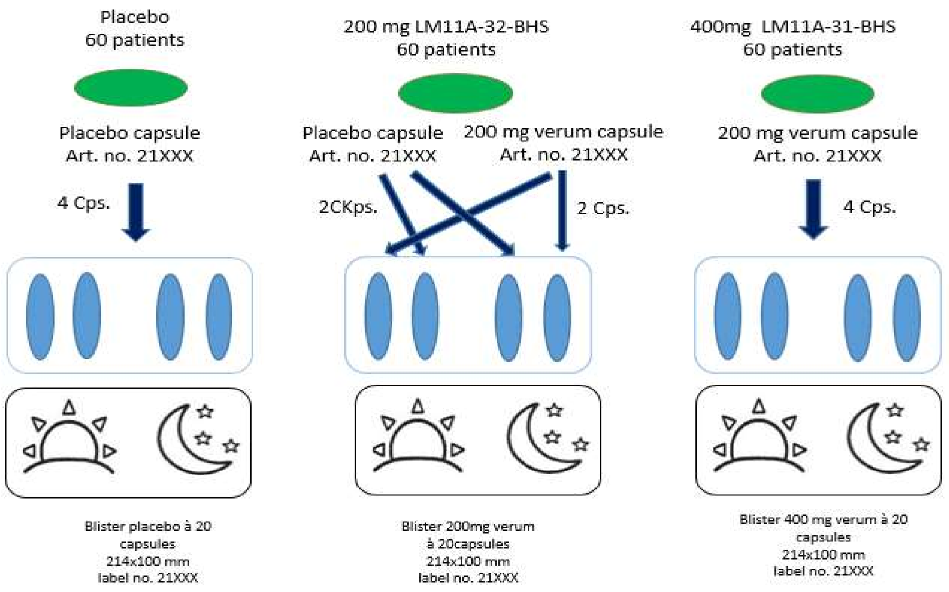

Figure 7*:* Administration Schedule

11.4 Selection of doses in the study

Adequate safety margins for 200mg / 400mg bid LM11A-31-BHS dosing have been established from both clinical and pre-clinical investigations.

11.5 Selection and timing of dose for each subject

Two capsules will be administered by oral route twice daily in the morning and in the evening. Doses should always be taken at approximately the same time together with an appropriate amount of water.

11.6 Blinding

The sponsors' personnel involved in this study as well as the personnel from NeuroScios, the study sites' personnel, the patients and the caregivers will be blinded to the assigned treatment. All investigational medication, *i.e.* LM11A-31-BHS and placebo capsules, will be identical in appearance. The unique patient identification number, which will be assigned to the patient at baseline visit (Visit 2), identifies the assignment of the patient to the treatment arm and

corresponds to the unique drug identification number. The randomization data are kept strictly confidential, accessible only to authorized persons until the time of database lock. Both NeuroScios and PharmatrophiX will be notified immediately when the study blind is broken.

In case of an emergency situation, unblinding of individual patients by the site investigator might be necessary for appropriate treatment of a medical emergency. For this purpose, individual access codes will be provided to each investigator, to the study safety officer and to the project manager of the CRO. The Investigator should break the code only if knowledge of the medication is necessary for optimal treatment in an emergency situation. If possible, the Principal Investigator, the study monitor or safety officer should be consulted before breaking the code. The date, time and reason for breaking the code will be recorded in the IWRS system.

The safety data from the patients concerned will continue to be collected but any efficacy will only be included in the analysis up to the date of the code break in question.

In case of a potential Suspected Unexpected Serious Adverse Reaction (SUSAR, see 14.7), unblinding of the specific patient by the Study Safety Officer will be needed to verify the SUSAR prior to expedited reporting to Eudravigilance and ECs.

11.7 Prior and Concomitant Therapy

Concomitant medication is defined as any medication, other than the IMP, which is taken during the trial, including prescription and over-the-counter medications.

The medication history over the past three months will be obtained from each patient at the time of screening and will be recorded in the recent and concomitant medication form in the CRF.

Concomitant medications, if these are considered necessary for the patients' welfare and will not interfere with the study medication, may be given at the discretion of the investigator. Concomitant medications are permitted during the trial period except the medications listed under section 11.8 "Prohibited Concomitant Medications" of the trial protocol.

At screening, the medication history over the past three months prior to screening will be obtained from each patient and will be recorded in source data and on the concomitant medication page in the e-CRF.

Conventional Alzheimer's disease therapies are permitted and must be on stable doses for at least 3 weeks prior to Visit 1 and during the entire trial period. A change in dose of standard treatments for Alzheimer's disease within three months prior to screening is reason not to include the patient in this trial.

The patient may also not be included, if previous or current medications which the patient is taking because of consisting illnesses are changed within 30 days prior to screening, if considered relevant by the investigator.

All medication that the patient is taking at the time of the screening visit and all subsequent study visits will be recorded in the source data and on the concomitant medication page in the e-CRF. Additionally, any changes in these medications, any changes in frequency and/or dosing or any new medication introduced during the course of the study will be recorded on the concomitant medication page and, if the change in concomitant medication is considered relevant by the investigator, as a protocol deviation. For any concomitant medication (including decrease and increase in dose) given as treatment for a new or worsened condition, the Investigator must also record this condition as an AE in the e-CRF.

11.8 Prohibited Concomitant Medications

The administration of the following medications interferes with the evaluation and interpretation of the results of the clinical trial and is therefore excluded throughout the entire clinical trial. Patients who are receiving one of the following medications should be excluded.

Chronic daily drug intake of ≥ 14 days or expected for ≥ 14 days:

- Benzodiazepines, neuroleptics or major sedatives
- Antiepileptics
- Centrally active anti-hypertensive drugs (clonidine, l-methyl DOPA, guanidine, guanfacine, etc.)
- Opioid containing analgesics
- Nootropic drugs (except Ginkgo Biloba)

11.9 Permitted Concomitant Medications

Stable treatment with one of the acetylcholinesterase inhibitors donepezil (Aricept ®), galantamine (Razadyne®), or rivastigmine (Exelon) or the partial NMDA receptor antagonist with memantine (Namenda®) is allowed in a steady state (chronic use and stable dosage for at least three months prior to baseline). Dosage regimen follows empirical clinical judgements and is based on a case-by-case evaluation.

11.10 Treatment Compliance and Drug Accountability

Assessment of compliance: At Visits 3, 4 and 5 the patients/caregivers will return to the site bringing their boxes of blister strips with them for an assessment of medication compliance.

At Visits 2, 3 and 4, new medication boxes will be dispensed to the patients. In addition, remaining unused medication will be redistributed at Visits 3 and 4 to guarantee a sufficient supply for the intervening treatment period until the next study visit.

Patients will be instructed to adhere to the dosing schedule, because it is critical to the performance of the investigational product and to the assessment of its potential benefit. Patients should be informed that dosing should be done on a regular schedule daily (*i.e.* take morning and evening doses at approximately the same time each day).

Concerning missed doses: patients missing less than 20% of the study medication over the course of the complete trial period will be treated as per protocol. Patients missing more than 20% of the study medication over the course of the complete study should be excluded from the study per protocol analysis. Also, if any evidence exists that patients have missed more than 5 consecutive doses they will be excluded from the per protocol analysis.

At Visit 2, 3 and 4, as discussed in Section 11.3, a sufficient amount of medication will be given to patients/caregivers for use as spare medication to cover the time until the maximum visit-window (+7 days). In the event of a disruption in investigational drug supply, patients/caregivers should notify their investigational site immediately (*e.g*. loss of trial box, damage to trial box) in order to arrange a resupply.

1. Dosing interruptions or dose reductions should not be allowed unless the patient is experiencing an AE. Should an AE occur during dosing and the patient is concerned with continuation of treatment, the patient or caregiver should notify his/her investigational site immediately.

2. If the Investigator determines that dosing should be interrupted, this information should be documented and communicated to NeuroScios immediately, and subsequently communicated to the safety officer. If the patient's AE does not resolve within three days, and if the Investigator (in consultation with the NeuroScios and sponsor's safety officer) determines that the AE is related to LM11A-31-BHS treatment, dosing should not be re-initiated but data will continue to be recorded.
3. If a patient experiences an AE with a similar symptomatic profile upon re-initiation of any dosing, the investigator should terminate dosing. but data will continue to be recorded.

If an individual dose is missed, the following guidelines are provided.

1. If the missed dose is the morning dose, then the patient should take the missed morning dose as soon as possible thereafter on the same day but not later than lunch-time. Otherwise the morning dose is skipped.
2. If the missed dose is the morning dose and the morning dose is then taken later in the day, the evening dose can be taken if at least six hours have elapsed post morning dose and it is at least three hours prior to going to sleep; if not, then skip evening dose.
3. If the missed dose is the evening dose, then the dose can be taken at any time up to at least three hours prior to going to sleep.
4. If the missed dose is the evening dose and it is not taken that day, then the next dose will be the morning dose of the next day. The evening dose is skipped.
5. Never attempt to compensate for a missed dose by taking two doses at the next scheduled dosing period.
6. The patient should never take two doses within less than six-hours.

11.11 Drug Accountability at the Site

The Investigator will confirm receipt of the investigational product in writing (date, time and storage condition) and will use the investigational product only within the framework of this clinical study and in accordance with this trial protocol. Receipt and return of the investigational product must be properly documented and include the following information: study number, dates (dispensed and returned), quantities, batch/lot number, expiry date, and the code number assigned to the investigational product and study patients.

At the beginning of the study, the site will receive drug accountability forms to document, dispense and return of study medication. These forms will be made available to the authorized persons (*e.g.* Clinical Monitor, Auditor) and include the following information: study number, dates, quantities, batch/lot number, expiry date and the code number assigned to the investigational product and study patients.

The responsible CRA will check the drug supply for inventory purposes and to ensure proper storage will be conducted at regular intervals throughout the study.

12 LABORATORY

All laboratory safety tests will be analyzed by the central laboratory, Labor Dr. Spranger und Partner; D-85051 Ingolstadt, Lindberghstrasse 9-13; Germany. The laboratory will provide instructions on blood sampling methodology and supplies to the staff prior to initiation of the study.

For blood collection, the following tubes will be used:

7, 5 ml plain monovettes for biochemistry screens

2,7 ml EDTA monovettes and 3 ml Citrate monovettes for all hematology screens.

The total blood volume collected from each individual during the entire study will be approximately 115 ml.

Table 3: Blood volume collected

**Biochemistry**	5 x 7,5 ml	37,5 ml
Glucose	5 x 2,7 ml	13,5 ml
Homocystein	5 x 2,7 ml	13,5 ml
**Hematology**	5 x 2,7 ml	13,5 ml
**Coagulation**	5 x 3 ml	15ml
**Pharmacodynamic and biomarker assessments**	4 x 3 ml	12 ml
**Serology**	1 x 7,5 ml	7,5 ml
**Genotyping**	1 x 2,7 ml	2,7 ml
	Total blood volume entire study:	≈ 115,2 ml

12.1 Diagnostic Parameters

Five milliliters (5 ml) of blood will be collected in gel tubes for determination of diagnostic parameters at screening V1 and prepared according to the guidelines provided in the laboratory manual. The amount of folic acid and vitamin B12 will be determined in the local laboratory at the site. Becton Dickenson Vacutainers will be used for blood sampling.

Table 4: Diagnostic parameters

**Other**	HbA1c (V1), ApoE ε4 allele status
**Serology**	Syphilis (TPPA), HIV, Hepatitis B, Hepatitis C (V1).
**Hormonal & nutritional status**	Thyroid panel (T4, TSH), Vit. B12, Folate (V1)
**CSF**	CSF-Biomarkers (tau, p-tau, Aβ40, Aβ42, AchE activity)

12.2 Safety Blood and Urinalysis

Safety parameters will be collected at each visit (Visit1 to Visit 5). Laboratory values (hematology, clinical chemistry and urinalysis) outside the reference range will be classified as either clinically significant or not clinically significant by the Investigator. Repeat samples may be requested by the Investigator. All new findings or worsening of clinically significant abnormalities will be recorded as AEs and followed-up by the Investigator.

If any parameter is not measured, ND for "not done" will be entered in the appropriate space in the CRF.

Table 5: Safety parameters

**Hematology:**	Hematocrit, hemoglobin, erythrocytes (red blood cells), MCV, MCHC, MCH, reticulocytes, leucocytes (white blood cells), differential blood count (neutrophils, lymphocytes, monocytes, eosinophils and basophils, expressed in total number and % of leucocytes) and thrombocytes (platelets).	
**Biochemistry:**	Electrolytes:	Sodium, potassium, calcium, chloride and inorganic phosphate, magnesium
	Enzymes:	SGOT (AST), SGPT (ALT), alkaline phosphatase, GGT, LDH, creatine-kinase
	Substrates:	Glucose, total cholesterol, triglycerides, creatinine, creatinine clearance, total bilirubin, total protein, albumin, uric acid and blood urea nitrogen (BUN), homocysteine, CRP, HbA1c,<br>Cystatin C (if GFR ≤30ml/min)
**Coagulation**	PTT, Fibrinogen, INR, D-dimer	
**Urinalysis:**	Dipstick analysis will be performed at the trial site and includes: pH, blood leucocytes, protein, glucose, specific gravity, nitrites, urobilinogen, bilirubin and ketones. If clinically relevant abnormalities are detected, a urine sample will be sent to the local laboratory for analysis of the sediment.	

12.3 Pharmacokinetics

PK sampling will be performed during Visit 2, 3, 4 and 5. During Visit 2 the PK sample will be taken before the first dose is taken. The patients will record the time when the morning dose was taken on visit days 3,4,5. The time of PK sampling will be recorded as well.

12.4 Lumbar puncture for CSF collection

All CSF samples will be analyzed at the Clinical Neurochemical Laboratory, Inst. of Neuroscience and Physiology, The Sahlgrenska Academy at Gothenburg University, Mölndal Hospital, SE-431 80 Mölndal, Sweden.

CSF sampling will be performed at Screening Visit 1 and Visit 5. The puncture is to be done after obtaining results of the brain MRI. CSF samples collected (max. 15 ml) will be processed according to the appended Laboratory Manual, shipped to the Sponsor and analyzed.

Lumbar puncture (LP)

The LP will be performed in the morning (AM). The LP will be made in the L3/L4 or L4/L5 interspace, patient sitting or lying down, fasting not needed. 10 ml of CSF is tapped into a polypropylene tube.

In order to reduce the risk of postdural headache, lumbar puncture must be performed with atraumatic needles.

After lumbar puncture, the following procedures are recommended according to Good Clinical Practice:

1. Patient must stay in bed on the abdomen 30 minutes after spinal tap
2. After these 30 minutes on abdomen patient stays additional 90 minutes in bed
3. After these 2 hours (30+90 minutes), patient can leave outpatient department and go home.

In addition, the patients need to follow the instructions of the investigator and the particular hospital procedures in case any other medical reasons.

Biomarkers tau, p-tau, Aβ40, Aβ42 activity and AchE activity will be measured from patient's CSF samples. Selected samples will be used to measure drug levels. CSF levels of LM11A-31 and its aminoethyl morpholine metabolite will also be measured.

If CSF samples have been obtained from a patient within a three-month window before screening these may be used for inclusion criteria check-up.

In case there are already results, which confirm that the patient can be included into the study, these results can be used for inclusion.

In case Aβ42, p-Tau or Tau were not analyzed an aliquot of at least 1 ml (see laboratory manual) need to be sent to the laboratory in Sweden.

In any case, aliquots from the baseline samples are needed to be analyzed at the end of the study together with the samples from the final visit.

If CSF sampling is done at the site as a routine for diagnostic purposes, the procedures for sampling of CSF described in the laboratory manual of the study should be followed if possible. Aliquots of these sample should be stored as described in the manual.

If the patient has already been screened before and could not be included because of the T-tau or p-tau values but Aβ42 <550 ng l−1 or a Aβ 40/42 ratio <0,89 is suitable, these patients can be rescreened and included based on the former CSF results.

13 STUDY PROCEDURES

13.1 Flow Chart

Clinical trial flow chart	Visit 1 (Screening)	Visit 2 (Baseline)	Visit 3	Visit 4	Visit 5 (Final Visit)	Early Disc. Visit
Week	-8	0	4	12	26	
Days (+/- Days)	-56	1 (+7)	28 (+/-7)	84 (+/-7)	182 (+/-7)	
Informed Consent Capability	X					
Written Informed Consent [1]	X					
Demographic Information	X					
General Medical and Surgical History	X					
Verification of Inclusion/Exclusion criteria	X	X				
Diagnosis of probable AD (McKhann et al)	X					
Physical Examination	X	X	X	X	X	X
Neurological Examination	X	X	X	X	X	X
Vital Signs [2]	X	X	X	X	X	X
Hematology, Biochemistry, Urinalysis[3]	X	X	X	X	X	X
Coagulation	X					
Serology: Syphilis, HIV, HepB, HepC, INR	X					
Thyroid Panel (T4, TSH), B12, folate [4]	X					
Pharmacokinetic samples		X	X	X	X	X
CSF sampling [5]	X				X	X
Apo E Status		X				
Modified Hachinski Ischemic Score	X					
Geriatric Depression Scale	X	X	X	X	X	X
C-SSRS	X	X	X	X	X	X
History of Alzheimer's disease	X					
ECG [6]	X		X	X	X	X
MRI [7]	(X)	X			X	X
MMSE	X				X	X
^{18}F-FDG-PET		X			X	X
NTB: Category Fluency Test, COWAT, Digit Symbol Substitution Test, Digit Span		X		X	X	X
ADAS cog-13-items		X		X	X	X
AMUNET		X		X	X	X
Clinical Global Impression - Severity	X					
Clinical Global Impression - Improvement				X	X	X
Patient Card Handout	X					
Patient Diary Handout and/or Return		X	X	X	X	X
Randomization		X				
Dispensing or return of Study Medication		X	X	X	X	X
Recording of Concomitant Medication [8]	X	X	X	X	X	X
Recording of Adverse Events		X	X	X	X	X

1. Informed Consent must be obtained from patient and caregiver before any study examination is performed
2. Vital signs: heart rate, systolic, diastolic blood pressure, body temperature and respiratory rate will be measured after five minutes in sitting position. Weight will be measured at every visit. Height will be measured only at screening and Visit 5.
3. Hematological parameters: Erythrocytes, hemoglobin, hematocrit, MCH, MCV, leucocytes, neutrophils, eosinophils, basophils, lymphocytes, monocytes and thrombocytes. Blood chemistry: total bilirubin, ALT, AST, LDH, alkaline phosphatase, gamma-GT, creatinine, Blood Urea Nitrogen, uric acid, CK, sodium, potassium, phosphate, calcium, chloride, magnesium, albumin, total cholesterol, triglycerides, glucose, total protein, INR (prothrombin time). Urinalysis: pH, glucose, albumin, ketones, specific gravity, nitrites, bilirubin, urobilinogen, red and white blood cells.
4. Assessment for TSH, free T4, folate, vitamin B12 and TPPA test for syphilis (Vitamin B12 and folate will be analyzed in local laboratories at the sites)
5. CSF biomarker assessments: p-tau and tau, Aβ40, Aβ42 tau, p-tau, Aβ40, Aβ42, and AChE. If CSF samples are taken at the Screening Visit (Inclusion Criteria Nr.4), aliquots of the samples will be sent to the laboratory and analyzed immediately. All other sample assessments will be performed at the end of study unless the trial is determined to be a total failure. In this case, a determination will be made as to whether to perform these tests to better understand trial data.
6. ECG: 12-lead ECGs will be recorded after 5 minutes in supine posture.

7. A MRI according to the MRI protocol will be performed at the screening visit if no MRI is available within 6 months prior to baseline visit. All patients need to have a MRI according to the MRI protocol at baseline if no MRI was performed at the screening visit.
8. Recording of Concomitant Medication including general AD treatment (& changes thereof)

13.2 Screening Procedures at Visit 1

The screening has to be performed within eight weeks prior to the baseline Visit.

The following assessments will be performed at screening for each patient:

- The informed consent capability will be verified by an independent physician/ neurologist in Germany and Czech Republic.
- Informed consent procedure: Prior to performing any study related assessments, the Investigator will inform the patient and caregiver in writing and orally about the potential benefit and any risks associated with the participation in this trial. The informed consent must be signed and dated by the patient and the caregiver. A separate Informed Consent must be signed by the patient for the genetic testing. The investigator will document the Informed Consent Procedure in the patient file.
- Demographics: sex, race, date of birth (Only for Germany: Date of birth shall not be reported) and domiciliary status
- Medical, surgical and drug history: A drug history will be recorded for the period of three months prior to screening
- Compliance with inclusion/exclusion criteria
- Physical and Neurological Examination
- Vital signs, including
  - Systolic and diastolic blood pressure (after 5 min sitting)
  - Heart rate (after 5 min sitting)
  - Respiration rate (after 5 min sitting)
  - Body temperature (after 5 min sitting)
  - Height
  - Weight
  - Body Mass Index (BMI)
- Laboratory safety screens: biochemistry, hematology, urine analysis
- Diagnostic laboratory: Assessment for TSH, free T4, folate, vitamin B12 and TPPA test for syphilis, HIV, Hep B, Hep C, coagulation
- Modified Hachinski Ischemic Score
- Geriatric depression scale (GDS):< 5
- C-SSRS
- History of Alzheimer's Disease
- Verification of diagnosis of probable Alzheimer's Disease according to McKhann et al (2011)
- 12-lead ECGs will be recorded after 5 minutes in supine posture
- A MRI will be performed if no MRI or CT is available no older than six months before baseline
- CSF sampling (tau, ptau, Aβ40, Aβ42, AchE activity)
- MMSE: ≥18 and ≤26
- Clinical Global Impression – Severity (CGI-S)
- Assessment of concomitant medication, general AD treatment and changes thereof
- Patient card handout
- Recording of concomitant medication

13.3 Baseline Procedures at Visit 2:

A visit window of +7 days is allowed for this visit.

The following assessments will be performed at baseline for each patient:

- Physical and Neurological Examination
- Compliance with Inclusion/Exclusion Criteria, if applicable
- Vital signs, including
  - Systolic and diastolic blood pressure and pulse (after 5 min sitting)
  - Respiration rate (after 5 min sitting)
  - Body temperature
  - Weight
  - Body Mass Index (BMI)
- Laboratory safety screens: biochemistry, hematology, urine analysis
- Pre-dose PK sampling
- Apo E Status
- MRI according to the MRI protocol if no MRI was performed during the screening visit
- Geriatric depression scale (GDS)
- C-SSRS
- FDG-PET Scan
- NTB:
  - Category Fluency Test
  - Digit Span
  - Controlled Oral Word Association test (COWAT)
  - Digit Symbol Substitution Test
- ADAS-cog 13 items
- Spatial Orientation Testing with Amunet
- Patient diary handout and/or return
- Randomization
- Study Medication dispensing (Medication Kit 1 and 2)
- Recording of concomitant medication
- Recording of adverse events

13.4 Procedures at Visit 3 (4 Weeks):

A visit window of +/- 7 days is allowed for this visit.

The following assessments will be performed at Visit 3 for each patient:

- Physical and Neurological Examination
- Vital signs, including
  - Systolic and diastolic blood pressure and pulse (after 5 min sitting)
  - Respiration rate (after 5 min sitting)
  - Body temperature (after 5 min sitting)
  - Weight
  - Body Mass Index (BMI)
- Laboratory safety screens: biochemistry, hematology, urine analysis
- PK sampling
- Geriatric depression scale (GDS)
- C-SSRS
- 12-lead ECGs will be recorded after 5 minutes in supine posture
- Patient diary handout and/or return
- Study Medication dispensing (Medication Kit 3 and 4 plus unused returned capsules from pervious boxes)

- Recording of concomitant medication
- Recording of adverse events

13.5 Procedures at Visit 4 (12 Weeks):

A visit window of +/- 7 days is allowed for this visit.

The following assessments will be performed at Visit 4 for each patient:

- Physical and Neurological Examination
- Vital signs, including
    - Systolic and diastolic blood pressure and pulse (after 5 min sitting)
    - Respiration rate (after 5 min sitting)
    - Body temperature (after 5 min sitting)
    - Weight
    - Body Mass Index (BMI)
- Laboratory safety screens: biochemistry, hematology, urine analysis
- PK sampling
- Geriatric depression scale (GDS)
- C-SSRS
- 12-lead ECGs will be recorded after 5 minutes in supine posture.
- NTB:
    - Category Fluency Test
    - Digit Span
    - Controlled Oral Word Association test (COWAT)
    - Digit Symbol Substitution Test
- ADAS-cog 13 items
- Spatial Orientation Testing with AMUNET
- Clinical Global Impression – Improvement (CGI-I)
- Patient diary handout and/or return
- Study Medication dispensing (Medication Kit 5, 6, 7 and 8 plus unused returned capsules from previous boxes)
- Recording of concomitant medication
- Recording of adverse events

13.6 Procedures at Visit 5 (26 Weeks):

A visit window of +/- 7 days is allowed for this visit.

The following assessments will be performed at visit 5 for each patient:

- Physical and Neurological Examination
- Vital signs, including
    - Systolic and diastolic blood pressure and pulse (after 5 min sitting)
    - Respiration rate (after 5 min sitting)
    - Body temperature (after 5 min sitting)
    - Height
    - Weight
    - Body Mass Index (BMI)
- Laboratory safety screens: biochemistry, hematology, urine analysis
- PK sampling
- CSF sampling (tau, ptau, Aβ40, Aβ42, AchE activity, in selected patients drug levels)
- MMSE

- Geriatric depression scale (GDS)
- C-SSRS
- 12-lead ECGs will be recorded after 5 minutes in supine posture
- MRI
- FDG-PET scan
- NTB:
  - Category Fluency Test
  - Digit Span
  - Controlled Oral Word Association test (COWAT)
  - Digit Symbol Substitution Test
- ADAS-cog 13 items
- Spatial Orientation Testing with Amunet
- Clinical Global Impression – Improvement (CGI-I)
- Patient diary return
- Recording of concomitant medication
- Recording of adverse events
- Return of study medication

13.7 Procedures at Early Discontinuation Visit

The same assessments as for final visit 5 apply to an early discontinuation visit:

- Physical and Neurological Examination
- Vital signs, including
  - Systolic and diastolic blood pressure and pulse (after 5 min sitting)
  - Respiration rate (after 5 min sitting)
  - Body temperature (after 5 min sitting)
  - Height
  - Weight
  - Body Mass Index (BMI)
- Laboratory safety screens: biochemistry, hematology, urine analysis
- PK sampling
- CSF sampling (tau, ptau, Aβ40, Aβ42, AChE activity)
- MMSE
- Geriatric depression scale (GDS)
- C-SSRS
- 12-lead ECGs will be recorded after 5 minutes in supine posture.
- MRI
- FDG-PET scan
- NTB:
  - Category Fluency Test
  - Digit Span
  - Controlled Oral Word Association test (COWAT)
  - Digit Symbol Substitution Test
- ADAS-cog 13 items
- Spatial Orientation Testing with Amunet
- Clinical Global Impression – Improvement (CGI-I)
- Patient diary return
- Recording of concomitant medication
- Recording of adverse events

- Return of study medication

14 SAFETY REPORTING, PHARMACOVIGILANCE

Management of pharmacovigilance activities will be performed by NeuroScios PV Unit.

Details on responsibilities and handling of safety reporting processes will be outlined in the corresponding Safety Workplan which, once approved and signed, is binding for all parties involved in pharmacovigilance within the trial.

14.1 Adverse Events (AEs)

According to ICH E2A, an AE is any untoward medical occurrence in a patient or clinical trial investigation subject administered a pharmaceutical product, which does not necessarily have a causal relationship with the treatment. An AE can therefore be any unfavorable or unintended sign (including, for example, an abnormal laboratory finding), symptom, or disease temporally associated with the use of a medicinal product, whether or not related to the medicinal product.

All AEs should be recorded by the investigator, whether considered minor or serious, drug-related or not.

AEs will be ascertained based on volunteered symptoms, responses to non-leading non-specific questions and clinical observation and assessment. All abnormal laboratory findings considered clinically significant must be recorded as AEs.

An AE can therefore be any unfavorable and unintended sign, symptom or disease including inter-current illness, deterioration of a pre-existing illness, accident, any suspected drug reaction or a clinically relevant change of laboratory values, ECG, *etc.,* whether or not considered related to the investigational product(s) and/or study treatment.

14.2 Documentation

Data pertaining to AEs will be collected during each study visit either based upon the patient's spontaneous description, by Investigator's inquiry or discovered in the course of examinations completed during the visit. The Investigator should first ask a general question, for example, "Have you experienced any other/new health problems since your last visit?" rather than searching for specific symptoms. If the answer is, "no", no further questions are asked. If the answer is "yes", the Investigator will assess and record any AE in detail on the 'Adverse Event' page of the Case Report Form including: AE description, the date (and time, if applicable) of onset, date (and time, if applicable) of resolution, maximum intensity, seriousness, duration and outcome, drug relationship of the AE to the study drug, any study drug action and/or any other action taken. After all visit investigations (*e.g*. ECG, physical and neurological examination, vital signs) are performed, the Investigator must document any AEs detected during these visits in the patient files.

All of the following details will be recorded in the patient's CRF for each adverse event:

- Full description of adverse event
- Date of onset
- Date of resolution
- Severity of event, to be assessed by the investigating physician in accordance with the definitions below
- Relationship to study drug to be assessed by the investigating physician in accordance with the definitions below
- Action taken (if any)
- Outcome and details of any further follow-up

Furthermore, in case of a Serious Adverse Event (SAE, see section 14.6), the Investigator must additionally complete a "Serious Adverse Event Report" and report the SAE to NeuroScios immediately as described below.

14.3 Definition of severity

The following definitions should be used by the investigating physician to describe the severity of adverse events. The following are THE ONLY DEFINITIONS which should be used to describe adverse event severity. Only one severity definition should be used for each adverse event (e.g. MILD to MODERATE is not acceptable).

SEVERITY	DEFINITION
MILD	The adverse event does not interfere with the subject's/ patient's daily routines. It causes slight discomfort.
MODERATE	The adverse event interferes with some aspects of the subject's/ patient's daily routines.
SEVERE	The adverse event causes inability to carry out the subject's/ patient's daily routine.

14.4 Definition of causality relationship with investigational product

The following definitions should be used by the investigating physician to describe the relationship between an adverse event and investigational product. The following are the only definitions that should be used to describe the relationship between adverse events and the investigational product. Only one of the following definition should be used for each adverse event (e.g. POSSIBLE-PROBABLE is not acceptable).

RELATIONSHIP TO STUDY DRUG	DEFINITION
Definite	• Definite temporal relationship with IP treatment.<br>• Known reaction to agent or chemical group, or predicted by known pharmacology.<br>• Event cannot be explained by subject's/ patient's clinical state or other factors.
Probable	• Reasonable temporal relationship with IP treatment.<br>• Likely to be known reaction to agent or chemical group, or predicted by known pharmacology.<br>• Event cannot easily be explained by subject's/ patient's clinical state or other factors.
Possible	• Reasonable temporal relationship with IP treatment.<br>• Event could be explained by subject's/ patient's clinical state or other factors.
Unlikely	• Poor temporal relationship with IP treatment and/or<br>• Event easily explained by subject's/ patient's clinical state or other factors.
Not related	• Event occurred before dosing or<br>• Event or inter-current illness wholly due to factors other than drug treatment.
Not assessable	• Causality assessment cannot be performed at the time due to missing information.

14.5 Categories of actions taken and outcome

14.5.1 Action(s) taken to drug:

- Drug withdrawn
- Dose reduced
- Dose increased
- Dose not changed
- Unknown
- Not applicable

14.5.2 Outcome

- recovered/resolved
- recovering/resolving
- not recovered/not resolved
- recovered/resolved with sequelae
- fatal
- unknown

14.6 Serious Adverse Event (SAE)

A ”Serious adverse event" is any adverse event which

(a) Results in death,

(b) Is life threatening,

(c) Requires hospitalization or prolongation of existing hospitalization,

(d) Results in persistent or significant disability or incapacity, or

(e) Consists of a congenital anomaly or birth defect.

(f) Other medically significant event

Medical and scientific judgment should be exercised in deciding whether expedited reporting is appropriate in other situations, such as important medical events that may not be immediately life-threatening or result in death or hospitalization but may jeopardize the patient or may require intervention to prevent one of the other outcomes listed in the definition above. These should also usually be considered serious. Examples of such events are intensive treatment in an emergency room or at home for allergic bronchospasm, blood dyscrasias, convulsions that do not result in hospitalization or development of drug dependency or drug abuse.

All SAEs which occur during the study, from the first administration of the investigational medicinal product up to one month after receiving the last dose of study treatment, whether or not related to the study treatment will be documented and reported immediately to NeuroScios PV.

The immediate reports should be followed promptly by detailed follow-up reports. The immediate and follow-up reports should identify patients by unique code numbers assigned to the trial subjects rather than by the patient's names, personal identification numbers, and/or addresses.

All SAEs must be reported to the Sponsor within 24 hours of investigator awareness. The immediate reports should be followed promptly by detailed follow-up reports. The reports should identify patients by unique code numbers assigned to the trial subjects rather than by the patient's names, personal identification numbers, and/or addresses.

Details on reporting procedures will be given to all investigators involved in the trial prior to study start.

14.7 Expedited Reporting to Competent Authorities and Ethics Committees

Adverse events, which meet all of the following criteria:

- Serious
- Unexpected (*i.e.* is not consistent with the applicable product information)
- There is at least a reasonable possibility that there is a causal relationship between the event and the medicinal product

will be classified as **Suspected Unexpected Serious Adverse Reactions (SUSARs)** and should be reported to the Ethics Committee and to the Regulatory Authorities and investigators in accordance with applicable regulatory requirements for expedited reporting. It is the Sponsor's or delegate's responsibility to report SUSARs to the Ethics Committee and Regulatory Authorities.

Full details of the SAE handling and SUSAR reporting will be documented in a study specific pharmacovigilance plan created by NeuroScios prior to the start of dosing.

14.7.1 Definition of serious and severe

To ensure no confusion or misunderstanding of the difference between the terms "serious" and "severe," which are not synonymous, the following note of clarification is provided:

The term "severe" is often used to describe the severity of a specific event (as in mild, moderate, or severe myocardial infarction); the event itself, however, may be of relatively minor medical significance (such as severe headache). This is not the same as "serious," which is based on patient/event outcome or action criteria usually associated with events that pose a threat to a patient's life or functioning. Seriousness (not severity) serves as a guide for defining regulatory reporting obligations.

14.7.2 Monitoring of Patients with Adverse Events

Any adverse event, which occurs in the course of the study, should be monitored and followed up until:

- It is resolved.
- There is a return to normal or baseline values or reaches clinical stability.
- A plausible explanation for the cause of the event has been found.
- All requested follow up items have been received or confirmed as not available.

14.7.3 Unexpected Adverse Events

An Unexpected AE is an experience not previously reported in nature, severity or incidence in the current Investigator's Brochure, the general investigational plan or the SMPC, if applicable.

14.7.4 Expected Adverse Events

As with any medicinal product, it is possible that the study drug LM11A-31-BHS may lead to side effects during treatment. The expected adverse reactions are outlined in the IB.

15 DATA QUALITY CONTROL AND QUALITY ASSURANCE

This study will be monitored regularly by a Clinical Monitor according to ICH-GCP Guidelines and the respective Standard Operating Procedures (SOPs). Monitoring procedures include one or more visits designed to clarify all prerequisites before the study commences. Interim monitoring visits will take place on a regular basis according to a schedule fixed by mutual agreement. During these visits, the Clinical Monitor will check for completion of the entries in the e-CRF, their compliance with the trial protocol and with Good Clinical Practice, and will compare the e-CRF entries with the source data. Monitoring will also be aimed to detect any misconduct or fraud.

For source data verification, at least the following information must be included in the patient file:

- date of patient's written informed consent
- study number,
- screening number
- patients' visit dates
- (Serious) Adverse Events
- confirmation of the diagnosis of the indication being treated
- investigational product administration (*e.g.* start of treatment, dose compliance, drug accountability)
- concomitant medication
- primary efficacy variables
- secondary efficacy variables
- protocol deviations and/or violations

Laboratory and ECG printouts, including MRI/ CT data, are considered as source data. The Clinical Monitor will check the appropriate copy or copies of the completed forms. The Clinical Monitor will also verify the storage of the investigational medicinal product on site. The investigational medicinal product will not be supplied to the study site until a favorable opinion is obtained from the relevant IEC/IRB and Regulatory Authority and until Sponsors' approval. The Clinical Monitor will check the investigator site file, sample storage and drug storage and drug accountability including shipment conditions. After the final drug accountability has been performed, the investigational medicinal product will be destroyed on-site or returned to the sponsor.

In addition, the Clinical Monitor will determine whether all AEs and SAEs have been appropriately reported within the periods required.

The Investigator and his/her staff will be expected to cooperate with the Clinical Monitor providing any missing information whenever possible. The Investigator must be available to answer questions arising during regular monitoring of the study. In addition, the Investigator guarantees:

- Prior to each monitoring visit, all data will be properly recorded in the e-CRF and patient files.
- At the monitoring visits, source documentation will be available.
- All medication dispensed must be recorded in the e-CRF and/or drug inventory form.
- All patients who are screened but not entered into the study will be listed on the form provided.

The monitor will verify training of the site staff according to the delegated tasks and help the investigator to organize training and updates if applicable.

The sponsor will implement a quality assurance system to ensure that the trial is performed and the data are generated, documented (recorded), and reported in compliance with GCP and the applicable regulatory requirement(s).

A member of the Sponsor's quality assurance staff may arrange to visit the Investigator in order to audit the performance of the study at the study site and the study documents originating there. The Clinical Monitor and/or Project Manager will usually accompany the auditor(s). The Investigator will be informed about the scheduling and the outcome of any audit.

In addition, inspections by Regulatory Authority representatives and Independent Ethics Committees are possible at any time. The Investigator is to notify NeuroScios of any such inspection immediately and share any output of the inspection.

16 STATISTICAL METHODS AND DETERMINATION OF SAMPLE SIZE

Statistical methods are outlined, however a detailed Statistical Analysis Plan (SAP) including detailed descriptions of all statistical methodology utilized, planned 'sensitivity' analyses, together with master table and listing shells and outline figures will be produced after finalization of the electronic Case Report Form (e-CRF) and prior to the database lock of this study.

In addition, a supporting Data Review Plan (DRP) for the selection and presentation of all blinded data at the interim review will also be produced.

Any deviations or changes to the plans during the analyses will be documented and justified in the Final Clinical Study Report.

16.1 Adaptive Design

An adaptive design will be used for the study. In the context of this study, an adaptive design is used to describe the potential to increase the total sample size of patients required at some interim stage of the ongoing study.
As described in section 'Total number of patients, statistical rationale provided' a blinded data review will be performed to assess the original assumptions for the sample size calculations. This will involve a 'blinded' assessment of the pooled variability of the efficacy data (comprising the raw cognitive variables and overall composite).

At no stage will these data be unblinded and as such, there will be no statistical impact on the final analysis.
Outline details of the objective, variables involved and timing are given in the section called 'Interim Data Review'. These details will be fully described in the Statistical Analysis Plan (SAP), which will contain supporting specifically required tables, figure and listing shells (TF's) for review.

16.1.1 Interim Data Review

A single overall statistical analysis will be performed at the conclusion of the project. No formal interim analyses are planned and therefore no impact on the experiment wise type 1 error rate is envisaged.

However, in order to enable a review and reassessment of the project's power expected at the outset, a 'blinded' re-estimation of the pooled variability will be performed when the DSMB review is completed.
This will involve investigation of the primary cognitive composite of the NTB Z score at week 26 with supporting information provided from each component item.

'Adaptation' in the design of this project will take the form of a potential increase in the total number of patients recruited and analyzed at the conclusion. This will be dependent on the demonstration of a larger variability estimate than that initially planned. The power calculations will then be repeated using the new pooled variability estimate, the sample size will be increased accordingly and the protocol will be amended.

No reduction of the sample size is intended to be done, if the variability estimate is smaller than initially planned.

The DSMB review, which will be performed at the same time, will involve a blinded evaluation of the supporting safety data (particularly adverse events and withdrawals).

16.2 Analysis Populations

Three patient populations will provide the basis for all statistical analyses, data evaluations and summaries included in the investigation.

The primary patient population of interest is the 'Intention to Treat' population (ITT). This will include all patients who receive at least one dose of medication and who subsequently provide any post baseline information. All patients in this group will be analyzed according to the randomization scheduled.

A secondary supporting population will also be investigated. This is defined as the 'Per-Protocol' population (PP) and this will include all patients who satisfactorily complete the 26-week treatment and fully comply with requirements of the protocol regarding the efficacy evaluations. All patients in this group will be analyzed according to the treatment they actually received.

For all safety evaluations, an overall 'Safety' population will also be described. This will include all patients who receive at least one dose of medication and consider all patients according to the treatment they actually received.

16.3 Missing Data

All possible efforts will be made to ensure that patients complete all the required assessments at each study visit and in general there will be no imputation of any missing data for any of the assessments performed.

However, as missing data in a chronic deteriorating condition such as Alzheimer's potentially influences the outcome through the production of biased endpoint estimates the effect of 'missingness' will be investigated in the form sensitivity analyses. These involve re-analysis of the data with substitution of the patient missing value with alternative values. Various replacement strategies may be investigated including: 'Worst case' (using the group worst score), 'Last observation carried forward' (using the last observed value), 'Modified last observation carried forward' (allowing for the rate of deterioration), 'Multiple imputation (using a grouped average value).

The overall 'drop-out' rates in each of the active treatments and placebo groups will also be considered to determine the proportion of data substitution.

These strategies will be applied to appropriate primary and secondary endpoints using ITT patient population.

16.4 Data Analysis

All data analysis and reporting procedures will use SAS v9.2 or higher in a Windows 7 operating system environment.

All data will be summarized according to endpoint or visit, treatment group and overall for each patient population. In addition, visit windows specified will be taken into account (Week 4: +/-7 days, Week 12: +/-7days and Week 26: +/-7days).

All numeric, interval data will be presented as means, standard deviation, minimum, maximum, median, lower quartile, and upper quartile as well as number of observations for both actual values and changes from baseline data. All categorical data will be presented in contingency tables as frequencies and percentages.

16.5 Evaluation of Safety and Tolerability Parameters

As appropriate, listings and summary tables by treatment will be provided for the safety and tolerability assessments. The investigator will evaluate the safety and tolerability of the treatment applied during the studies. This evaluation will take into account the recorded AEs, vital signs, ECG parameters, clinical laboratory assessments, C-SSRS, and any other parameter that is relevant for safety assessments.

16.5.1 Adverse events

Summary tables of TEAEs will be presented by system organ class based on the Medical Dictionary for Regulatory Activities (MedDRA) terminology list (preferred terms): a table containing the number of subjects experiencing the AE by treatment, a table by treatment and relationship, and a table by treatment and severity.

16.5.2 Clinical laboratory test values

Clinical laboratory data will be listed accompanied by an indication if the parameter is outside the reference range, and a summary listing of all data outside the reference range will be prepared.

16.5.3 Vital signs and ECG parameters

Vital signs and ECG parameters will be listed and summarized descriptively.

16.5.4 Physical examination

Any significant physical examination findings will be listed.

16.5.5 Columbia-Suicide Severity Rating Scale

Any changes from baseline will be listed and summarized by treatment.

16.5.6 MRI

MRI scans will be performed according to a MRI protocol at Visit 2-Baseline and Visit 5 End of Treatment. The scans will be analyzed by central reader. All changes from baseline will be listed and summarized by treatment.

16.6 Exploratory/ Efficacy Analyses

Secondary exploratory variables of interest are the individual cognitive and biomarker assessments including:

- Regional brain glucose metabolism (^{18}FDG-PET)
- CSF-Biomarkers (tau, p-tau, Aβ40, Aβ42, AchE activity)
- NTB:
  - Digit Span
  - Category Fluency Test
  - COWAT
  - Digit Symbol Substitution Test (DSST)
- ADAS-cog 13 items

- SPATIAL ORIENTATION AND LEARNING (Amunet)
- Geriatric Depression Scale (GDS)
- Clinical Global Impression Scale – Improvement (CGI-I)

16.6.1 Cognitive Tests

Exploratory efficacy analysis will involve the NTB after normalization (Z score) at endpoint and the change from baseline at 26 weeks will be calculated and analyzed.

NTB consists of the Digit Span Test, Category Fluency Test, the COWAT, and the Digit Symbol Substitution test.

This endpoint will be analyzed for the two populations and the two analysis strategies – ITT, ITT-last observed carried forward (LOCF), ITT-modified last observation carried forward (MLOCF) and PP. The ITT-LOCF will be the primary population of interest.

These data will be analyzed using a fixed effect weighted Analysis Of Covariance (ANCOVA) model including Treatment and Country as main factors and Treatment*Country as an interaction term and Baseline score as covariate. As type III sums of squares will be used throughout the interaction term will be removed from the model in the event of non-significance ($p>0.10$) for the purposes of estimation. Results will be presented as point estimates and associated 95% confidence intervals for the differences between each treatment dose and the placebo group via linear contrasts, which utilize the pooled variance estimator.

Treatment by country interaction will be assessed by examining the results of individual countries and by fitting separate analysis of covariance (ANCOVA) models including a term for treatment by country interaction.

The assumptions of normality and homogeneity of variance will be assessed by inspection of normal probability plots and residual plots. If these assumptions are not met, appropriate non-parametric methods or data transformations will be used.

16.6.2 Other Tests

All other tests are considered to be numeric interval data and will be analyzed as per the NTB composite score, except for GDS and CGI-I which are considered ordered categorical in type.

Ordered categorical endpoints will be analyzed using a Cochran Mantel-Haenszel (CMH-row mean scores difference), chi-squared test or Logistic regression models (as appropriate).

Additional analyses will involve evaluation of the time course of treatment response over the 26 weeks treatment period, using a repeated measures mixed model Analysis Of Covariance (ANCOVA). This will incorporate change from baseline data for each assessment week (4, 12 and 26). This mixed model will include Treatment and Country fitted as fixed effects, Time as a repeated measure effect and Patient as a random effect. The most appropriate covariance pattern between observations on the same subject will be selected. Results will be presented as point estimates and 95% confidence intervals for overall treatment and treatment by visit differences between each treatment dose and the placebo group.

Assessment of Treatment by Country interaction and model fit will be formed using methods analogous to those described for the primary efficacy endpoints. If assumptions are not met, then appropriate alternative methods will be used.

Where appropriate, the biochemical and imaging pharmacodynamic markers will be analyzed as for the primary and secondary clinical efficacy endpoints.

Any additional exploratory analyses will be defined in detail in the analysis plan.

16.6.3 Separate PET Scan Data Analyses

18F-PET Scan variables will be analyzed in a descriptive and exploratory manner separately from the main analysis and this will be performed at Karolinska Institutet, Translational Alzheimer Neurobiology, Center for Alzheimer Research in Stockholm, Sweden by Prof. Agneta Nordberg.

Following attenuation correction and reconstruction to an isotropic image resolution with a filtered back projection, all PET scans will be spatially normalized by registration to a common template. Reliable registration will be facilitated by the incorporation of the corresponding structural MRI scan.

Global intensity (counts) normalization of the PET scans will be done with the intensity in the pons. Assessment of regional glucose metabolism will then be done in template based, predefined regions including the frontal, parietal, occipital and temporal lobe, and the basal ganglia. Regional activity on 18F-FDG-PET scans will be compared to two doses of study medication or placebo using univariate ANOVA with repeated measurements.

16.6.4 Demographic and Baseline Characteristics

Assessments performed prior to randomization, at Screening and Baseline will be displayed using summary statistics at the conclusion of this study. These include: Demographics, Relevant Medical/Psychiatric/Neurological History, Height, Weight and Physical and Neurological Examination. Treatment groups and patient populations will be identified. No formal statistical analyses are planned. All data will be described appropriately as Interval or Categorical summaries.

16.6.5 Diagnostic Criteria

All diagnostic assessments performed at Screening will be summarized at the conclusion of this study. No formal statistical analyses are planned. All data will be described as Interval or Categorical summaries according to treatment group and patient population as appropriate.

16.6.6 Sample Size

Fifty-one (51) patients per group are sufficient to demonstrate an effect size difference of 0.56 between either active dose and placebo control with 80% power and type 1 error rate 0.05 (two tailed).

Allowing for an approximate drop-out rate of 15%, then a grand total of 240 patients (80 patients per group) will be recruited to the study at 20 centers in a ratio of 1:1:1 (200mg bid, 400mg bid, placebo).

16.6.7 Sample Size Justification

In view of the resources available for this investigation, a group sample size of 80 patients may be insufficient to demonstrate convincing and clinically relevant findings.

Therefore, to support these group sizes estimation of the likely magnitude of the precision of the treatment differences has also been assessed.

As treatment estimates obtained are random estimates from a distribution, it is possible to calculate the confidence limit for the resulting treatment estimates and to specify a lowest probable limit (95% confidence actually chosen).

Assuming a pooled standard deviation of 10 and a two-sided 95% confidence margin, then 51 patients per group will in fact enable the precision (lower limit) of the estimated difference in

treatment means observed (x) to be calculated to within 3.9 points (i.e.: Lower confidence margin = x-3.9).

However, in view of the stage of development and therefore limited availability of prior drug use information, a blinded data review will be performed to assess the 'actual' overall pooled variability of the primary efficacy endpoints. If this is larger than 'planned', this information may be used to adapt the trial and to increase the required sample size.

This 'planned' sample size is however considered sufficient to provide reasonably accurate information for the planning and powering of a subsequent efficacy study.

17 ETHICS AND REGULATORY

17.1 Independent Ethics Committee (IEC) or Institutional Review Board (IRB)

The Trial Protocol, any amendment to the protocol that is not solely of an administrative nature, the Investigators' Brochure, the Patient/ Caregiver Information Sheet and Informed Consent Form as well as consent form updates (if applicable) and patient recruitment procedures (if applicable) will be submitted to the responsible Independent Ethics Committee or Institutional Review Board to obtain an approval/favorable opinion according to local regulations.

17.2 Regulatory Authority

Before initiating the clinical trial, the sponsor should submit any required application to the appropriate authority(-ies) for review, acceptance and/or permission (as required by the applicable regulatory requirements) to start the trial. Any notification/submission should be dated and contain sufficient information to identify the protocol. Any amendment or relevant safety information becoming available during the trial will be submitted according to the regulatory requirements.

17.3 Ethical conduct of the study

The study will be conducted in accordance with ethical principles that have their origins in the Declaration of Helsinki, and that are consistent with ICH GCP Guidelines and applicable regulatory requirements.

17.4 Patient information and consent

In compliance with the Declaration of Helsinki and GCP, the patient (or legally acceptable representative) will be given full verbal and written information on the nature, objective, significance, expected benefits, potential risks and consequences of the study by the Investigator or authorized designee prior to inclusion of a patient into the study. The obligations of the Investigator as set forth in the Declaration of Helsinki, the ICH Guideline for Good Clinical Practice and the respective national legislation are to be respected.

During the course of the study, the patient (or legally acceptable representative) and caregiver will be informed in a timely manner if information becomes available that may be relevant to the patient's willingness to continue participation in the study.

The patient and caregiver will be informed that the recorded data will be passed on to authorized persons in an anonymous form. Only for the purpose of checking the validity of the data, authorized persons (*e.g.* Sponsor, person authorized by the Sponsor, auditors, Regulatory Authorities, members of Independent Ethics Committees/Institutional Review Boards), being bound to confidentiality, will have access to non-anonymous data. If the results of the study are published,

the patient's identity will remain confidential. Furthermore, the obligations of the patient/ caregiver and the fact that he/she is free to withdraw the consent at any time without stating the reason are described. However, in case of Adverse Events or poor tolerability, the patient should inform the Investigator. Each patient and caregiver will have the opportunity to discuss the informed consent form with the Investigator or authorized designee prior to giving his/her written consent. The patient will be given a copy of the signed and dated written informed consent form and consent form updates (if applicable).

A statement on the informed consent procedure must be given in the Case Report Forms.

17.5 Insurance

From the beginning of the study until its termination, each patient is insured against any health impairment occurring as a result of participation in the study in accordance with the laws and regulations of the country in which the study is performed.

The patients will be informed by the investigator about the existence of this insurance and the resulting obligations. A copy of the insurance certificate and conditions will be handed over to the patient, if requested. Any medical deviation from this trial protocol caused by patient's fault is not covered by this insurance.

17.6 Data handling and record keeping

All essential documents must be safely retained by the Sponsor and the Investigator for at least two years following the date a marketing application is approved for the drug for the indication for which it is being investigated; or if no application is filed or if the application is not approved for such indication according to local law and requirements of the Sponsor after the investigation is discontinued and the respective Regulatory Authorities are notified. Essential documents at the investigational site include (among other documents):

- Patient list (the investigator receives a template at the beginning of the study) containing patient numbers, patient's name and date of birth. This form is to identify the patients and will only be kept at the study center
- A signed copy of the final Trial Protocol and any amendment
- Signed patient Informed Consent Forms
- A copy of site Investigator's and co-workers' Curricula Vitae
- Copies of all correspondence with the Ethics Committee/IRB and any direct correspondence with the Regulatory Authority
- A copy of relevant laboratory reference ranges and methods
- Copies of study supplies receipt, drug inventory form
- Copies of all correspondence between the Investigator and the Clinical Monitor and between the Investigator and NeuroScios, respectively
- Documentation of handling of IP

Patient files will be archived according to local legislation.

Prior to destruction of the Investigator Site File (ISF), the Investigator needs to obtain permission in writing from NeuroScios or PharmatrophiX.

Authorized staff of the investigators will record the data in the provided study e-CRF, accurately and in a timely manner. Only the Investigator and authorized co-workers are entitled to make entries into the e-CRF and all entries will be made in English.

Data cleaning and processing will be managed through Data Magik Ltd, Laburnum House, East Grimstead, Salisbury, UK, SP5 3RT, Tel: +44 (0)1722 712972 electronic data capture and data management system. The database development process will include the production of an annotated pdf of the e-CRF detailing the variable names used in the database. It is not allowed to pass the assigned password to colleagues. Data Magik will provide a Data Management Plan. Data Management and Data Base Lock will be in accordance with the Data Management Plan.

Data Magik EDC system will be programmed to capture all data in a dedicated database through the specified e-CRF screens in a secure manner. Clinical Data will be recorded directly into an electronic Case Report Form (e-CRF) by assigned site personnel. All data should be recorded as early as possible within the e-CRF.

Access via the internet to the e-CRF is defined through permission-based roles, for example data entry or read-only. All changes to the e-CRF are recorded, along with a reason for change, in the system audit trail. Username, Password and web-address will be provided to study personnel.

Edit checks will be built into the design of the e-CRF screens and global rules programmed into Data Magik's CDMS.

All queries will be resolved at the clinical site.

The final e-CRF will be electronically signed off by the investigator.

18 FINANCING

The financial aspects of the study will be documented in an agreement between the Sponsor/CRO and each Investigator or any other involved party and must be confirmed in writing before the study commences.

19 PUBLICATION POLICY

The results of this study and any discoveries related to this study, regardless if they have technical or medical character are the exclusive properties of the Sponsor. Publications by the Investigator of any information on results of this study must be in accordance with the Clinical Trial Research Agreement.

20 APPENDIX 1 - WORLD MEDICAL ASSOCIATION DECLARATION OF HELSINKI

Ethical Principles for Medical Research Involving Human Subjects

Adopted by the 18th WMA General Assembly, Helsinki, Finland, June 1964.

Applicable Version: 59th WMA General Assembly, Seoul, October 2008

A. INTRODUCTION

1. The World Medical Association (WMA) has developed the Declaration of Helsinki as a statement of ethical principles for medical research involving human subjects, including research on identifiable human material and data. The Declaration is intended to be read as a whole and each of its constituent paragraphs should not be applied without consideration of all other relevant paragraphs.

2. Although the Declaration is addressed primarily to physicians, the WMA encourages other participants in medical research involving human subjects to adopt these principles.

3. It is the duty of the physician to promote and safeguard the health of patients, including those who are involved in medical research. The physician's knowledge and conscience are dedicated to the fulfilment of this duty.

4. The Declaration of Geneva of the WMA binds the physician with the words, "The health of my patient will be my first consideration," and the International Code of Medical Ethics declares that, "A physician shall act in the patient's best interest when providing medical care."

5. Medical progress is based on research that ultimately must include studies involving human subjects. Populations that are underrepresented in medical research should be provided appropriate access to participation in research.

6. In medical research involving human subjects, the well-being of the individual research subject must take precedence over all other interests.

7. The primary purpose of medical research involving human subjects is to understand the causes, development and effects of diseases and improve preventive, diagnostic and therapeutic interventions (methods, procedures and treatments). Even the best current interventions must be evaluated continually through research for their safety, effectiveness, efficiency, accessibility and quality.

8. In medical practice and in medical research, most interventions involve risks and burdens.

9. Medical research is subject to ethical standards that promote respect for all human subjects and protect their health and rights. Some research populations are particularly vulnerable and need special protection. These include those who cannot give or refuse consent for themselves and those who may be vulnerable to coercion or undue influence.

10. Physicians should consider the ethical, legal and regulatory norms and standards for research involving human subjects in their own countries as well as applicable international norms and standards. No national or international ethical, legal or regulatory requirement should reduce or eliminate any of the protections for research subjects set forth in this Declaration.

B. PRINCIPLES FOR ALL MEDICAL RESEARCH

11. It is the duty of physicians who participate in medical research to protect the life, health, dignity, integrity, right to self-determination, privacy, and confidentiality of personal information of research subjects.

12. Medical research involving human subjects must conform to generally accepted scientific principles, be based on a thorough knowledge of the scientific literature, other relevant sources of information, and adequate laboratory and, as appropriate, animal experimentation. The welfare of animals used for research must be respected.

13. Appropriate caution must be exercised in the conduct of medical research that may harm the environment.

14. The design and performance of each research study involving human subjects must be clearly described in a research protocol. The protocol should contain a statement of the ethical considerations involved and should indicate how the principles in this Declaration have been addressed. The protocol should include information regarding funding, sponsors, institutional affiliations, other potential conflicts of interest, incentives for subjects and provisions for treating and/or compensating subjects who are harmed as a consequence of participation in the research study. The protocol should describe arrangements for post-study access by study subjects to interventions identified as beneficial in the study or access to other appropriate care or benefits.

15. The research protocol must be submitted for consideration, comment, guidance and approval to a research ethics committee before the study begins. This committee must be independent of the researcher, the sponsor and any other undue influence. It must take into consideration the laws and regulations of the country or countries in which the research is to be performed as well as applicable international norms and standards but these must not be allowed to reduce or eliminate any of the protections for research subjects set forth in this Declaration. The committee must have the right to monitor ongoing studies. The researcher must provide monitoring information to the committee, especially information about any serious adverse events. No change to the protocol may be made without consideration and approval by the committee.

16. Medical research involving human subjects must be conducted only by individuals with the appropriate scientific training and qualifications. Research on patients or healthy volunteers requires the supervision of a competent and appropriately qualified physician or other health care professional. The responsibility for the protection of research subjects must always rest with the physician or other health care professional and never the research subjects, even though they have given consent.

17. Medical research involving a disadvantaged or vulnerable population or community is only justified if the research is responsive to the health needs and priorities of this population or community and if there is a reasonable likelihood that this population or community stands to benefit from the results of the research.

18. Every medical research study involving human subjects must be preceded by careful assessment of predictable risks and burdens to the individuals and communities involved in the research in comparison with foreseeable benefits to them and to other individuals or communities affected by the condition under investigation.

19. Every clinical trial must be registered in a publicly accessible database before recruitment of the first subject.

20. Physicians may not participate in a research study involving human subjects unless they are confident that the risks involved have been adequately assessed and can be satisfactorily managed.

Physicians must immediately stop a study when the risks are found to outweigh the potential benefits or when there is conclusive proof of positive and beneficial results.

21. Medical research involving human subjects may only be conducted if the importance of the objective outweighs the inherent risks and burdens to the research subjects.

22. Participation by competent individuals as subjects in medical research must be voluntary. Although it may be appropriate to consult family members or community leaders, no competent individual may be enrolled in a research study unless he or she freely agrees.

23. Every precaution must be taken to protect the privacy of research subjects and the confidentiality of their personal information and to minimize the impact of the study on their physical, mental and social integrity.

24. In medical research involving competent human subjects, each potential subject must be adequately informed of the aims, methods, sources of funding, any possible conflicts of interest, institutional affiliations of the researcher, the anticipated benefits and potential risks of the study and the discomfort it may entail, and any other relevant aspects of the study. The potential subject must be informed of the right to refuse to participate in the study or to withdraw consent to participate at any time without reprisal. Special attention should be given to the specific information needs of individual potential subjects as well as to the methods used to deliver the information. After ensuring that the potential subject has understood the information, the physician or another appropriately qualified individual must then seek the potential subject's freely-given informed consent, preferably in writing. If the consent cannot be expressed in writing, the non-written consent must be formally documented and witnessed.

25. For medical research using identifiable human material or data, physicians must normally seek consent for the collection, analysis, storage and/or reuse. There may be situations where consent would be impossible or impractical to obtain for such research or would pose a threat to the validity of the research. In such situations the research may be done only after consideration and approval of a research ethics committee.

26. When seeking informed consent for participation in a research study the physician should be particularly cautious if the potential subject is in a dependent relationship with the physician or may consent under duress. In such situations the informed consent should be sought by an appropriately qualified individual who is completely independent of this relationship.

27. For a potential research subject who is incompetent, the physician must seek informed consent from the legally authorized representative. These individuals must not be included in a research study that has no likelihood of benefit for them unless it is intended to promote the health of the population represented by the potential subject, the research cannot instead be performed with competent persons, and the research entails only minimal risk and minimal burden.

28. When a potential research subject who is deemed incompetent is able to give assent to decisions about participation in research, the physician must seek that assent in addition to the consent of the legally authorized representative. The potential subject's dissent should be respected.

29. Research involving subjects who are physically or mentally incapable of giving consent, for example, unconscious patients, may be done only if the physical or mental condition that prevents giving informed consent is a necessary characteristic of the research population. In such circumstances the physician should seek informed consent from the legally authorized representative. If no such representative is available and if the research cannot be delayed, the study may proceed without informed consent provided that the specific reasons for involving subjects with a condition that renders them unable to give informed consent have been stated in the research protocol and the study has been approved by a research ethics committee. Consent to

remain in the research should be obtained as soon as possible from the subject or a legally authorized representative.

30. Authors, editors and publishers all have ethical obligations with regard to the publication of the results of research. Authors have a duty to make publicly available the results of their research on human subjects and are accountable for the completeness and accuracy of their reports. They should adhere to accepted guidelines for ethical reporting. Negative and inconclusive as well as positive results should be published or otherwise made publicly available. Sources of funding, institutional affiliations and conflicts of interest should be declared in the publication. Reports of research not in accordance with the principles of this Declaration should not be accepted for publication.

21 APPENDIX 2 - ELEMENTS OF INFORMED CONSENT

The following principles should be followed in preparing an informed consent statement.

In compliance with the Declaration of Helsinki, prior to inclusion of the patient into the study, the patient (or legally acceptable representative) will be given full verbal and written information on the patient's diagnosed disease, nature, objective/purpose, significance of the research study, study design and experimental practice of the research study, expected benefits, potential risks and consequences of the study by the Investigator or authorized designee.

The patient will receive the following information orally and written in the informed consent:

- treatments within the research study (dosage, application, duration of treatment for the study drug and placebo), description of the study drug to be administered, expected effects of the study drug and how much experience has been gained on how many patients in previous trials
- explanation of placebo and probability for random assignment to each treatment
- in case of a blinded trial, explanations of emergency unblinding
- statement of the approval status of study drug and approved in which countries worldwide
- expected duration of the patient's participation in the research study, of approximate number of patients involved and how many countries and sites will participate in this research trial
- experimental aspects and invasive procedures performed within the study (including amount of blood samples in mL and respective spoons) and potential risks of these invasive procedures
- detailed study visit schedule
- all patient responsibilities (patient diaries, restrictions in diet or life style)
- alternative treatment possibilities including important potential benefits and risks
- reasonably foreseeable risks (including ineffective treatment and side-effects of the applied compound) or inconveniences to the subject and, if applicable, to an embryo, fetus or nursing infant if the patient may become pregnant and information for reason of monthly pregnancy test, if applicable
- insurance (including the insurance company name, mailing address and policy number) and compensation in the event of trial-related injuries and, if any, anticipated prorated payment to the patient for participating in the research study
- anticipated expenses, if any, to the patient for participating in the research study (including healthcare-related expenses and travel-related expenses)
- statement of the foreseeable circumstances and/or reasons under which the patient's participations in the research study may be terminated
- name(s) and telephone number(s) of a contact person at the investigational site for questions or in case of an emergency.

It is ensured that language in the written Informed Consent is easy to understand. The obligations of the Investigator as set forth in the Declaration of Helsinki, the ICH Guideline E6 (10 Jun 1996) for Good Clinical Practice and the respective national legislation are to be respected.

During the course of the study the patient (or legally acceptable representative) will be informed in a timely manner, if information becomes available that may be relevant to the patient's willingness to continue participation in the study.

The patient (or legally acceptable representative) will be informed that the recorded data will be passed on to authorized persons in a pseudonym form.

Only for the purpose of checking the validity of the data, authorized persons (e.g. Sponsor, person authorized by the Sponsor, auditors, Regulatory Authorities, members of Independent Ethics Committees/Institutional Review Boards), being bound to confidentiality, will have access to the code encrypting the pseudonym form. If the results of the study are published, the patient's identity will remain confidential. Furthermore, the obligations of the patient (or legally acceptable representative) and the fact that participation within the research study is voluntarily and the patient is free to withdraw the consent at any time without stating the reason are described. However, in case of Adverse Events or poor tolerability the patient (or legally acceptable representative) should inform the Investigator. Each patient (or legally acceptable representative) will have the opportunity to discuss the informed consent form with the Investigator or authorized designee prior to giving his/her written consent. The patient (or legally acceptable representative) will be given a copy of the signed and dated written informed consent form and consent form updates (if applicable).

A statement on the informed consent procedure must be given in the Case Report Forms.

22 APPENDIX 3 - MINI MENTAL STATE EXAMINATION (MMSE)

Introduction

Each score able item should be queried. When a year or season is near transition the correct answer can be prompted with „are you sure?“. If a patient gives the date when prompted for day, he/she should be given credit if the response is correct. The year can be prompted with „nineteen...“. Day or season can be prompted with a list of relevant names.

SCORING

No partial credit is given. Acceptable responses for address include street name without number. For hospital any correct name except generic names such as „medical centre“ or „hospital“. All other items require exact answers. Each item worth 1 point.

MINI-MENTAL STATE EXAMINATION (MMSE)

ITEM		Points	Score
**ORIENTATION**			
1.	What is the year?	1	________
2.	What is the season of the year?	1	________
3.	What is the date?	1	________
4.	What is the day of the week?	1	________
5.	What is the month?	1	________
6.	Can you tell me where we are?	1	________
	(For instance, what country are we in?)		
7.	What country are we in?	1	________
8.	What city/town are we in?	1	________
9.	What is the name or address of this place?	1	________
10.	What floor of the building are we on?	1	________
**IMMEDIATE RECALL**			
11.	I am going to name three objects. After I have said them, I want you to repeat them. Repeat what they are because I am going to ask you to name them again in a few minutes.		
	APPLE TABLE PENNY	3	_______

Please repeat the names for me (1 second to name each).

(Give 1 point for each correct answer on the first trial. Count trials.)

Record # of trials: _______________

**ATTENTION AND CALCULATION**		
12. "Now I'd like you to subtract 7 from 100. Then keep subtracting 7 from each answer until I tell you to stop".<br>Then say: "What is 100 take away 7?"<br>Prompt with: "Keep going."<br>You may repeat instructions, but only <u>before</u> the patient begins subtracting.<br>_ _ _ _ _<br>Scoring is assessed on a trial-by-trial basis. Seven less than the previous answer is correct, even if the answer before was incorrect.<br>E.g., 93, 80, 73, 72, 71 – is 2 correct answers	5	

**RECALL**		
„What were the three objects I asked you to remember?"		
13. Apple: ______________________________	3	_______
14. Table: ______________________________		
15. Penny: ______________________________		

**LANGUAGE**		
16. (Show wrist watch) What is this called?<br>_____________	1	_______
17. (Show pencil) What is this called?<br>________________	1	_______
18. I would like you to repeat a phrase after me:<br>(The phrase is): „No if's and's or but's."	1	_______
_ _ _ _ _<br>Allow only one trial.		
19. Read the words on this page, then do what it says. (The paper reads): *„Close your eyes"*<br>_ _ _ _ _<br>Code correct if patient closes eyes.	1	_______
20. I'm going to give you a piece of paper. Criteria: When I do, take the paper in your right hand, fold the	3	_______

paper in half with both hands, and put the paper down on your lap.<br><br>_ _ _ _ _<br><br>Read full statement **<u>then</u>** hand over the paper. **<u>Do not repeat instructions or coach</u>** (score 1 point for each correct step.)	right hand<br><br>folds<br><br>on lap		
21. Write any complete sentence on the piece of paper for me.		1	_______
22. Here is a drawing, please copy the drawing on the same paper. Score correct if the two five-sided figures intersect to form a four-sided figure and if all angles in the five-sided figure are preserved.		1	_______

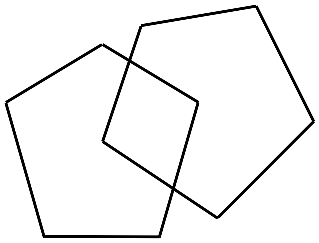

TOTAL POINTS	TOTAL SCORE
30	

24 APPENDIX 4 -DIGIT SPAN TEST

The digit span consists of two levels of difficulty. The digit span forward and the digit span backwards. In the digit span forward task the participant is asked to enter the digits in the given order. In the digit span backward task the participant needs to reverse the order of the numbers that is, by starting with the last number you said and going backwards to the first number you said.

Please instruct the participant at the start of the testing session as follows:
„I am going to say some numbers. Listen carefully, and when I have finished say them right back to me"
Then start right away with the test items. You don´t have to perform a sample item for digit span forward.
Begin with Trial 1 of Item 1. Read the digits at the rate of one per second and then ask the participant to repeat the sequence. Regardless of whether or not the participant repeats this item correctly, present the second series of the same length. So go on to trial 2 item 1. Always administer both trials of each succeeding item, regardless of whether the participant passes the first trial! If the participant passes EITHER trial of Item 1, proceed to Item 2. Discontinue the digit span forward after the participant failed on BOTH trials of any item. Each item is scored 2, 1 or 0:
Score two points if the participant passes both trials/ Score one point if the participant passes only one trial/ Score zero points if the participant fails on both trials.
The maximum score of the digit span forward is 12

**Part 1 Forwards**								
Item	**Trial 1**	Correct Yes	Correct No		**Trial 2**	Correct Yes	Correct No	Scoring (2, 1 or 0) Correct answers
1	6-2-9	☐	☐		3-7-5	☐	☐	
2	5-4-1-7	☐	☐		8-3-9-6	☐	☐	
3	3-6-9-2-5	☐	☐		6-9-4-7-1	☐	☐	
4	9-1-8-4-2-7	☐	☐		6-3-5-4-8-2	☐	☐	
5	1-2-8-5-3-4-6	☐	☐		2-8-1-4-9-7-5	☐	☐	
6	3-8-2-9-5-1-7-4	☐	☐		5-9-1-8-2-6-4-7	☐	☐	
						**SUM**		

For the digit span backwards we have the same procedure but you start with an example item.
Please instruct the participant as follows:

"Now I am going to say some more numbers, but this time when I stop I want you to say them backwards. For example, if I say 2-8-3, what would you say?"

And then pause for the participant to respond. If participant passes the example item continue with the test. If not explain the first example and provide a second example. Please instruct the participant as follows:

"No, I said 2-8-3, so to say them backwards you would need to say 3-8-2. Now try these numbers. Remember you are to say them backwards. Ready? 1-5-8."

Please don´t give any help on this second example or on any of the items that follows. Regardless of whether the participant succeeds or fails with the second example (1-5-8), proceed to Trial 1 of Item 1. After the example item or items (2 trials) administer both trials of item 1 and all succeeding items. Regardless of whether or not the participant repeats this item correctly, present the second series of the same length (Trial 2, Item 1). If the examinee passes EITHER trial of Item 1, proceed to Item 2. Discontinue after failure on BOTH trials of any item.

Scoring Rules are the same as for the Digit Span Forwards Task. The maximum score of the digit span backwards is 12. The maximum TOTAL score for the whole test is 24.

**Part 2 Backwards**								
Item	**Trial 1**	Correct Yes	Correct No	**Trial 2**	Correct Yes	Correct No	Scoring (2, 1 or 0) Correct answers
1	5-1	☐	☐	3-8	☐	☐	
2	4-9-3	☐	☐	5-2-6	☐	☐	
3	3-8-1-4	☐	☐	1-7-9-5	☐	☐	
4	6-2-9-7-2	☐	☐	4-8-5-2-7	☐	☐	
5	7-1-5-2-8-6	☐	☐	8-3-1-9-6-4	☐	☐	
6	4-7-3-9-1-2-8	☐	☐	8-1-2-9-3-6-5	☐	☐	
					**SUM**		

Total Score: ☐

25 APPENDIX 5 – CATEGORY FLUENCY TEST

For the start, when you instruct the test to the patient for the first time, say the following:

„I am going to give you a category and I want you to name, as fast as you can, all the things that belong in that category. For example, if I say „articles of clothing“, you could say shirt, tie or hat. Can you think of other articles of clothing?“

If the patient passes the example-task, continue with the following test-instruction:

„I want you to name all the things that belong to another category that is „animals“. You will have one minute. I want you to tell me all the animals you can think of in one minute. Ready? Begin.“

Press the time watch as you say “begin”. From that time on, the patient has 60 seconds to name animals.

Category: ANIMALS

**Words (1 minute testing time)**	✓

Accepted words (✓)	
Total generated words (W)	

26 APPENDIX 6 - CONTROLLED ORAL WORD ASSOCIATION TEST (COWAT)

The COWAT measures a person's ability to make verbal associations to specified letters (i.e., F, A and S), evaluates the spontaneous production of words beginning with a given letter and is able to detect changes in word association fluency often found with various disorders.

To instruct the patient, say something like:

"I will say a letter of the alphabet. Then I want you to tell me as many words that begin with that letter as quickly as you can. For example, if I say the letter "B", you might say "bad", "bottle", "bed". You should not repeat any words and I do not want you to use words that are proper names such as "Boston" or "Bob". Also, do not use the same word again with a different ending such as "eat" and "eating". You will have one minute. Any questions? Begin when I say the letter. Tell me all the words that you can that start with the letter "F"."

Then the patient has 1 minute to name words with the given letter – every word the patient says needs to be written down, even repetitions or incorrect words. Repeat the task with the letters "A" and "S".

Trial 1_ words with „F"

Words (1 minute testing time)	✓

Accepted words (✓)	
Total generated words (W)	

27 APPENDIX 7 - DIGIT SYMBOL SUBSTITUTION TEST

INSTRUCTIONS

Place the response form in front of the subject. Read aloud the instructions.

"Look at these boxes. Notice that each has a number in the upper part and a special mark in the lower part. Each number has its own mark. Now look down here where the boxes have numbers in the top part but the squares at the bottom are empty (point to the sample items).
"You are to put in each of the empty squares the mark that should go there, like this: Here is a 2; the 2 has this mark, so I put it in this square like this. Here is a 1; the 1 has this mark, so I put it in this square. This number is 3; the 3 has this mark, so I put it in this square (examiner fills in first three boxes to demonstrate).
"Now you fill in the squares up to this heavy line. (If subject makes errors, continue to help until all sample items are filled in correctly.) ***Yes, now do you know how to do them?"***
"When I tell you to start, you do the rest of them. Begin here and fill in as many squares as you can, one after the other, without skipping any. Keep working until I tell you to stop. Work as quickly as you can without making any mistakes. When you finish this line, go on to this one. Ready? Begin."

- Allow 90 seconds for the test.

Coding

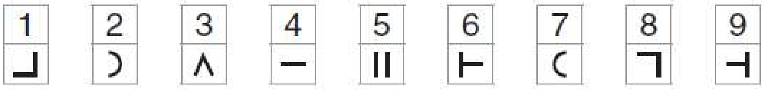

Demo Sample

6	8	3	9	5	4	1	7	2	1	4	8	2	7	6	9	3	5

8	3	1	9	2	5	6	4	3	7	2	9	8	1	4	7	6	5

9	1	2	4	7	2	5	6	9	5	8	6	4	3	1	7	8	3

1	3	9	6	3	9	7	5	1	4	2	8	7	2	8	5	6	4

7	6	4	1	3	2	8	1	7	9	2	5	3	4	8	6	5	9

8	1	9	5	1	4	2	6	9	8	7	3	5	6	4	7	2	3

3	6	8	9	1	8	4	7	5	2	9	6	7	1	5	2	3	4

28 APPENDIX 8 - ALZHEIMER'S DISEASE ASSESSMENT SCALE - COGNITIVE ITEMS

ADAS Cog 13 items (total score = 85);

Administration and Scoring Procedures

The word recall task is administered first followed by the remaining cognitive tasks. Some time is spent in open-ended conversation in order to access various aspects of expressive speech.

The rating scale of 0-5 reflects the degree of severity of dysfunction. A rating of 0 signifies no impairment on a task or absence of a particular behavior. A rating of 5 is reserved for the most severe degree of impairment or very high frequency of occurrence of a behavior. A rating of 1 signifies a very mild presence of a behavior or corresponds to a particular performance level on a task. Ratings of 2, 3, or 4 correspond to mild, moderate, and moderately severe, respectively. Ratings on many cognitive behaviors correspond to levels of performance on tasks.

1. **Word-recall task.**

On this task the patient is given three trials to learn a list of ten high-frequency *(Throndike & Lorge, 1944),* high-imagery *(Pavio et al., 1968)* nouns. The ten words are printed in block letters on cards. At the start of the first trial the tester gives instructions similar to the following: "I am going to show you some words one at a time. Please read each word out loud and try to remember it because later I will ask you to try to remember all of the words I have shown you." After the presentation the tester asks the patient to try to recall as many words as possible. Two more learning and recall trials follow. The patient's score is the mean number of words not recalled on three trials (maximum 10). Multiple forms of this task, using different sets of words equated for frequency and imagery, should be used if the patient is to be retested with less than 6 months between test sessions.

2. **Commands**

This task is designed to assess receptive speech. The patient is asked to carry out 1 to 5 step commands *(Goodglass & Kaplan, 1972)*. Each command should be read once. If the patient does not respond or makes an error, the tester should then give the entire command one more time. Then go on to the next command. All commands should be given to every patient.

1. Make a <u>fist</u>.
2. Point to the <u>ceiling</u> and then to the <u>floor</u>. Line up a pencil, watch, and card, in that order, on a table in front of the patient.
3. Put the pencil on top of the card and then put it back.
4. Put the <u>watch</u> on the <u>other side</u> of the <u>pencil</u> and then <u>turn over</u> the card.
5. Tap each shoulder twice, with two fingers, keeping your eyes shut.

Each underlined element represents a single step. Each command scored is as a whole.

0 = All commands correct

1 = 1 command incorrect, 4 commands correct

2 = 2 commands incorrect, 3 commands correct

3 = 3 commands incorrect, 2 commands correct

4 = 4 commands incorrect, 1 command correct

5 = All 5 commands incorrect

3. Constructional praxis

This task assesses the patient's ability to copy four geometric forms ranging from a very simple one, a circle, to a fairly difficult one, a cube. The forms should be presented one at a time. Each form is presented on a Rating Book page. The tester should give the patient a pall pen along with the drawing. The instructions to the patient should be: "On this piece of paper is a shape. Try to draw another one that looks just like this somewhere on the page." The patient should be allowed two attempts for each shape. If the patient cannot reproduce the figure in two attempts the tester should go on to the next item. The forms, in the order of presentation, are:

1. Circle approximately 2.0 cm in diameter.
2. Two overlapping rectangles, one oriented vertically and one oriented horizontally.
3. Diamond with the long axis oriented vertically.
4. Cube with each side approximately 2.0 cm and all internal lines visible.

Each form should be located in the upper middle of a plain sheet of white paper. A drawing should be scored as correct if the patient has reproduced all of the essential geometric features of the original. Changes in size do not count as errors. Small gaps between lines do not indicate an error as long as the shape has been reproduced. Scoring criteria for each form (examples shown below*):

1. Circle. A closed curved figure.
2. Two overlapping rectangles. Forms must be four-sided and overlap must be similar to presented form. Changes in size are not scored.
3. Diamond. Figure must be four-sided, oriented so that points are at the top and bottom, and the sides' approximately equal length.
4. Cube. The form is three-dimensional, with front face in the correct orientation, internal lines drawn correctly between corners. Opposite sides of faces should be approximately parallel.

*2. Correct

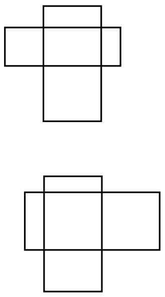

Incorrect

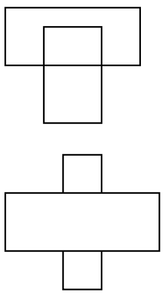

*3. Correct

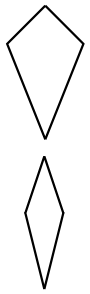

Incorrect

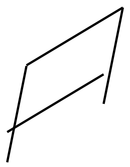

*4. Correct

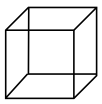

Incorrect

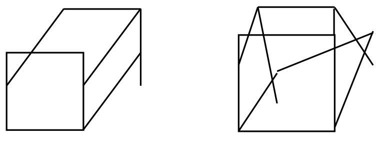

The scoring rule for this item is:

0 = All 4 drawings correct

1 = 1 form drawn incorrectly

2 = 2 forms drawn incorrectly

3 = 3 forms drawn incorrectly

4 = 4 forms drawn incorrectly

5 = No figures drawn at all or no figures drawn correctly (scribbles, parts of forms or words instead of forms)

4. Delayed Word-Recall task

On this task the patient should recall as many words as possible from the Immediate Word-Recall task. The ten words are printed in block letters on cards. At the start of the first trial the tester gives instructions similar to the following: "Few minutes ago I had you read some words printed on these Cards. (point to word list) Tell me all of the words you can remember that were on the cards."

The patient's score is the number of words not recalled.

5. Naming objects and fingers

For this item the patient is asked to name 12 randomly presented objects printed on picture cards, whose frequency values *(Throndike & Lorge, 1944)* are: high, medium, and low. The patient is also asked to name the fingers of his/her dominant hand, i.e. thumb, pinky, index (pointer, forefinger), middle and ring fingers. The first question about each object should be "What is this called?" or "What is the name of this thing?". If the patient does not respond, then the examiner should give the cue for that item listed below. If the patient still doesn't respond or makes an error, go on to the next object. For many of the objects there is more than one correct response. A response other than the name given below should be scored as correct if it is a name that would be used by a no demented person with the same cultural background as the patient. For example, the mask might be called a "false face" in some parts of the U.S., the wallet might be called a "billfold" or the harmonica might be called a "mouth organ". Descriptions of the object, semantic or phonemic paraphasias should not be scored as correct. Examples of incorrect responses are "listening thing" for stethoscope, "cutter" for scissors and "prongs" for "tongs". The objects, their frequency and the clues are:

High Frequency:

- Flower (artificial) – grows in the garden
- Bed (doll house furniture) – used for sleeping
- Whistle – makes a sound when you blow on it
- Pencil – used for writing

Medium Frequency:

- Rattle – a baby's toy
- Mask – hides your face
- Scissors – cuts paper
- Comb – used on hair

Low Frequency:

- Wallet – holds your money

- Harmonica – a musical instrument
- Stethoscope – doctor uses it to listen to your heart
- Tongs – picks up food

The scoring rule for this item is:

0 = 0-2 items named incorrectly
1 = 3-5 items named incorrectly
2 = 6-8 items named incorrectly
3 = 9-11 items named incorrectly
4 = 12-14 items named incorrectly
5 = 15-17 items named incorrectly

6. Ideational praxis

This task is designed to determine whether the patient can perform a familiar but complex sequence of actions. The patient is given sheet of paper, a long envelope and a pencil. The tester should give the patient instructions similar to the following: "I want you to pretend that you have written yourself or to a family member or a friend a letter. Take this piece of paper, fold it so that it will fit into the envelope, and then put it into the envelope. Then seal the envelope, address the envelope to yourself, and show me where the stamp goes." There are five components to this task and each one is underlined in the instruction. If the patient forgets part of the task, or is having difficulty, the tester should repeat the instruction for the component of the task where the patient is having difficulty. For example, if the patient stops after folding the paper and putting it into the envelope, the tester should give one reminder on the next component, "Now seal the envelope". If the patient cannot do this part move on and give one reminder on the next component, "Now address the letter to yourself or to a family member or to a friend". After the first complete instruction only one additional reminder should be given for each component. Impairment on this item should reflect dysfunction in executing an over learned task only and not recall difficulty. Note that any address which would enable a postal worker to deliver the envelope is counted as correct even though it might not be the patient's current address. The address should contain: name, street, city, and state.

To score this item, simply count up the number of components that the patient failed to perform correctly:

0 = All components performed correctly
1 = Failure to perform 1 component
2 = Failure to perform 2 components
3 = Failure to perform 3 components
4 = Failure to perform 4 components
5 = Failure to perform 5 components

7. Orientation.

The components of orientation are: person, day of the week, date, month, year, season, time of day, place. The tester should ask the patient for each of these pieces of information one at a time. Before giving this item the tester should be sure that no clocks, watches or calendars are visible to aid the patient. One point is given for each incorrect response (maximum = 8). Acceptable answers include: ± 1 for the date; within one hour for the hour; partial name for place; naming of upcoming season with two weeks prior to its onset; and name of previous season for two weeks after its termination. Month, year, day of the week and the person's first and last name must be exact.

8. Word-recognition task.

On this task the patient is given three trials to learn a list of 12 words. The learning part of each trial is similar to the learning part of the word recall task since the patient is asked to read each word aloud and try to remember it. For each of the three test trials the 12 studied words are mixed with 12 new words matched to the studied words for frequency and imagery and the patient is asked to decide for each word whether or not it was one of the studied words. At the start of the first trial the tester gives instructions to the patient similar to the following: “I am going to show you some words printed on cards. I want you to read each word out loud and try to remember it.” Some of the words on the word recognition task may not be familiar to the patient and the patient may have difficulty reading them. If the patient cannot read a word, the tester should say the word out loud. However, it is important for the patient to actually look at each word and try to read it. At the end of the learning portion of a trial the tester should say to the patient something like the following: “Now I’m going to show you another set of words. Some of the words were on the list I just showed you and others are new. For each word I want you to tell me whether it is one of the words I just showed you.” Then the tester shows the first word and says either “Is this one of the words I showed you before, yes or no?” or “Did I show you this word before?” The same instruction is given before the second test word. For the remaining test words the tester should say: “How about this one?” If the patient does not remember the task (e.g. reads the word rather than responding yes or no) then the tester should repeat or rephrase the entire question and make a note that the patient had to be reminded of the task instructions.

Trials two and three are similar, and the tester should keep track of the number of times the patient has to be reminded of the test instructions.

To score this item count the number of incorrect responses on each trial, but allow only a maximum error score of 12 for each trial. Since the probability of guessing a correct response is ½ for each word, the average number of errors by a person guessing randomly will be 12. Thus, a person with no memory for any of the studied words would make an average of 12 errors per trial if they simply guessed for every test word. An error score of greater than 12 must be due to factors other than poor memory such as unlucky guessing. The total score for the item is the mean number of incorrect responses for three trials. Multiple forms of this task, with different words equated for frequency and imagery, should be used if the patient is to be retested with less than 6 months between test sessions.

9. Remembering test instructions

This item evaluates the patient’s ability to remember the requirements of the recognition task. On each recognition study, the patient is asked prior to presentation of the first two words, “Did I show you this word before or is this a new word?” For the third word, the patient is asked, “How about this one?” If the patient responds appropriately, i.e. “yes” or “no”, then memory for the instructions is accurate. If the patient fails to respond, this signifies that the instructions have been forgotten and the instruction is repeated. The procedure used for the third word is repeated for words 4-24. Each instance of memory failure for the test instructions is noted.

0 = Patient never needs extra reminders of instructions
1 = Very mild - forgets once
2 = Mild - must be reminded 2 times
3 = Moderate - must be reminded 3 or 4 times
4 = Moderately severe - must be reminded 5 or 6 times
5 = Severe - must be reminded 7 or more times

10. Spoken language ability.

This item is a global rating of the quality of speech, i.e., clarity, difficulty in making oneself understood. In rating this item, the tester should consider all of the speech produced by the patient during the test session. Quantity of speech and word finding difficulty are not rated on this item. It should be noted that the higher scores (4-5) on this item are reserved for patients whose expressive language abilities are impaired to such an extent that they seldom communicate without difficulty.

0 = No instances where it is difficult to understand the patient
1 = Very mild - 1 instance of lack of understandability
2 = Mild - subject has difficulty less than 25% of time
3 = Moderate - subject has difficulty 25% to 50% of the time
4 = Moderately severe - subject has difficulty more than 50% of the time
5 = Severe - 1 or 2 word utterances; fluent, but empty speech; mute

11. Word-finding difficulty in spontaneous speech

Along with item 9, this item rates impairment in expressive speech, but it rates only word finding difficulty, whereas item 9 is a more global rating of the extent to which the patient can communicate verbally. To rate this item, the tester must determine whether the patient has difficulty in finding the desired word in spontaneous speech. The problem may be overcome by circumlocution, i.e., giving explanatory phrases or nearly satisfactory synonyms. Do not include finger and object naming this rating.

0 = No evidence of word finding difficulty in spontaneous speech
1 = Very mild - 1 or 2 instances, not clinically significant
2 = Mild - noticeable circumlocution or synonym substitution
3 = Moderate - loss of words without compensation on occasion
4 = Moderately severe - frequent loss of words without compensation
5 = Severe - nearly total loss of content words; speech sounds empty; 1-2 word

12. Comprehension

This item evaluates the patient's ability to understand speech. To rate this item, the tester should consider how well the patient was able to understand the tester's speech during the opening discussion, during the test session and, if applicable, the administration of the no cognitive items. Do not include responses to commands.

0 = No evidence of poor comprehension
1 = Very mild - 1 instance of misunderstanding
2 = Mild - 3-5 instances of misunderstanding
3 = Moderate - requires several repetitions and rephrasing
4 = Moderately severe - patient only occasionally responds correctly; i.e. yes-no
5 = Severe – patient rarely responds to questions appropriately, not due to poverty of speech

13. Concentration/ Distractibility

This item rates the frequency with which the subject is distracted by irrelevant stimuli and/ or must be reoriented to the ongoing task because of loss of train of thought or being caught up in his/ her own thoughts.

0 = No evidence of poor concentration or distractibility
1 = Very mild - 1 instance of poor concentration
2 = Mild - 2-3 instances of poor concentration/ distractibility; signs of restlessness and inattentiveness
3 = Moderate - 4-5 instances during interview
4 = Moderately severe - poor concentration/ distractibility throughout much of the interview
5 = Severe – extreme difficulty in concentration and extremely distractible, unable to complete tasks

29 APPENDIX 9 - GERIATRIC DEPRESSION SCALE 15-ITEM - SHORT VERSION

Suitable as a screening test for depressive symptoms in the elderly. Ideal for evaluating the clinical severity of depression, and therefore for monitoring treatment. It is easy to administer, needs no prior psychiatric knowledge and has been well validated in many environments - home and clinical.

The original GDS was a 30 item questionnaire - time consuming and challenging for some patients (and staff). Later versions retain only the most discriminating questions; their validity approaches that of the original form. The most common version in general geriatric practice is the 15-item version.

INSTRUCTIONS

Undertake the test orally. Obtain a clear "yes" or "no" answer. If necessary, repeat the question. Cross off either "yes" or "no" for each question (depressive answers are bold/italicized). Count up 1 for each depressive answer.

Geriatric Depression Scale - Short Form

Choose the best answer for how you have felt over the past week:

1. Are you basically satisfied with your life?	YES	NO
2. Have you dropped many of your activities and interests?	YES	NO
3. Do you feel that your life is empty?	YES	NO
4. Do you often get bored?	YES	NO
5. Are you in good spirits most of the time?	YES	NO
6. Are you afraid that something bad is going to happen to you?	YES	NO
7. Do you feel happy most of the time?	YES	NO
8. Do you often feel helpless?	YES	NO
9. Do you prefer to stay at home rather than going out and doing new things?	YES	NO
10. Do you feel you have more problems with memory than most?	YES	NO
11. Do you think it is wonderful to be alive now?	YES	NO
12. Do you feel pretty worthless the way you are now?	YES	NO
13. Do you feel full of energy?	YES	NO
14. Do you feel that your situation is hopeless?	YES	NO
15. Do you think that most people are better off than you are?	YES	NO

Answers marked in grey indicate a depression.

Scoring:

Assign one point for each of these answers:

1.NO	4. YES	7. NO	10. YES	13. NO
2.YES	5. NO	8. YES	11. NO	14. YES
3.YES	6. YES	9. YES	12. YES	15. YES

A score of 0 to 5 is normal. A score above suggests depression.

30 APPENDIX 10 - SPATIAL ORIENTATION AND LEARNING (AMUNET)

What are we testing?

AlloEgo Navigation – The capability of spatial navigation both with the aid of visible orientation cues that are not placed near the goal (allocentric navigation) and with the aid of information about the direction and the distance from the start that is needed to find the goal (egocentric navigation).

Ego Navigation – Focused on egocentric navigation.

Allo Navigation – Focused on allocentric navigation.

Delayed Navigation – Focused on delayed allocentric navigation.

Managing the Program

To open the test, go to Amunet.exe and choose Amunet 1.

The following list includes the function of each of the keys used in this test:

- Press the Enter to start / stop measurement of pointer's / mouse movement and show orienting cues
- Press the right arrow to move to the next trial / experiment
- Press the left arrow to move to the previous trial
- Press "**r**" to start / stop the rotation of the image in the Demo part of the test
- Press the ESC to terminate the experiment
- Press the Spacebar to show / hide the goal
- Press "**c**" to show / hide the orienting cues
- Press "**s**" to show / hide the start

Using the Manual

Black Letters – General test instructions and instructions on what you need to do are written in black letters.

Blue Letters – verbatim instructions for the participants during the test are written in blue letters.

Test Instructions

Demo

Press on "Demo" with the pointer/ mouse and then press on "Start test" to start the Demo part of the test, which will demonstrate AlloEgo Navigation. The following picture will appear on the screen:

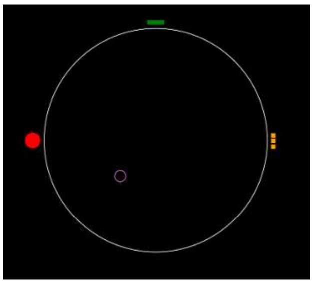

Then say to the examinee: This white large circle is the arena and represents the map view of the field in which you will be traveling (show it to the participant). The red dot on the edge of the arena

is the start (point to the red dot), the small purple hollow circle inside of the arena is the goal (point to the purple hollow circle), and the yellow and green lines on the edge of the arena are the orienting cues (point to both the yellow and the green lines).

Your task is to find the goal. You will need to begin at the start and, if possible, trace a path to the goal via the shortest route. However, even though I will show you the goal at the beginning, you will need to remember where it is located because it will disappear from the screen and it will not be visible. The start and the two orienting cues (point to the orienting lines) will help you find the goal.

You can imagine that the start (point to the start), the goal (point to the goal) and the yellow and green orienting cues (point to the cues) create an irregular quadrilateral shape (point to the shape). This shape will stay the same throughout the rest of this task. Although the mutual relationship of the start, the goal, and the orienting cues will remain the same, the whole image will rotate by a small amount like this (press "r" to start the rotation of the image, let it rotate for a few seconds and then press "r" again to stop the rotation). Do you understand this task? (if the examinee does not understand, explain it again). Press twice the right arrow to end the trial and to go back to the AMUNET start screen.

2 – **AlloEgo Virtual**

Press on "AlloEgo Virtual" with the pointer/ mouse and then press on "Start test" to start the AlloEgo Virtual part of the test. The AlloEgo part of the test will demonstrate AlloEgo Navigation.

The following picture will appear on the screen:

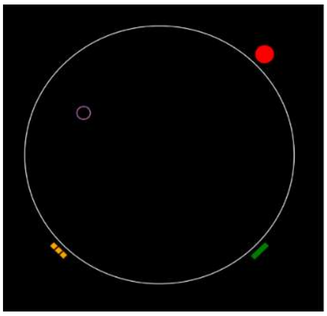

Then say to the examinee: Let's now do the task. Just like before, the large white circle is the arena. The red dot on the edge of the circle is the start (point to the red dot), the small purple hollow circle inside the arena is the goal (point to the purple circle), and the yellow and green lines are the orienting cues (point to the yellow and green lines).

Your task will be to begin at the start and trace a path in a straight line to the goal. Remember how I said that the goal will disappear from the arena and your task will be to find it with the help of the starting point (point to the red dot) and the two orienting cues (point to the yellow and green lines)? Memorize now how far and in which direction the goal is from the start (point to the distance and

Touch the start with the tip of your pointer (let the examinee touch the red dot with the pointer and instruct to do so if they don't automatically touch it). When the goal becomes invisible, trace a path to where the hidden goal is. It is not important how quickly you do the task, but the precision with which you find the goal. Press the Enter to start the first trial and to show the orienting cues. The goal is still the same distance and direction from the start and from the yellow and green orienting cues. Tell me where the hidden goal is.

Let the examinee trace a path from start to goal and once they tell you where the goal is, press the Enter to find the goal. The position of the goal will appear again and whether or not the examinee finds the goal, give him/her feedback and repeat the instructions: **Again, look at the correct position of the goal and memorize how far and in which direction the goal is from the start** (point to the distance and direction between the goal and the start), **from the green orientation**

cue (point again) **and from the yellow orientation cue** (point again). Press the right arrow for next trial.

Repeat this 8 more times. There are a total of 9 trials in the AlloEgo phase.

The order of the keys is – the Enter, the Enter, and the right arrow.

For all subsequent trials in this task, state the following before each trial:

Show me again where the goal is. It is still the same distance and direction from the start and from the yellow and green orienting cues.

Similarly, state the following after each trial:

Look again at the correct position of the goal and try to remember how far and in which direction it is from start and from the yellow and green orienting cues.

Ego Virtual

Say to the examinee: Let's now do another task.

Press on "Ego Virtual" with the pointer/ mouse and then press on "Start test" to start the Ego Virtual part of the test, which will demonstrate Egocentric Navigation. The following picture will appear on the screen:

Say to the examinee: Your next task is similar to the previous task and you will again be asked to move the pointer from the start to the goal. However, as there will not be any orienting cues at the edge of the arena, you will need to orient yourself with the help of the start position (point to the red dot). The goal (point to the hollow purple circle) will be located the same distance and direction from the start as in the previous task.

Before each trial you say:

Start here (point to the start position and instruct the examinee to touch the screen with the pointer) **and trace a path from start to the goal** (press the Enter)**. Tell me when you have found the hidden goal.** Press the Enter again to mark the position of the hidden goal and to show the examinee where the position of the goal is. Provide feedback and say the following after each trial: **Look again at the correct position of the goal, and try to remember how far and in which direction it is from the start.** Press the right arrow for next trial.

This task consists of **8** trials. The order of the keys is the same as in the previous task the Enter, the Enter, and the right arrow.

Allo Virtual

Say to the participant: Let's now do a different task.

Press on "Allo Virtual" with the pointer/ mouse and then press on "Start test" to start the Allo Virtual part of the test, which will demonstrate Allocentric Navigation. The following picture will appear on the screen:

Say to the participant: In the previous task, you were able to orient yourself with the help of the start position, however, in this next task, the start will appear randomly. The yellow and green orienting cues will also appear on the screen and will play an important role – you can use the

yellow and green cues to help orient yourself and find the goal. The relationship between the goal and the orienting cues will stay the same.

Try now to remember how far and in which direction the small hollow purple circle was placed the last time from these yellow and green orienting cues (press the Enter to show the yellow and green lines). Again go to the start and use your pointer to trace a path from start to where you think the hidden goal is, but this time, use the orienting cues to find the goal, do not orient yourself with the start position.

Before each trial you say:

Now you start here (point to the start position and instruct the examinee to touch the screen with the pointer). Try to trace the path again to where the goal is. It is still the same distance and direction from the orienting cues (press the Enter). Tell me when you have found the hidden goal. Press the Enter again to mark the position of the hidden goal and to show the examinee where the position of the goal is. Provide feedback and say the following after each trial: Look again at the correct position of the goal, and try to remember how far and in which direction it is from the yellow and green orienting cues. Press the right arrow for next trial.

This task consists of **8** trials. The order of the keys is the same as in the previous task – the Enter, the Enter, and the right arrow.

Before finishing say to the participant: Remember how far and in which direction the goal is from the yellow and green orienting cues because I will be asking you later.

Delayed

Say to the participant: Now, let's get back to the navigation task.

Press on "Delayed" with the pointer/ mouse and then press on "Start test" to start the Delayed part of the test, which will demonstrate Delayed memory recall and Allocentric Navigation. The following picture will appear on the screen:

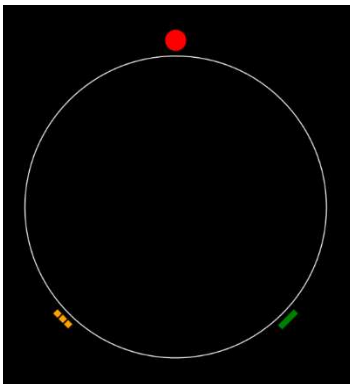

Say to the participant: Your task here again is to find the goal, while you are only guided by the orienting cues, and not by the start. Try to remember how far and in which direction the goal was placed from these yellow and green lines (press the Enter to show the orienting cues). I will not be able to show you the correct position of the goal. Tell me when you are at the hidden goal position (when the participant indicates that they are at the goal, press the Enter). Repeat this one more time.

31 APPENDIX 11 - CLINICAL GLOBAL IMPRESSION – SEVERITY/IMPROVEMENT

CGI - Severity:

Assess the overall severity of patient's illness (Visit 1).

1 = Normal—not at all ill, symptoms of disorder not present past seven days
2 = Borderline mentally ill—subtle or suspected pathology
3 = Mildly ill—clearly established symptoms with minimal, if any, distress or difficulty in social and occupational function
4 = Moderately ill—overt symptoms causing noticeable, but modest, functional impairment or distress; symptom level may warrant medication
5 = Markedly ill—intrusive symptoms that distinctly impair social/occupational function or cause intrusive levels of distress
6 = Severely ill—disruptive pathology, behavior and function are frequently influenced by symptoms, may require assistance from others
7 = Among the most extremely ill patients—pathology drastically interferes in many life functions; may be hospitalized

CGI - Improvement:

Assess the overall change in the severity of patient's illness, compared to start of clinical trial (Visit 1).

1 = Very much improved—nearly all better; good level of functioning; minimal symptoms; represents a very substantial change
2 = Much improved—notably better with significant reduction of symptoms; increase in the level of functioning but some symptoms remain
3 = Minimally improved—slightly better with little or no clinically meaningful reduction of symptoms. Represents very little change in basic clinical status, level of care, or functional capacity
4 = No change—symptoms remain essentially unchanged
5 = Minimally worse—slightly worse but may not be clinically meaningful; may represent very little change in basic clinical status or functional capacity
6 = Much worse—clinically significant increase in symptoms and diminished functioning
7 = Very much worse—severe exacerbation of symptoms and loss of functioning

32 APPENDIX 12 - THE MODIFIED HACHINSKI ISCHEMIC SCALE

The Modified Hachinski Ischemic Scale is used to assess the presence of vascular risk factors in patients with dementia. The scale consists of 8 items and has a maximum score of 12, with higher scores indicative for vascular dementia. In this study, only patients with a MHIS score equal to or less than 4 will be enrolled.

A score of less than or equal to 4 is necessary to rule out a diagnosis of Multi-Infarct Dementia.

	Point value if present	Score
1. Abrupt onset	2	
2. Stepwise deterioration	1	
3. Somatic complaints	1	
4. Emotional incontinence	1	
5. History or presence of hypertension	1	
6. History of stroke	2	
7. Focal neurological symptoms	2	
8. Focal neurological signs	2	
	Total	

33 APPENDIX 13 - C-SSRS

Introduction

The Columbia–Suicide Severity Rating Scale (C-SSRS) is an assessment tool that evaluates suicidal ideation and behavior. This guide outlines the proposed safety outcomes and statistical analysis strategy for the C-SSRS for an individual clinical trial.

As noted in the August 2012 draft guidance titled "Suicidal Ideation and Behavior: Prospective Assessment of Occurrence in Clinical Trials", the FDA has adopted the 11 categories defined in the C-SSRS (five subtypes of suicidal ideation, five subtypes of suicidal behavior, and self-injurious behavior without suicidal intent) as their standard. Some data collected on the C-SSRS are not included in these proposed displays for safety analysis (e.g., suicidal behavior lethality and suicidal ideation intensity), but are used for individual clinical management, safety monitoring, or other research purposes.

Outcomes

There is current debate on whether suicidal ideation and suicidal behavior should be combined and analyzed as a single outcome. Researchers agree on the need for having analyses that keep suicidal ideation and suicidal behavior separate, but disagree on whether there is value to having any outcome that combines them. This document currently includes the combined outcomes measure.

The following outcomes are C-SSRS categories and have binary responses (yes/no). The categories have been re-ordered from the actual scale to facilitate the definitions of the composite and comparative endpoints, and to enable clarity in the presentation of the results.

- Category 1 – Wish to be Dead
- Category 2 – Non-specific Active Suicidal Thoughts
- Category 3 – Active Suicidal Ideation with Any Methods (Not Plan) without Intent to Act
- Category 4 – Active Suicidal Ideation with Some Intent to Act, without Specific Plan
- Category 5 – Active Suicidal Ideation with Specific Plan and Intent
- Category 6 – Reparatory Acts or Behavior
- Category 7 – Aborted Attempt Category 8 – Interrupted Attempt
- Category 9 – Actual Attempt (non-fatal) Category 10 – Completed Suicide

Endpoints

Composite endpoints based on the above categories are defined below.

- Suicidal ideation: A "yes" answer at any time during treatment to any one of the five suicidal ideation questions (Categories 1-5) on the C-SSRS.
- Suicidal behavior: A "yes" answer at any time during treatment to any one of the five suicidal behavior questions (Categories 6-10) on the C-SSRS.
- Suicidal ideation or behavior: A "yes" answer at any time during treatment to any one of the ten suicidal ideation and behavior questions (Categories 1-10) on the C-SSRS.
- Comparative endpoints of interest are defined below. "Treatment emergence" is used for outcomes that include events that first emerge or worsen. "Emergence" is used for outcomes that include events that first emerge.

Number of Patients with Suicidal Ideation, Suicidal Behavior, and Self- Injurious Behavior without Suicidal Intent Based on the C-SSRS During Treatment;

Events during treatment (percentages)	Drug Name<br>N=xx<br>n (%)	Comparator Name<br>N=xx<br>n (%)	p-values[a] (to compare percentages)
Suicidal Ideation (1-5)			0.xxx
1) Wish to be dead	x (%)	x (%)	
2) Non-specific active suicidal thoughts	x (%)	x (%)	
3) Active suicidal ideation with any methods (not plan) without intent to act	x (%)	x (%)	
4) Active suicidal ideation with some intent to act, without specific plan	x (%)	x (%)	
5) Active suicidal ideation with specific plan and intent	x (%)	x (%)	
Suicidal Behavior (6-10)			0.xxx
6) Preparatory acts or behavior	x (%)	x (%)	
7) Aborted attempt	x (%)	x (%)	
8) Interrupted attempt	x (%)	x (%)	
9) Non-fatal suicide attempt	x (%)	x (%)	
10) Completed suicide	x (%)	x (%)	
Suicidal Ideation or Behavior (1-10)	x (%)	x (%)	0.xxx
Self-injurious behavior without suicidal intent	x (%)	x (%)	

NOTE: the p-value column should be eliminated when inferential analyses are not performed depending on the Sponsor's standard approach for safety data presentations.

[a]p-values are from [specify test].

Notes: N = number of enrolled patients with at least one post-baseline C-SSRS assessment. In this table, n and (%) refer to the number and percent of patients who experience the event at least once during treatment. For the composite endpoint of suicidal ideation (1-5), n and (%) refer to the number and percent of patients who experience any one of the five suicidal ideation events at least once during treatment. For the composite endpoint of suicidal behavior (6-10), n and (%) refer to the number and percent of patients who experience any one of the five suicidal behavior events at least once during treatment. For the composite endpoint of suicidal ideation or behavior (1-10), n and (%) refer to the number and percent of patients who experience any one of the ten suicidal ideation or behavior events at least once during treatment.

34 APPENDIX 14 - ADMINISTRATIVE MATTERS

34.1 Responsibilities of the Investigator

- To ensure that he/she has sufficient time to conduct and complete the study and has adequate staff and appropriate facilities which are available for the duration of the study and to ensure that other studies do not divert essential patients or facilities away from the study at hand.
- To submit an up-to-date curriculum vitae and other credentials (e.g. medical license) to PharmatrophiX and, where required, to relevant regulatory authorities.
- Preparing and maintaining adequate case histories designed to record observations and other data pertinent to the study.
- Following and complying with Good Clinical Practice guidelines.
- Verifying adequate training of personnel administering efficacy instruments at his/her site.
- Ensuring that proper informed consent is obtained. Communicating with their local IRBs.
- Properly documenting all patient related events in the source data

34.2 Protocol Amendments

No modification to the study protocol will be allowed unless discussed in detail with the Sponsor or Delegate and filed as an amendment to this protocol.

Any modifications to the protocol will be adhered to by the participating site (or all participating sites) and will apply to all patients following approval by the Institutional Review Board.

34.3 Sponsor's Termination of Study

PharmatrophiX reserves the right to discontinue the clinical study at any time for medical or administrative reasons. When feasible, a 30-day written notification will be tendered.

34.4 Electronic Case Report Form Instructions

When a patient completes a visit, it is anticipated that all relevant e-CRF pages will be completed within 24 hours of the availability of the last data, but in any case no more than 5 days later. These directions also apply to forms for potential study participants who were not randomized to a treatment group.

As soon as the patient has completed/withdrawn from the study and the e-CRF is completed, the principal Investigator or designated Investigator(s) under his/her supervision will sign the study conclusion page of the e-CRF to confirm that they have reviewed the data and that the data are completed and accurate.

Any questions or comments related to the e-CRF should be directed to the assigned study monitor.

34.5 Monitoring

Monitoring visits by a professional representative of PharmatrophiX or its designee will be scheduled to take place before entry of the first patient, during the study at appropriate intervals and after the last patient is completed.

These visits are for the purpose of verifying adherence to the protocol and the completeness and exactness of data entered on the e-CRF and Drug Inventory Forms. The monitor will verify CRF entries by comparing them with the hospital/clinic/office records which will be made available for

this purpose. The monitor will review completed e-CRF sections at each visit. Adequate time and space for these visits must be made available by the Investigator.

The Investigator must ensure provision of reasonable space and adequate qualified personnel for monitoring visits.

34.6 Archiving of Data

The Investigator must retain patient records as well as drug disposition records until disposal has been agreed upon in writing with PharmatrophiX. The Investigator must have a “key“linking the patient’s study identification number (e.g., randomization number) with the patient’s clinical file. If the Investigator moves or retires, he/she must nominate someone in writing to be responsible for record keeping. Archived data may be held on microfiche or electronic record, provided that a back-up exists and a hard copy can be obtained from it, if required.

PharmatrophiX agrees to retain a copy of the protocol, documentation, approvals and all other documents related to the study, including certificates that satisfactory audit and inspection procedures have been carried out.

34.7 Audits

For the purpose of compliance with Good Clinical Practice it may be necessary for PharmatrophiX, its designee, or authority to conduct a site audit. This may occur at any time from start to after conclusion of the study.

When an Investigator signs the protocol, he agrees to allow auditors to inspect his/her study records. Furthermore, if an Investigator refuses an inspection, his data would not be accepted in support of a New Drug Application.

35 REFERENCES

1. Nguyen TV, Shen L, Vander Griend L, Quach LN, Belichenko NP, Saw N, Yang T, Shamloo M, Wyss-Coray T, Massa SM, Longo FM. Small Molecule P75ntr Ligands Reduce Pathological Phosphorylation and Misfolding of Tau, Inflammatory Changes, Cholinergic Degeneration, and Cognitive Deficits in Abetapp(L/S) Transgenic Mice. J Alzheimers Dis. 2014;42(2):459-83. doi: 10.3233/JAD-140036. PubMed PMID: 24898660; PMCID: 4278429.

2. Massa SM, Xie Y, Yang T, Harrington AW, Kim ML, Yoon SO, Kraemer R, Moore LA, Hempstead BL, Longo FM. Small, Nonpeptide P75ntr Ligands Induce Survival Signaling and Inhibit Prongf-Induced Death. J Neurosci. 2006;26(20):5288-300. doi: 10.1523/JNEUROSCI.3547-05.2006. PubMed PMID: 16707781.

3. Knowles JK, Simmons DA, Nguyen TV, Vander Griend L, Xie Y, Zhang H, Yang T, Pollak J, Chang T, Arancio O, Buckwalter MS, Wyss-Coray T, Massa SM, Longo FM. Small Molecule P75ntr Ligand Prevents Cognitive Deficits and Neurite Degeneration in an Alzheimer's Mouse Model. Neurobiol Aging. 2013;34(8):2052-63. doi: 10.1016/j.neurobiolaging.2013.02.015. PubMed PMID: 23545424.

4. Knowles JK, Rajadas J, Nguyen TV, Yang T, LeMieux MC, Vander Griend L, Ishikawa C, Massa SM, Wyss-Coray T, Longo FM. The P75 Neurotrophin Receptor Promotes Amyloid-Beta(1-42)-Induced Neuritic Dystrophy in Vitro and in Vivo. J Neurosci. 2009;29(34):10627-37. doi: 10.1523/JNEUROSCI.0620-09.2009. PubMed PMID: 19710315; PMCID: 2771439.

5. Yang T, Knowles JK, Lu Q, Zhang H, Arancio O, Moore LA, Chang T, Wang Q, Andreasson K, Rajadas J, Fuller GG, Xie Y, Massa SM, Longo FM. Small Molecule, Non-Peptide P75 Ligands Inhibit Abeta-Induced Neurodegeneration and Synaptic Impairment. PLoS One. 2008;3(11):e3604. doi: 10.1371/journal.pone.0003604. PubMed PMID: 18978948; PMCID: 2575383.

6. Simmons DA, Knowles JK, Belichenko NP, Banerjee G, Finkle C, Massa SM, Longo FM. A Small Molecule P75ntr Ligand, Lm11a-31, Reverses Cholinergic Neurite Dystrophy in Alzheimer's Disease Mouse Models with Mid- to Late-Stage Disease Progression. PLoS One. 2014;9(8):e102136. doi: 10.1371/journal.pone.0102136. PubMed PMID: 25153701; PMCID: PMC4143160.

7. Dickens AM, Vainio S, Marjamaki P, Johansson J, Lehtiniemi P, Rokka J, Rinne J, Solin O, Haaparanta-Solin M, Jones PA, Trigg W, Anthony DC, Airas L. Detection of Microglial Activation in an Acute Model of Neuroinflammation Using Pet and Radiotracers 11c-(R)-Pk11195 and 18f-Ge-180. J Nucl Med. 2014;55(3):466-72. doi: 10.2967/jnumed.113.125625. PubMed PMID: 24516258.

8. James ML, Belichenko NP, Nguyen TV, Andrews LE, Ding Z, Liu H, Bodapati D, Arksey N, Shen B, Cheng Z, Wyss-Coray T, Gambhir SS, Longo FM, Chin FT. Pet Imaging of Translocator Protein (18kda) in a Mouse Model of Alzheimer's Disease Using 18f-Pbr06. J Nucl Med. 2015. doi: 10.2967/jnumed.114.141648. PubMed PMID: 25613536.

9. McKhann GM, Knopman DS, Chertkow H, Hyman BT, Jack CR, Jr., Kawas CH, Klunk WE, Koroshetz WJ, Manly JJ, Mayeux R, Mohs RC, Morris JC, Rossor MN, Scheltens P, Carrillo MC, Thies B, Weintraub S, Phelps CH. The Diagnosis of Dementia Due to Alzheimer's Disease: Recommendations from the National Institute on Aging-Alzheimer's Association Workgroups on Diagnostic Guidelines for Alzheimer's Disease. Alzheimers Dement. 2011;7(3):263-9. Epub 2011/04/26. doi: 10.1016/j.jalz.2011.03.005. PubMed PMID: 21514250; PMCID: 3312024.

10. Shi J, Longo FM, Massa SM. A Small Molecule P75(Ntr) Ligand Protects Neurogenesis after Traumatic Brain Injury. Stem Cells. 2013;31(11):2561-74. doi: 10.1002/stem.1516. PubMed PMID: 23940017.

11. Tep C, Lim TH, Ko PO, Getahun S, Ryu JC, Goettl VM, Massa SM, Basso M, Longo FM, Yoon SO. Oral Administration of a Small Molecule Targeted to Block Prongf Binding to P75 Promotes Myelin Sparing and Functional Recovery after Spinal Cord Injury. J Neurosci. 2013;33(2):397-410. doi: 10.1523/JNEUROSCI.0399-12.2013. PubMed PMID: 23303920; PMCID: PMC3710149.

12. Friesland A, Weng Z, Duenas M, Massa SM, Longo FM, Lu Q. Amelioration of Cisplatin-Induced Experimental Peripheral Neuropathy by a Small Molecule Targeting P75 Ntr. Neurotoxicology. 2014;45:81-90. doi: 10.1016/j.neuro.2014.09.005. PubMed PMID: 25277379; PMCID: PMC4268328.

13. Meeker RB, Poulton W, Clary G, Schriver M, Longo FM. Novel P75 Neurotrophin Receptor Ligand Stabilizes Neuronal Calcium, Preserves Mitochondrial Movement and Protects against Hiv Associated Neuropathogenesis. Exp Neurol. 2016;275 Pt 1:182-98. doi: 10.1016/j.expneurol.2015.09.012. PubMed PMID: 26424436; PMCID: PMC4688079.

14. Mosconi L, Berti V, Glodzik L, Pupi A, De Santi S, de Leon MJ. Pre-Clinical Detection of Alzheimer's Disease Using Fdg-Pet, with or without Amyloid Imaging. J Alzheimers Dis. 2010;20(3):843-54. doi: 10.3233/JAD-2010-091504. PubMed PMID: 20182025; PMCID: 3038340.

15. Berti V, Osorio RS, Mosconi L, Li Y, De Santi S, de Leon MJ. Early Detection of Alzheimer's Disease with Pet Imaging. Neurodegener Dis. 2010;7(1-3):131-5. doi: 10.1159/000289222. PubMed PMID: 20197691; PMCID: 3214828.

16. Minoshima S, Giordani B, Berent S, Frey KA, Foster NL, Kuhl DE. Metabolic Reduction in the Posterior Cingulate Cortex in Very Early Alzheimer's Disease. Ann Neurol. 1997;42(1):85-94. doi: 10.1002/ana.410420114. PubMed PMID: 9225689.

17. Monacelli AM, Cushman LA, Kavcic V, Duffy CJ. Spatial Disorientation in Alzheimer's Disease: The Remembrance of Things Passed. Neurology. 2003;61(11):1491-7. PubMed PMID: 14663030.

18. Kalova E, Vlcek K, Jarolimova E, Bures J. Allothetic Orientation and Sequential Ordering of Places Is Impaired in Early Stages of Alzheimer's Disease: Corresponding Results in Real Space Tests and Computer Tests. Behav Brain Res. 2005;159(2):175-86. doi: 10.1016/j.bbr.2004.10.016. PubMed PMID: 15817181.

19. Laczo J, Vlcek K, Vyhnalek M, Vajnerova O, Ort M, Holmerova I, Tolar M, Andel R, Bojar M, Hort J. Spatial Navigation Testing Discriminates Two Types of Amnestic Mild Cognitive Impairment. Behav Brain Res. 2009;202(2):252-9. doi: 10.1016/j.bbr.2009.03.041. PubMed PMID: 19463709.

20. Laczo J, Andel R, Vyhnalek M, Vlcek K, Magerova H, Varjassyova A, Tolar M, Hort J. Human Analogue of the Morris Water Maze for Testing Subjects at Risk of Alzheimer's Disease. Neurodegener Dis. 2010;7(1-3):148-52. doi: 10.1159/000289226. PubMed PMID: 20197695.

21. Gazova I, Vlcek K, Laczo J, Nedelska Z, Hyncicova E, Mokrisova I, Sheardova K, Hort J. Spatial Navigation-a Unique Window into Physiological and Pathological Aging. Front Aging Neurosci. 2012;4:16. doi: 10.3389/fnagi.2012.00016. PubMed PMID: 22737124; PMCID: 3380196.

22. Bohbot VD, Kalina M, Stepankova K, Spackova N, Petrides M, Nadel L. Spatial Memory Deficits in Patients with Lesions to the Right Hippocampus and to the Right Parahippocampal Cortex. Neuropsychologia. 1998;36(11):1217-38. PubMed PMID: 9842767.

23. Braak H, Braak E. Neuropathological Stageing of Alzheimer-Related Changes. Acta Neuropathol. 1991;82(4):239-59. PubMed PMID: 1759558.

24. Hort J, Andel R, Mokrisova I, Gazova I, Amlerova J, Valis M, Coulson EJ, Harrison J, Windisch M, Laczo J. Effect of Donepezil in Alzheimer Disease Can Be Measured by a Computerized Human Analog of the Morris Water Maze. Neurodegener Dis. 2014;13(2-3):192-6. doi: 10.1159/000355517. PubMed PMID: 24192578.

25. Busner J, Targum SD. The Clinical Global Impressions Scale: Applying a Research Tool in Clinical Practice. Psychiatry (Edgmont). 2007;4(7):28-37. PubMed PMID: 20526405; PMCID: 2880930.

26. Folstein MF, Folstein SE, McHugh PR. "Mini-Mental State". A Practical Method for Grading the Cognitive State of Patients for the Clinician. J Psychiatr Res. 1975;12(3):189-98. PubMed PMID: 1202204.

27. Moroney JT, Bagiella E, Desmond DW, Hachinski VC, Molsa PK, Gustafson L, Brun A, Fischer P, Erkinjuntti T, Rosen W, Paik MC, Tatemichi TK. Meta-Analysis of the Hachinski Ischemic Score in Pathologically Verified Dementias. Neurology. 1997;49(4):1096-105. PubMed PMID: 9339696.

Extended imaging methods

MRI/PET Acquisition

An MRI scanning session for the clinical trial consisted of the collection of up to six scan types: an axial T2-weighted scan, an axial fluid attenuated inversion recovery (FLAIR) scan, an axial T2* weighted scan, a sagittal T1-weighted 3D scan and a diffusion tensor imaging (DTI) scan (optional). MRI scans were acquired at 1.5 or 3 Tesla depending on the scanner available at the study site. T2-weighted and FLAIR scans were collected to assess white matter changes and to verify whether the participants met the trial inclusion criteria. T2* weighted images were collected to assess microbleeds. T1-weighted scans were collected to assess brain atrophy and DTI data was collected to assess microstructural tissue changes.

On the same day as the MRI, a static period ^{18}F-FDG PET scan was acquired as a measure of cerebral glucose metabolism.

Participants who had not had an MRI scan within six-months of the start of the study were scanned at the screening visit. All other participants were scanned at study baseline. Participants were additionally scanned at their 26-week or early discontinuation visit (Extended Data Table 2). After scan acquisition, MRI scans were reviewed by experts in the trial's Image Analysis Unit to assess image quality. Scans could be rejected if they deviated from the trial's scan parameters, had significant motion or other artifacts, were incomplete or had unidentified data. Rejected scans were repeated within three weeks of the assessment. Quality control of PET data was performed at post-processing stages and is discussed later.

MRI Pre-processing

Participants with longitudinal sMRI data (n = 206) were analyzed as part of the exploratory outcome analysis of sMRI data. Preprocessing of all neuroimaging data was performed in SPM12 (https://www.fil.ion.ucl.ac.uk/spm/software/spm12/). The first step of image pre-processing was ensuring that all participants' images had approximately the same orientation. To do so, the origin of each image was manually set to the anterior commissure. Each image was then co-registered and resliced to a standard T1-weighted image of an older adult brain to ensure all images had the same matrix dimensions across participants. Next, we performed longitudinal registration with the serial longitudinal pipeline in SPM12 [1]. The midpoint average images were segmented in the Computational Anatomy Toolbox (CAT12) [2] using tissue probability maps which improve classification of subcortical grey matter [3]. The grey and white matter segments resulting from segmentation were used in combination with geodesic shooting to create a custom, age-appropriate population template to improve spatial normalization [4]. A deformation field was estimated between the template and the midpoint average image for each participant.

Prior to spatial normalization, the grey matter segment of each participant's midpoint average image was multiplied by the Jacobian determinant for each timepoint to produce longitudinally modulated grey matter segments which provide an estimate of within subject change over time [1]. Lastly, each participant's deformation field was then applied to each modulated grey matter segment to warp them into the template space for comparison across individuals.

PET Pre-Processing

As with sMRI analyses, only participants with longitudinal PET data (n = 197) were analyzed as part of the exploratory outcome analyses. The origin of ^{18}F-FDG PET images was set to the anterior commissure. PET images for each subject were aligned across timepoints, then coregistered and re-sliced to the subject's T1 weighted midpoint average image. After co-registration to the sMRI data, sMRI-defined deformation fields were used to warp the PET images

into the trial population template space. Next, ^{18}F-FDG PET scans were normalized to the mean uptake within a previously defined spared region of interest to produce standardized uptake value ratio (SUVr) images [5].

After sMRI and PET pre-processing was completed, the sMRI and ^{18}F-FDG PET SUVr images for each participant were smoothed with a 6mm isotropic smoothing kernel.

Quality Control of Neuroimaging Data

MRI and PET data underwent automated and manual quality control checks prior to inclusion in statistical analyses. For sMRI images, CAT12 provided an Image Quality Rating (IQR) for each image, which reflects the level of bias, signal to noise ratio and the resolution of the scan [2]. Images with a CAT12 IQR below 70% were flagged for manual inspection. Additionally, we examined potential outliers in each of the MRI and PET data using the CAT12 check covariance function on spatially normalized grey matter segments and SUVr images. Data with covariance two or more standard deviations below the mean covariance across participants were manually checked for quality. In the sMRI data, a total of 13 participants were excluded after manual inspection due to concerns about segmentation and normalization accuracy, leaving 193 participants to be included in the statistical analysis. In the PET data, a total of 14 participants were excluded. Of these participants, ten had PET images which failed the manual inspection, three participants were excluded because their MRI data failed to segment and their PET data could therefore not be spatially normalized, and one participant did not have MRI data. As a result, 183 participants were included in the PET statistical analyses.

Code to perform longitudinal sMRI and PET pre-processing are available on GitHub (https://github.com/hayleyshanks/Longitudinal-MRI-PET-preproc).

References

1. Ashburner J. Symmetric diffeomorphic modeling of longitudinal structural MRI. *Front Neurosci*. 2013;6. doi:10.3389/fnins.2012.00197

2. Gaser C, Dahnke R, Thompson PM, Kurth F, Luders E, Initiative ADN. CAT – A Computational Anatomy Toolbox for the Analysis of Structural MRI Data. Published online June 13, 2022:2022.06.11.495736. doi:10.1101/2022.06.11.495736

3. Lorio S, Fresard S, Adaszewski S, et al. New tissue priors for improved automated classification of subcortical brain structures on MRI. *NeuroImage*. 2016;130:157-166. doi:10.1016/j.neuroimage.2016.01.062

4. Ashburner J, Friston KJ. Diffeomorphic registration using geodesic shooting and Gauss–Newton optimisation. *NeuroImage*. 2011;55(3):954-967. doi:10.1016/j.neuroimage.2010.12.049

5. Chen K, Langbaum JBS, Fleisher AS, et al. Twelve-month metabolic declines in probable Alzheimer's disease and amnestic mild cognitive impairment assessed using an empirically pre-defined statistical region-of-interest: Findings from the Alzheimer's Disease Neuroimaging Initiative. *NeuroImage*. 2010;51(2):654-664. doi:10.1016/j.neuroimage.2010.02.064
