# Supplementary material for: p75 neurotrophin receptor modulation in mild to moderate Alzheimer disease: a randomized, placebo-controlled phase 2a trial
Source: Nat Med. 2024 May 17;30(6):1761–70. doi: 10.1038/s41591-024-02977-w (PMC11186782; doi:10.1038/s41591-024-02977-w)
Supplement: Supplementary file 2 — Reporting Summary [file 41591_2024_2977_MOESM2_ESM.pdf]

nature portfolio

Corresponding author(s): Hayley Shanks

Last updated by author(s): Mar 27, 2024

Reporting Summary

Nature Portfolio wishes to improve the reproducibility of the work that we publish. This form provides structure for consistency and transparency in reporting. For further information on Nature Portfolio policies, see our Editorial Policies and the Editorial Policy Checklist.

Statistics

For all statistical analyses, confirm that the following items are present in the figure legend, table legend, main text, or Methods section.

n/a	Confirmed	
☐	☒	The exact sample size (*n*) for each experimental group/condition, given as a discrete number and unit of measurement
☒	☐	A statement on whether measurements were taken from distinct samples or whether the same sample was measured repeatedly
☐	☒	The statistical test(s) used AND whether they are one- or two-sided<br>*Only common tests should be described solely by name; describe more complex techniques in the Methods section.*
☐	☒	A description of all covariates tested
☐	☒	A description of any assumptions or corrections, such as tests of normality and adjustment for multiple comparisons
☐	☒	A full description of the statistical parameters including central tendency (e.g. means) or other basic estimates (e.g. regression coefficient) AND variation (e.g. standard deviation) or associated estimates of uncertainty (e.g. confidence intervals)
☐	☒	For null hypothesis testing, the test statistic (e.g. *F*, *t*, *r*) with confidence intervals, effect sizes, degrees of freedom and *P* value noted<br>*Give P values as exact values whenever suitable.*
☒	☐	For Bayesian analysis, information on the choice of priors and Markov chain Monte Carlo settings
☒	☐	For hierarchical and complex designs, identification of the appropriate level for tests and full reporting of outcomes
☒	☐	Estimates of effect sizes (e.g. Cohen's *d*, Pearson's *r*), indicating how they were calculated

Our web collection on statistics for biologists contains articles on many of the points above.

Software and code

Policy information about availability of computer code

Data collection	SAS code (SAS v9.2 or above) was used to perform participant randomization.
Data analysis	Pre-processing of MRI (e.g. segmentation, normalization) and PET (SUVR, spatial normalization) was performed in SPM12 (https://www.fil.ion.ucl.ac.uk/spm/software/spm12/ version 7771) and CAT12 version 12.8.1. Detailed descriptions are available in Supplementary Note 1. Statistical analyses of cognitive and CSF endpoints were performed in MATLAB r2021b and R version 4.2.2

For manuscripts utilizing custom algorithms or software that are central to the research but not yet described in published literature, software must be made available to editors and reviewers. We strongly encourage code deposition in a community repository (e.g. GitHub). See the Nature Portfolio guidelines for submitting code & software for further information.

Data

Policy information about availability of data

All manuscripts must include a data availability statement. This statement should provide the following information, where applicable:

- Accession codes, unique identifiers, or web links for publicly available datasets
- A description of any restrictions on data availability
- For clinical datasets or third party data, please ensure that the statement adheres to our policy

Data files containing pseudonymized participant data (baseline characteristics, raw data used to conduct primary and exploratory endpoint analyses reported in this article) can be shared in compliance with current data protection regulations by the European Union. All requests for data access should be directed to the

corresponding authors. Requests for data will be reviewed and responded to within a one-month period. Data can be shared through data use agreements for research/academic purposes only.

Human research participants

Policy information about studies involving human research participants and Sex and Gender in Research.

Reporting on sex and gender	Given that sample sizes are small due to the exploratory nature of the trial, sex and gender were not explicitly investigated in this report. The proportion of males and females did not differ between the treatment groups (p = 0.74; Table 1). Biological sex was self reported by participants.
Population characteristics	Groups: Placebo, 200mg LM11A-31 twice daily, 400mg LM11A-31 twice daily<br>Median age: 72 years.<br>Race: 100% white.<br>Disease stage: mild to moderate Alzheimer's disease<br>See Table 1 for further participant information
Recruitment	Potential study participants were recruited through participating trial sites and were evaluated by an independent professional (neurologist or psychiatrist) to determine their ability to provide informed consent. If the individual was deemed capable of providing informed consent, a conversation between the potential participant, the physician, and optionally their caregiver, was initiated. During this conversation, subjects were provided the informed consent form for the study and were given time to review this the form. The informed consent form was approved by the trial's ethics committee prior to study commencement. A small proportion of the trial centres made advertisements in newspapers regarding the trial. All advertisement text was approved by the relevant ethics committee. Following informed consent by both the patient and the caregiver, the Screening visit was performed.<br>Potential recruitment biases include biases incurred by the subject pool at the clinic. Additionally, the participant pool in this study is reflective of white participants recruited across clinics in Europe. Further studies will be required to characterize effects of LM11A-31 in diverse participant populations.<br>Study participants were reimbursed for travel costs. There was no financial incentive for participation.
Ethics oversight	The trial was initiated at 21 sites located in five European countries: Austria, the Czech Republic, Germany, Spain, and Sweden. The trial was conducted in accordance with the Declaration of Helsinki and ICH-GCP. All required study documents were submitted to the Ethics Committees of the participating countries. Each of the five countries involved in the study had a lead site, and the Institutional Review Board (IRB) at the lead sites provided ethical approval for the study. The IRBs approving the trial were IRB00002556 (Austria), IRB00002091 (Czech Republic), IRB00007525 (Germany), IRB00004959 (Sweden), IRB00002590 (Spain). The Principal Investigators of the lead trial sites were: Dr. Anne Börjesson-Hanson (Sweden; trial Coordinating Investigator), Dr. Reinhold Schmidt (Austria), Prof. Dr. Jakub Hort (Czech Republic), Dr. Oliver Peters (Germany) and Dr. Rafael Blesa González (Spain).

Note that full information on the approval of the study protocol must also be provided in the manuscript.

Field-specific reporting

Please select the one below that is the best fit for your research. If you are not sure, read the appropriate sections before making your selection.

☒ Life sciences ☐ Behavioural & social sciences ☐ Ecological, evolutionary & environmental sciences

For a reference copy of the document with all sections, see nature.com/documents/nr-reporting-summary-flat.pdf

Life sciences study design

All studies must disclose on these points even when the disclosure is negative.

Sample size	Before the study began, sample size was determined using power calculations that assumed a pooled standard deviation of 10 and a two-sided 95% confidence margin. These analyses determined that 51 participants per group were required to demonstrate an effect size of 0.56 between either dose of LM11A-31 and placebo with 80% power and type 1 error rate of 0.05 (two tailed), resulting in an initial participant target of 60 subjects per arm for a total of 180 subjects. A blinded review of the neuropsychological test battery (NTB) z-score cognitive data from an initial 81 enrolled subjects was performed to assess overall pooled variability across the treatment groups. The pooled variability of the NTB was higher than expected and the sample size target was therefore increased to 80 subjects per arm (240 participants). This was the maximum possible number of participants allowable based on the amount of study medication available.
Data exclusions	1. Failure to perform screening or baseline examinations<br>2. Hospitalization or change of chronic concomitant medication one month prior to screening or during screening period<br>3. Clinical, laboratory or neuro-imaging findings consistent with:<br>•Other primary degenerative dementia, (dementia with Lewy bodies, fronto-temporal dementia, Huntington's disease, Creutzfeldt-Jakob Disease, Down's syndrome, etc.)<br>• Other neurodegenerative condition (Parkinson's disease, amyotrophic lateral sclerosis, etc.)<br>• Cerebrovascular disease (major infarct, one strategic or multiple lacunar infarcts, extensive white matter lesions > one quarter of the total white matter)<br>• Other central nervous system diseases (severe head trauma, tumors, subdural hematoma or other space occupying processes, etc.)

• Seizure disorder
• Other infectious, metabolic or systemic diseases affecting central nervous system (syphilis, present hypothyroidism, present vitamin B12 or folate deficiency, serum electrolytes out of normal range, juvenile onset diabetes mellitus, etc.)
4. A current DSM-IV diagnosis of active major depression, schizophrenia or bipolar disorder
5. Clinically significant, advanced or unstable disease that may interfere with primary or secondary variable evaluations, and which may bias the assessment of the clinical or mental status of the patient or put the patient at special risk, such as:
• chronic liver disease, liver function test abnormalities or other signs of hepatic insufficiency (ALT, AST, Gamma GT, alkaline phosphatase > 2.5 ULN)
• Respiratory insufficiency
• Renal insufficiency (serum creatinine >2mg/dl) or creatinine clearance ≤ 30 mL/min according to Cockcroft-Gault formula). In case of creatinine clearance ≤30 mL/min, an alternative verification of the renal function must be completed using Cystatin C analysis. In case of normal level of Cystatin C, the patient can be included
• Heart disease (myocardial infarction, unstable angina, heart failure, Cardiomyopathy within six months before screening)
• Bradycardia (heart beat <50/min.) or tachycardia (heart beat >95/min.)
• For Austria, Germany, Spain and Sweden: Hypertension (>180/95) or hypotension (<90/60) requiring treatment with more than three drugs
• For Czech Republic: Hypertension (>160/95) or hypotension (<90/60) requiring treatment with more than three drugs
AV block (type II / Mobitz II and type III), congenital long QT syndrome, sinus node dysfunction or prolonged QTcB-interval (males >450 and females >470 msec)
• Uncontrolled diabetes defined by HbA1c >8.5
• Malignancies within the last five years except skin malignancies (other than melanoma) or indolent prostate cancer
• Metastases
6. Disability that may prevent the patient from completing all study requirements (e.g. blindness, deafness, severe language difficulty, etc.)
7. Women who are fertile and of childbearing potential
8. Chronic daily drug intake of ≥ 14 days or expected for ≥ 14 days:
• benzodiazepines (except lorazepam ≤ 1mg for sleeping disorders only), neuroleptics or major sedatives
• Antiepileptics
• Centrally active anti-hypertensive drugs (clonidine, l-methyl DOPA, guanidine, guanfacine, etc.)
• Opioid containing analgesics
9. Nootropic drugs (except Ginkgo Biloba)
10. Austria, Germany, Spain and Sweden: Suspected or known drug or alcohol abuse, i.e. more than approximately 60 g alcohol (approximately 1 liter of beer or 500 ml of wine) per day, indicated by elevated MCV significantly above normal value at screening.
Czech Republic: Suspected or known drug or alcohol abuse, i.e. more than approximately 20 g alcohol per day for females (500 ml of beer or 250 ml of wine) and 30g alcohol per day for males (approximately 750 ml of beer or 375 ml of wine) indicated by elevated MCV significantly above normal value at screening.
11. Suspected or known allergy to any components of the study treatments
12. Enrollment in another investigational study or intake of investigational drug within the previous three months
13. Any condition, which, in the opinion of the investigator, makes the patient unsuitable for inclusion
14. If patient is in any way dependent on the sponsor or the principal investigator or if the patient is accommodated in an establishment on judicial or administrative order
For analyses of primary endpoint data, all participants in the safety population (N = 242) were included in analyses. For secondary and exploratory endpoint data, quality control and outlier detection methods were performed as described in the Methods and Supplementary Note 2.

Replication	The phase 2a trial has not been replicated in an independent cohort of AD patients.
Randomization	Participants were randomized 1:1:1 into placebo, 200mg LM11A-31 or 400mg LM11A-31. The randomization list was developed by Data Magik Ltd and was structured to allow for a total of at least 240 patients (80 per group) with treatment centre as the only stratification variable. A total of 242 patients were finally randomized and treated in the safety population.
Blinding	The sponsor's personnel, study sites' personnel, participants, and caregivers were blinded to the assigned treatment.

Reporting for specific materials, systems and methods

We require information from authors about some types of materials, experimental systems and methods used in many studies. Here, indicate whether each material, system or method listed is relevant to your study. If you are not sure if a list item applies to your research, read the appropriate section before selecting a response.

Materials & experimental systems

n/a	Involved in the study	
☐	☒	Antibodies
☒	☐	Eukaryotic cell lines
☒	☐	Palaeontology and archaeology
☒	☐	Animals and other organisms
☐	☒	Clinical data
☒	☐	Dual use research of concern

Methods

n/a	Involved in the study	
☒	☐	ChIP-seq
☒	☐	Flow cytometry
☐	☒	MRI-based neuroimaging

Antibodies

Antibodies used

CSF samples were analyzed for the core AD CSF biomarkers Aβ42, Aβ40, total tau (t-tau) and phosphorylated tau at position 181 (p-tau181) using the Lumipulse technology1, on a G1200 instrument. The following kits (name, catalogue #) were used: Lumipulse G β-Amyloid 1-42 #230336, Lumipulse G β-Amyloid 1-40 #231524, Lumipulse G Total Tau Ag #30312, and Lumipulse G pTau 181 #230350, all from Fujirebio Europe, Ghent, Belgium. The pre-synaptic proteins SNAP25 and SYT1 were measured using immunoprecipitation mass spectrometry (IP-MS) as described previously in detail2,3, using the SMI-81 mouse monoclonal for SNAP-25 (Nordic Biosite, Täby, Sweden) for the IP having the epitope at the N-terminal (acetylated) Ac 2-11 amino acids of SNAP-25, while the mouse monoclonal antibody clone 41.1 (Synaptic Systems) recognizing the N-terminal 1st calcium-binding domain of SYT1. CSF levels of the post-synaptic protein neurogranin were measured by an in-house ELISA method4, in which the mouse monoclonal antibody Ng36 (epitope Ng63–75) at a final concentration of 0.5 µg/mL (100 µL/well), was used as a capturing antibody, while biotinylated Ng2 (epitope Ng52–63), final concentration 0.5 µg/mL (100 µL/well), was used as detection antibody 5. CSF sTREM2 concentrations were measured using an in-house Meso Scale Discovery (MSD) immunoassay with streptavidin coated plates (Cat#: L45SA, MSD, Rockville, MD, USA), biotinylated goat polyclonal IgG antibody (Cat#: BAF1828, R&D Systems, Minneapolis, MN, USA) as capture, a mouse monoclonal IgG antibody (Cat#: sc-373828, Santa Cruz Biotechnology, Dallas, TX, USA) as secondary, and a SULFO-TAG–labeled goat polyclonal anti-mouse antibody (Cat#: R32AC, MSD, Rockville, MD, USA) for detection, as previously described 6,7. The CSF level of YKL40 was measured using a commercially available assay (Cat#: DC3L10, R&D Systems, Minneapolis, MN, USA) according to the manufacturer's instructions, using a dilution factor of 1:100 for the samples. CSF NFL was measured using an in-house ELISA method8 based on the mouse monoclonal antibodies NFL21 (final concentration 0.5 µg/ml, 100 µl/well) and NFL23 (final concentration 0.5 µg/ml, 100 µl/well), both having the core domain of human NFL as the epitope. CSF acetylcholinesterase activity using an in-house enzymatic Ellman assay9, as also described elsewhere in detail10. All CSF analyses were performed by board-certified laboratory technicians using methods validated for clinical trials. CSF samples from baseline and end-of-study were analyzed side-by-side to reduced possible variability. All analyses were performed blinded to the clinical information. See Supplementary Table 2 for further details on CSF antibodies.

1. Gobom, J. et al. Validation of the LUMIPULSE automated immunoassay for the measurement of core AD biomarkers in cerebrospinal fluid. Clin Chem Lab Med 60, 207–219 (2022).
2. Brinkmalm, A. et al. SNAP-25 is a promising novel cerebrospinal fluid biomarker for synapse degeneration in Alzheimer's disease. Mol Neurodegener 9, 53 (2014).
3. Öhrfelt, A. et al. The pre-synaptic vesicle protein synaptotagmin is a novel biomarker for Alzheimer's disease. Alz Res Therapy 8, 41 (2016).
4. Kvartsberg, H. et al. The intact postsynaptic protein neurogranin is reduced in brain tissue from patients with familial and sporadic Alzheimer's disease. Acta Neuropathol 137, 89–102 (2019).
5. Kvartsberg, H. et al. Cerebrospinal fluid levels of the synaptic protein neurogranin correlates with cognitive decline in prodromal Alzheimer's disease. Alzheimers Dement 11, 1180–1190 (2015).
6. Ashton, N. J. et al. Plasma levels of soluble TREM2 and neurofilament light chain in TREM2 rare variant carriers. Alzheimers Res Ther 11, 94 (2019).
7. Kleinberger, G. et al. TREM2 mutations implicated in neurodegeneration impair cell surface transport and phagocytosis. Sci Transl Med 6, 243ra86 (2014).
8. Gaetani, L. et al. A new enzyme-linked immunosorbent assay for neurofilament light in cerebrospinal fluid: analytical validation and clinical evaluation. Alzheimers Res Ther 10, 8 (2018).
9. Davidsson, P. et al. Differential increase in cerebrospinal fluid-acetylcholinesterase after treatment with acetylcholinesterase inhibitors in patients with Alzheimer's disease. Neurosci Lett 300, 157–160 (2001).
10. Parnetti, L. et al. Changes in CSF acetyl- and butyrylcholinesterase activity after long-term treatment with AChE inhibitors in Alzheimer's disease. Acta Neurol Scand 124, 122–129 (2011).

Validation

Validation of protocols is described in detail for each assay in the preceding references.

Clinical data

Policy information about clinical studies

All manuscripts should comply with the ICMJE guidelines for publication of clinical research and a completed CONSORT checklist must be included with all submissions.

Clinical trial registration

EU Clinical Trials identifier: 2015-005263-16; USA ClinicalTrials.gov identifier: NCT03069014

Study protocol

Please see Supplementary Note 1 for the trial protocol

Data collection

The trial was initiated at 21 hospitals/clinics located in five European countries: Austria, Germany, Spain, Sweden and the Czech Republic. Participants were enrolled at 18 sites with the first patient randomized in May 2017 and the last participant completing treatment in June 2020. Database lock occurred in November 2020.
Of the 18 clinics which enrolled participants, 2 sites were located in Austria, 4 sites were located in the Czech Republic, 7 sites were located in Germany, 1 site was in Sweden and 4 sites were in Spain. The number of participants recruited by country are as follows: 8 from Austria, 104 from the Czech republic, 48 from Germany, 19 from Sweden and 63 from Spain.
A full list of trial site Principal Investigators and their affiliations can be found in the Supplementary Table 1.

Outcomes

Primary, secondary and exploratory outcome measures were defined in the study protocol and statistical analysis plan. The primary trial outcome was safety (number of EAs/SAEs within the 26-week study period), assessed through adverse event reporting and patient physical evaluations including vital signs, blood pressure, 12-le2d electrocardiogram, MRI, hematology, blood biochemistry, and urinalysis. Clinical safety evaluations consisted of the Columbia Suicide Severity Rating Scale. Secondary biomarker and clinical data were collected and preselected exploratory, longitudinal biomarker and clinical endpoints were also collected. Secondary CSF assays included CSF Aβ40, Aβ42, p-tau181, t-tau and acetylcholinesterase activity. Secondary cognitive outcomes included a composite z-score of a custom NTB consisting of a digit span task, a digit symbol substitution task, a category fluency task, and a controlled oral word association test (COWAT). Prespecified exploratory biomarker outcomes consisted of CSF assays for the

following: the synaptic proteins SNAP25, SYT1, NG; the microglial protein sTREM2, the astrocytic biomarker YKL40, and the neurodegenerative biomarker NfL. Prespecified exploratory imaging studies included sMRI, and 18F-FDG PET. Prespecified exploratory clinical assessments included the MMSE, ADAS-Cog-13, and the CGI. A schematic summary of the time points at which the main measures were obtained is provided in Extended Data Table 2.

Magnetic resonance imaging

Experimental design

Design type: Structural magnetic resonance imaging was used to measure longitudinal grey matter volumes

Design specifications: T1 weighted MRI scans were collected twice: once before treatment, once after 26-weeks (or early discontinuation, if possible).

Behavioral performance measures: NA

Acquisition

Imaging type(s): Structural

Field strength: 1.5-3T

Sequence & imaging parameters:
3 tesla:
Sagittal 3DT1
Method: IR-prepped fast 3D gradient echo (spoiled)
FOV (mm): 256
Acq. Matrix: 256x256
Slice thickness (mm): 1.0
TR (ms): 1800 (Siemens), shortest (Philips), 5-6 (GE)
TE (ms): minimum
flip angle: 8-12 degrees

1.5 Tesla
Same as above except:
TR (ms): 2400 (Siemens), shortest (Philips), 9-14 (GE)

Area of acquisition: whole brain

Diffusion MRI ☐ Used ☒ Not used

Preprocessing

Preprocessing software:
Software used: SPM12 (v7771), CAT12 (12.8.1)
Smoothing kernel (MRI and PET): 6mm isotropic
Detailed information is available in Supplementary Note 2 and pre-processing code has been posted at https://github.com/hayleyshanks/Longitudinal-MRI-PET-preproc

Normalization: MRI and PET data were normalized non-linearly with Geodesic shooting in SPM12 to the clinical trial population template (below).

Normalization template: A custom age- and disease-appropriate population template was created for the clinical trial dataset using Geodesic Shooting in SPM12. All MRI and PET data for the trial was normalized to this custom template space.

Noise and artifact removal: The trial's image analysis unit (IAU) manually inspected MRI scans for quality, and scans with significant motion artifacts or other artifacts (e.g. due to dental work or bad shimming) were rejected. Rejected scans were repeated within 3 weeks of the original scan. During sMRI pre-processing, bias field correction was performed during the creation of midpoint average images, and the quality of midpoint average images was rated automatically in CAT12 (see Supplementary Materials). Low quality images, based on their CAT12 image quality rating, were inspected manually.

Volume censoring: Voxel-wise statistical analyses were restricted to brain regions vulnerable to decline in Alzheimer's disease using explicit masking in CAT12. To define vulnerable brain regions, longitudinal MRI and FDG PET data was aggregated in 54 ADNI participants who met the trial inclusion criteria for age, MMSE score and amyloid abnormality. Voxel-wise t tests were applied throughout the whole brain grey matter to isolate voxels exhibiting significant longitudinal decline in the ADNI cohort for the ADNI MRI and PET data. These t maps were corrected for multiple comparisons (FWER $p < 0.05$), binarized, and used as explicit masks in the clinical trial data analysis.

Statistical modeling & inference

Model type and settings: Statistical analysis of sMRI and 18F-FDG PET data was performed in CAT12 using voxel-wise flexible factorial ANOVA models. Models included drug group (placebo, LM11A-31) and time (baseline, follow-up) as factors. Additionally, we included a subject factor, which controls for participant-specific variables that do not change over time (sex, APOE genotype, trial site).

Flexible factorial models were restricted to the grey matter regions identified to be vulnerable to AD in the ADNI sample (Extended Data Fig. 6). Statistics were mass univariate.

Effect(s) tested

We examined the effect of drug group on voxel-wise grey matter volume (MRI) or glucose metabolism (PET)

Specify type of analysis: ☒ Whole brain ☐ ROI-based ☐ Both

Statistic type for inference
(See Eklund et al. 2016)

Voxel-wise analyses were performed using F and T statistics

Correction

Voxel-wise analyses presented in Fig. 4 and Extended Data Fig. 7 were not corrected for multiple comparisons due to the short, exploratory nature of the study. However, Monte Carlo simulations were used to compare the ratios of hypothesis-consistent to hypothesis-inconsistent effects in MRI and PET data (see results).

Models & analysis

n/a	Involved in the study
☒	☐ Functional and/or effective connectivity
☒	☐ Graph analysis
☒	☐ Multivariate modeling or predictive analysis
